# Comparative Effectiveness of CagriSegma, Semaglutide, Cagrilintide and Tirzepatide in the Management of Overweight and Obesity: A Network Meta‐Analysis of Randomized Clinical Trials

**DOI:** 10.1002/edm2.70248

**Published:** 2026-05-28

**Authors:** Sultan Hamarsheh, Abdel Rahman Jaber, Omar Abu‐Khazneh, Celina R. Andonie, Saleem Majadleh, Anwar Zahran, Hazem Ayesh

**Affiliations:** ^1^ Department of Medicine An‐Najah National University Nablus Palestine; ^2^ School of Medicine University of Jordan Amman Jordan; ^3^ Al‐Quds University Jerusalem Palestine; ^4^ Deaconess Health System Evansville Indiana USA; ^5^ Adjunct Clinical Assistant Professor of Medicine, Indiana University School of Medicine Indianapolis Indiana USA

## Abstract

**Background:**

Obesity is a chronic, progressive disease affecting over 1 billion adults worldwide, linked to serious comorbidities, including diabetes, hypertension, and cardiovascular disease and associated with increased mortality. Achieving clinically meaningful weight loss is critical to reducing cardiometabolic risk. Tirzepatide, semaglutide, cagrilintide and their combination (CagriSema) have demonstrated efficacy in clinical trials; however, no direct head‐to‐head studies have compared all advanced anti‐obesity medications. This network meta‐analysis examines their comparative efficacy and safety.

**Methods:**

We systematically searched PubMed, Scopus and Cochrane Central for randomized controlled trials comparing these medications with placebo in adults with overweight or obesity. Outcomes included changes in percent body weight, absolute weight, waist circumference, BMI, patients achieving ≥ 5% to ≥ 20% weight loss, HDL‐C and safety outcomes (AEs, serious AEs, gastrointestinal AEs and treatment discontinuation). Random‐effects model and network meta‐analysis methods were employed.

**Results:**

Twenty‐five trials involving 12 interventions met the inclusion criteria. Tirzepatide 15 mg resulted in the greatest percent weight reduction (MD −17.97%), followed by CagriSema (MD −17.84%) and semaglutide 7.2 mg (MD −14.66%). At the ≥ 20% weight‐loss threshold, CagriSema demonstrated marked superiority (RR 27.82), followed by tirzepatide 15 mg (RR 23.70). Gastrointestinal adverse events increased with all treatments (RR 1.33–1.91), and treatment discontinuation was highest with semaglutide 7.2 mg (RR 3.09). Serious adverse events remained comparable to placebo across all regimens.

**Conclusion:**

Tirzepatide 15 mg and CagriSema achieve the greatest weight reduction, including ≥ 20% body weight loss. Gastrointestinal adverse events rise with treatment intensity, while serious adverse events remain comparable to placebo. These findings support dual‐pathway and combination incretin therapies as preferred options for patients requiring substantial weight loss. Treatment selection should be individualized based on comorbidity burden, tolerability and weight loss goals, recognizing that ≥ 5% weight loss improves metabolic parameters while ≥ 10% is needed for meaningful comorbidity reduction. Future head‐to‐head trials are needed.

## Graphical abstract

Tirzepatide 15 mg and cagrilintide–semaglutide (CagriSema) achieve the greatest percent body weight reduction (−17.97% and −17.46%, respectively) and the highest rates of ≥ 20% weight loss versus placebo in this network meta‐analysis of 25 randomized trials. Gastrointestinal adverse events rise with treatment intensity, while serious adverse events remain comparable to placebo across all regimens.

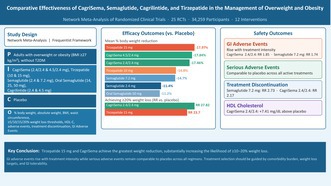

## 1 Introduction

Obesity is a multifactorial disease with a complex aetiology involving biological, psychological, socioeconomic and environmental factors [1]. According to the WHO Global Prevalence report (2025), more than 1.01 billion adults have obesity (BMI ≥ 30 kg/m^2^), and 1.52 billion have overweight (BMI ≥ 25 kg/m^2^), collectively representing 46% of the global adult population [2]. This chronic condition is associated with increased mortality risk, decreased quality of life (QoL) and over 230 comorbidities and complications. These include metabolic disorders such as diabetes, hypertension and dyslipidemia, as well as conditions like obstructive sleep apnoea, non‐alcoholic fatty liver disease and cardiovascular disease [3, 4, 5].

Pharmacological interventions for obesity are used alongside lifestyle modifications, as sustained weight loss through diet and exercise alone is frequently difficult to achieve [6]. Tirzepatide is a long‐acting agent that targets both glucose‐dependent insulinotropic polypeptide (GIP) and glucagon‐like peptide‐1 (GLP‐1) receptors. Semaglutide, a GLP‐1 receptor agonist, is approved for weight management [7, 8]. The SURMOUNT‐5 trial demonstrated that tirzepatide produced significantly greater weight loss than semaglutide in patients with obesity [7]. Cagrilintide, a long‐acting amylin analogue, binds to the calcitonin receptor and all three amylin receptors. Evidence suggests that cagrilintide reduces food intake and promotes weight loss [6]. Additionally, the novel combination of cagrilintide and semaglutide (CagriSema) has demonstrated superior weight‐loss efficacy compared to semaglutide or cagrilintide alone in randomized clinical trials, supporting a synergistic multi‐hormonal approach to obesity management [5, 6].

Although multiple clinical trials have compared tirzepatide and semaglutide, as well as cagrilintide, CagriSema and semaglutide [5, 7], no head‐to‐head studies have directly compared tirzepatide with CagriSema or assessed all advanced anti‐obesity pharmacotherapies within a unified comparative framework. Network meta‐analysis integrates direct and indirect evidence across trials (See Figure S2), enabling simultaneous comparison and ranking of multiple interventions. Accordingly, this study aims to evaluate the efficacy and safety of tirzepatide, semaglutide, cagrilintide and CagriSema using a network meta‐analysis of randomized clinical trials.

## 2 Methods

This research employed a systematic review and network meta‐analysis (NMA) of randomized clinical trials. Methodology and reporting adhered to the Preferred Reporting Items for Systematic Reviews and Meta‐Analyses, including Network Meta‐Analyses (PRISMA‐NMA) guidelines (See Table S12 [9]). The protocol was prospectively registered on the Open Science Framework (OSF; Registration DOI: 10.17605/OSF.IO/BFH6S) [10]. A frequentist framework was applied using the netmeta R package to synthesize both direct and indirect evidence from randomized controlled trials (RCTs) [11].

### 2.1 Eligibility Criteria and Study Selection

Studies were deemed eligible if they met the following criteria: randomized controlled trial design; enrolment of adults (≥ 18 years) with overweight (body mass index [BMI] ≥ 25 kg/m^2^) or obesity (BMI ≥ 30 kg/m^2^) without DM; evaluation of tirzepatide, semaglutide, cagrilintide or the combination of cagrilintide and semaglutide (CagriSema) at any dose with a minimum treatment duration of 12 weeks; comparison with placebo or another intervention of interest; and reporting of at least one of the following outcomes: change in body weight, proportion of participants achieving ≥ 5% weight loss, waist circumference, any adverse event, gastrointestinal adverse events, serious adverse events or treatment discontinuation due to adverse events.

### 2.2 Search Strategy

We systematically searched PubMed, Scopus and the Cochrane Central Register of Controlled Trials to identify relevant studies from database inception to November 29th, 2025. The search terms included: (Tirzepatide OR LY3298176 OR Zepbound OR Mounjaro OR Semaglutide OR Ozempic OR Rybelsus OR Wegovy OR Cagrilintide OR CagriSema OR ‘Cagrilintide + Semaglutide’ OR ‘CagriSema combination’) AND (Obes* OR overweight OR ‘high BMI’ OR ‘body mass index’ OR BMI* OR adiposity) AND (‘randomized controlled trial’ OR RCT OR ‘clinical trial’ OR trial* OR ‘placebo‐controlled’ OR multicenter). No restrictions were applied based on publication type; however, only studies published in English were included, full search strategy is provided in Data S1.

### 2.3 Screening Process

After de‐duplication, search results were screened at the title and abstract levels. Full texts of potentially eligible records were subsequently examined, and reasons for exclusion were documented throughout the screening process. Study selection was performed independently by two reviewers, with disagreements resolved by a third reviewer.

### 2.4 Data Extraction

For each eligible study, data were independently extracted by two reviewers using a standardized and piloted data‐extraction form. The extracted variables comprised baseline study characteristics and participant demographics (e.g., age, sex), anthropometric measures (body weight [kg], body mass index [BMI; kg/m^2^], waist circumference [cm]), glycaemic parameters (glycated haemoglobin [HbA1c, %]), blood pressure (systolic and diastolic) and lipid profile measures (triglycerides and low‐density lipoprotein cholesterol). To ensure consistency across studies, all lipid values were converted to mg/dL using Meta‐Accelerator when necessary [12]. Additional information collected included study design, treatment duration and adverse events. For studies reporting outcomes for multiple intervention arms or subgroups, combined means and standard deviations were calculated according to the Cochrane Handbook for Systematic Reviews of Interventions, and standard errors were derived as appropriate [13].

### 2.5 Risk of Bias Assessment

The methodology of the included study was evaluated using the Cochrane Risk of Bias 2 (RoB 2) tool, which assesses bias in five domains: randomization process, deviations from intended interventions, missing outcome data, outcome measurement and selection of the reported result [14]. Independent assessments were conducted, with disagreements resolved through discussion or by consulting an additional reviewer. The certainty of the evidence was assessed using the Confidence in Network Meta‐Analysis (CINeMA) framework (See Table S9) [15].

### 2.6 Outcomes

The primary outcome was the percentage change in body weight from baseline. Secondary efficacy outcomes were the absolute change in body weight, change in waist circumference, change in body mass index (BMI) and the proportion of participants achieving at least 5%, 10%, 15% and 20% weight loss. Safety outcomes comprised the incidence of any adverse event, serious adverse events, adverse events resulting in treatment discontinuation and gastrointestinal adverse events.

### 2.7 Data Analysis

A network meta‐analysis was conducted to simultaneously compare multiple interventions by integrating evidence from randomized studies, thereby enabling both direct and indirect treatment comparisons, including those not evaluated within individual trials [16]. A random‐effects model was applied to account for between‐study heterogeneity. Treatment effects were estimated as mean differences (MDs) for continuous outcomes and relative risks (RRs) for dichotomous outcomes. In contrast, standardized mean differences (SMDs) were used when outcomes were reported using different measurement scales. Between‐study heterogeneity was assessed using the *τ* ^2^ statistic, the *I* ^2^ statistic and Cochran's Q test [17]. All statistical analyses were conducted in R (RStudio) using the *meta* and *netmeta* packages [11]. Treatments were ranked using *p*‐scores (See Table S8), which quantify the probability that an intervention is among the most effective based on estimated treatment effects [18].

## 3 Results

### 3.1 Literature Search and Study Selection

**FIGURE 1:**
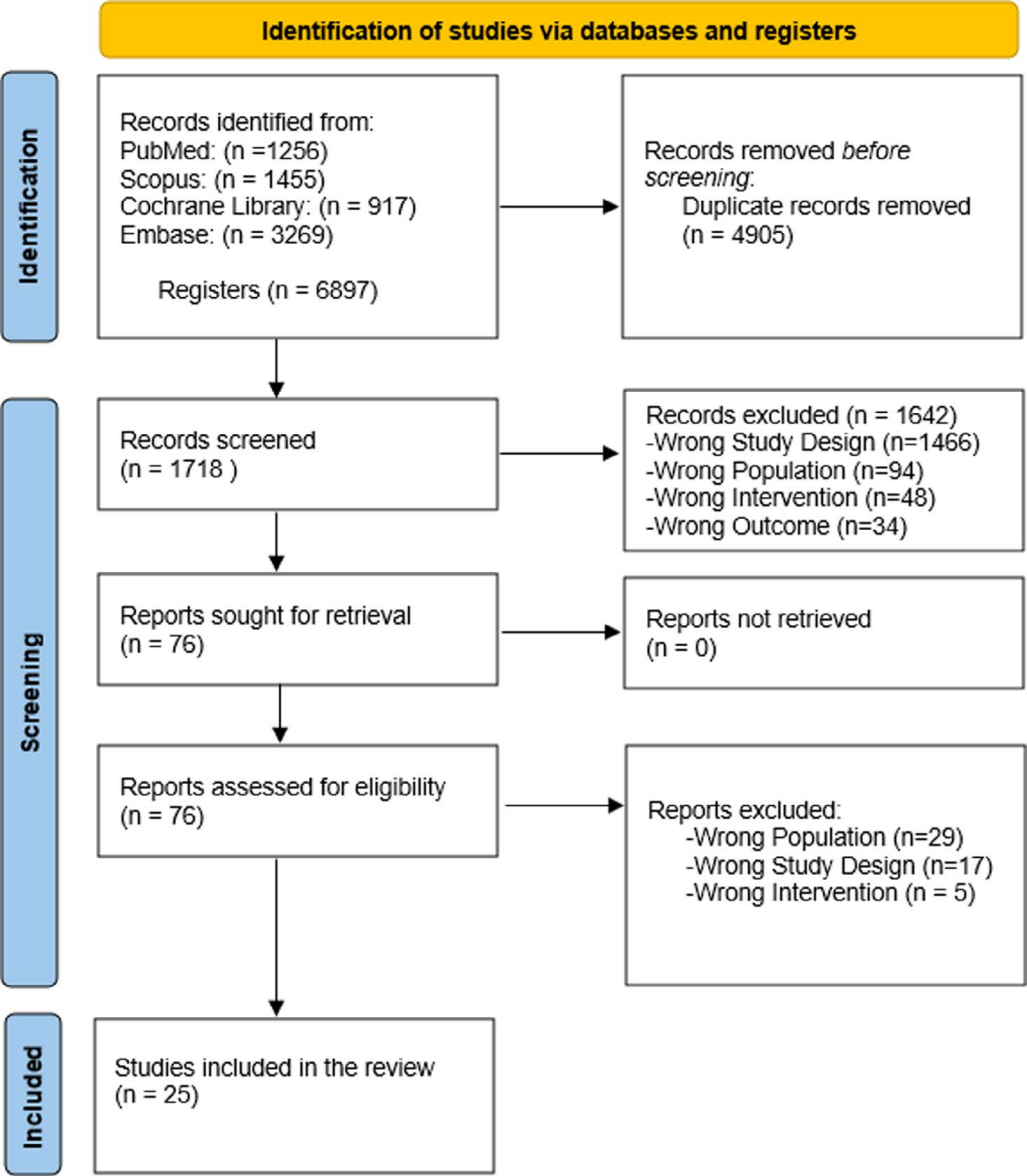
PRISMA flow diagram of the included studies.

A literature search revealed 6897 articles; 4905 duplicates were identified and removed. One thousand six hundred forty‐two were excluded during title and abstract screening. After a thorough full‐text screening of the remaining 76 articles, 25 met our inclusion criteria (Figure 1).

### 3.2 Baseline Characteristics

A total of twenty‐five studies were included in the analysis, comprising 34,259 participants. The mean age of participants was 54 years (SD = 11.2), and the mean body mass index (BMI) was 34.7 kg/m2 (SD = 5.53). All studies were published between 2017 and 2025. Baseline characteristics of the included studies and the participants are summarized in Tables S3 and S4.

### 3.3 Risk of Bias Assessment for the Included Studies

Risk of bias was assessed using the Cochrane risk‐of‐bias framework [14]. Overall, 19 of 25 trials were judged to be at low risk of bias; four raised some concerns, and two were rated high risk. The high‐risk judgements were driven primarily by allocation concealment and blinding domains (blinding of participants/personnel and blinding of outcome assessment). In contrast, selective reporting was consistently rated low risk across the included trials. These domain‐level judgements are presented in Table S5, supporting an overall evidence base that is predominantly low risk, with a small subset requiring cautious interpretation.

### 3.4 Primary Outcomes

#### 3.4.1 Percent Body Weight Change

**FIGURE 2:**
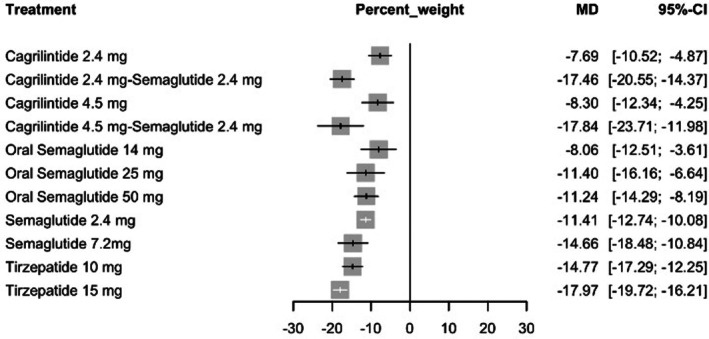
Forest plot for percent body weight change.

Using a random‐effects model, tirzepatide 15 mg was associated with the greatest percent reduction in body weight compared with placebo (MD = −17.97%, 95% CI: −19.72 to −16.21; *p* < 0.0001). Similar magnitudes of reduction were observed with cagrilintide 4.5 mg plus semaglutide 2.4 mg (MD = −17.84%, 95% CI: −23.71 to −11.98; *p* < 0.0001) and cagrilintide 2.4 mg plus semaglutide 2.4 mg (MD = −17.46%, 95% CI: −20.55 to −14.37; *p* < 0.0001). Among semaglutide monotherapy regimens, the 7.2 mg dose demonstrated greater weight reduction (MD = −14.66%, 95% CI: −18.48 to −10.84; *p* < 0.0001) than the standard 2.4 mg dose (MD = −11.41%, 95% CI: −12.74 to −10.08; *p* < 0.0001). Complete comparative estimates are shown in Figure 2.

Substantial heterogeneity was observed (*I* ^2^ = 89.4%, 95% CI: 85.2%–92.4%), and heterogeneity within study designs was statistically significant (Q = 188.29, df = 20, *p* < 0.0001). Global inconsistency testing, however, did not indicate incoherence (Q = 0.57, df = 6, *p* = 0.997). Meta‐regression identified the trial phase as a significant effect modifier (coefficient = −2.71, *p* = 0.030), with larger reductions observed in earlier‐phase trials. Importantly, excluding high‐risk‐of‐bias studies (*I* ^2^ = 89.5%) and small‐sample studies (*I* ^2^ = 89.9%) did not materially alter the magnitude or direction of the effect estimates, and fixed‐effect modelling yielded results that were closely aligned, supporting the overall stability of the findings (See Table S11.1). Ranking probabilities favoured tirzepatide 15 mg (*p*‐score = 0.91), followed by cagrilintide 2.4 mg plus semaglutide 2.4 mg (*p*‐score = 0.872), cagrilintide 4.5 mg plus semaglutide 2.4 mg (*p*‐score = 0.871) and semaglutide 7.2 mg (*p*‐score = 0.69) (See Table S8.1). The network plot, funnel plot, league table and CINeMA are provided in Figures S2.1 and S6.1, Tables S7.1 and S9.1, respectively.

### 3.5 Secondary Outcomes

#### 3.5.1 Change in Waist Circumference

**FIGURE 3:**
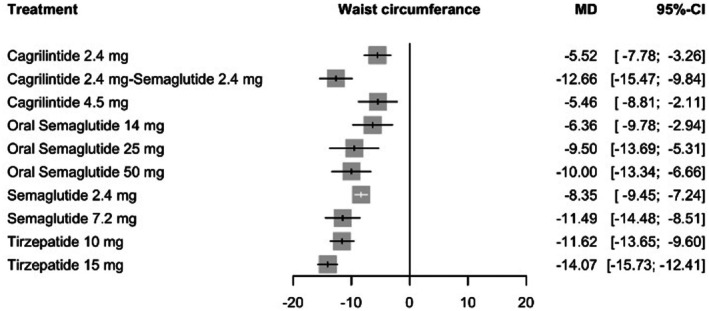
Forest plot for change in waist circumference.

Analysis of waist circumference using a random‐effects framework showed the most pronounced reduction with tirzepatide 15 mg compared with placebo (MD = −14.07 cm, 95% CI: −15.73 to −12.41; *p* < 0.0001). The combination of cagrilintide 2.4 mg plus semaglutide 2.4 mg followed (MD = −12.66 cm, 95% CI: −15.47 to −9.84; *p* < 0.0001), with tirzepatide 10 mg yielding similar reductions (MD = −11.62 cm, 95% CI: −13.65 to −9.60; *p* < 0.0001). Semaglutide 7.2 mg demonstrated a comparable magnitude of decrease (MD = −11.49 cm, 95% CI: −14.48 to −8.51; *p* < 0.0001), whereas the 2.4 mg dose was associated with smaller reductions (MD = −8.35 cm, 95% CI: −9.45 to −7.24; *p* < 0.0001). Detailed estimates are presented in Figure 3.

Substantial heterogeneity was observed (*I* ^2^ = 83.9%, 95% CI: 75.2%–89.6%), with significant heterogeneity within study designs (Q = 93.28, df = 15, *p* < 0.0001). No evidence of global inconsistency was detected (Q = 1.33, df = 5, *p* = 0.932). Meta‐regression did not identify significant effect modifiers (all *p* > 0.05). Importantly, prespecified sensitivity analyses yielded closely comparable effect estimates, with no meaningful changes in magnitude or ranking, reinforcing the robustness and stability of the network estimates (See Table S11.2). Ranking analysis placed tirzepatide 15 mg highest (*p*‐score = 0.96), followed by cagrilintide 2.4 mg plus semaglutide 2.4 mg (*p*‐score = 0.83), tirzepatide 10 mg (*p*‐score = 0.74) and semaglutide 7.2 mg (*p*‐score = 0.73) (See Table S8.2). The network plot, funnel plot, league table and CINeMA are provided in Figures S2.2 and S6.2, Tables S7.2 and S9.2, respectively.

#### 3.5.2 Change in BMI

**FIGURE 4:**
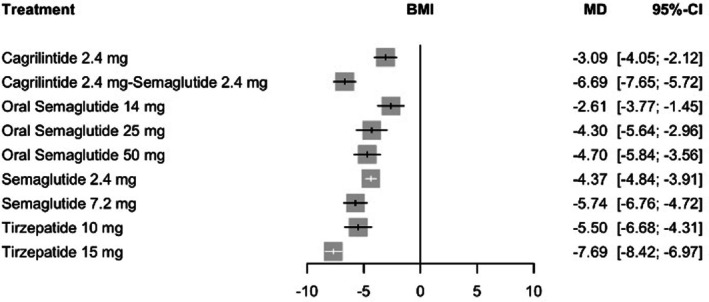
Forest plot for the change in BMI.

For body mass index, the random‐effects model showed the largest mean reduction with tirzepatide 15 mg compared with placebo (MD = −7.69 kg/m^2^, 95% CI: −8.42 to −6.97; *p* < 0.0001). The combination of cagrilintide 2.4 mg plus semaglutide 2.4 mg was associated with the next greatest reduction (MD = −6.69 kg/m^2^, 95% CI: −7.65 to −5.72; *p* < 0.0001), followed by semaglutide 7.2 mg (MD = −5.74 kg/m^2^, 95% CI: −6.76 to −4.72; *p* < 0.0001). Tirzepatide 10 mg yielded a comparable magnitude of reduction (MD = −5.50 kg/m^2^, 95% CI: −6.68 to −4.31; *p* < 0.0001), whereas semaglutide 2.4 mg was associated with more modest changes (MD = −4.37 kg/m^2^, 95% CI: −4.84 to −3.91; *p* < 0.0001). Complete comparative estimates are presented in Figure 4.

Between‐study heterogeneity was substantial (*I* ^2^ = 73.4%, 95% CI: 45.7%–86.9%), and heterogeneity within designs was statistically significant (Q = 26.27, df = 7, *p* = 0.0005). The global test indicated evidence of inconsistency in the network (Q = 11.25, df = 4, *p* = 0.024). Meta‐regression identified systolic blood pressure as a significant effect modifier (coefficient = 0.36, *p* = 0.048), while no other covariates reached statistical significance (all *p* > 0.05). Importantly, sensitivity analyses yielded closely comparable effect estimates and preserved the relative treatment hierarchy, suggesting that the overall pattern of findings remained stable despite observed heterogeneity and evidence of inconsistency (See Table S11.3). According to ranking probabilities, tirzepatide 15 mg had the highest *p*‐score (0.99), followed by cagrilintide 2.4 mg plus semaglutide 2.4 mg (0.87), semaglutide 7.2 mg (0.72) and tirzepatide 10 mg (0.68) (See Table S8.3). The network plot, funnel plot, league table and CINeMA are provided in Figures S2.3 and S6.3, Tables S7.3 and S9.3, respectively.

#### 3.5.3 Absolute Change in Body Weight

**FIGURE 5:**
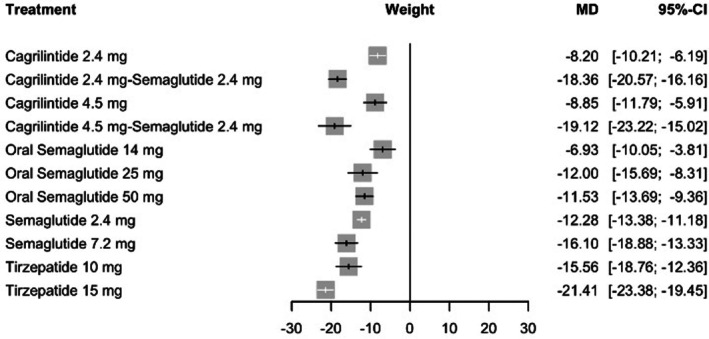
Forest plot for the mean change in body weight.

When absolute body weight change was examined, tirzepatide 15 mg corresponded to the greatest reduction compared with placebo (MD = −21.41 kg, 95% CI: −23.38 to −19.45; *p* < 0.0001). Large reductions were also observed with cagrilintide 4.5 mg plus semaglutide 2.4 mg (MD = −19.12 kg, 95% CI: −23.22 to −15.02; *p* < 0.0001) and cagrilintide 2.4 mg plus semaglutide 2.4 mg (MD = −18.36 kg, 95% CI: −20.57 to −16.16; *p* < 0.0001). Semaglutide 7.2 mg was associated with a substantial decrease (MD = −16.10 kg, 95% CI: −18.88 to −13.33; *p* < 0.0001), whereas the 2.4 mg dose showed smaller reductions (MD = −12.28 kg, 95% CI: −13.38 to −11.18; *p* < 0.0001). Figure 5 provides the full set of estimates.

Heterogeneity was considerable (*I* ^2^ = 89.4%, 95% CI: 85.2%–92.4%), and within‐design heterogeneity was statistically significant (Q = 188.29, df = 20, *p* < 0.0001). No incoherence was detected in the global inconsistency model (Q = 0.57, df = 6, *p* = 0.997). Meta‐regression identified trial phase (coefficient = −2.58, *p* = 0.043), systolic blood pressure (coefficient = 0.99, *p* = 0.004) and diastolic blood pressure (coefficient = 1.33, *p* = 0.021) as significant effect modifiers. No other covariates reached statistical significance (all *p* > 0.05). Sensitivity analyses confirmed the stability of the results (See Table S11.4). Ranking analysis again favoured tirzepatide 15 mg (*p*‐score = 0.98), followed by cagrilintide 2.4 mg plus semaglutide 2.4 mg (0.87), cagrilintide 4.5 mg with semaglutide 2.4 mg (0.83) and semaglutide 7.2 mg (0.70) (See Table S8.3). The network plot, funnel plot, league table and CINeMA are provided in Figures S2.4 and S6.4, Tables S7.4 and S9.4, respectively.

#### 3.5.4 Proportion of Patients Achieving ≥ 5%, ≥ 10%, ≥ 15% and ≥ 20% Weight Loss

For categorical weight loss outcomes, treatment effects increased progressively across higher weight loss thresholds. At ≥ 5% weight loss, oral semaglutide 14 mg and tirzepatide 15 mg demonstrated the highest achievement rates compared to placebo (RR = 4.25, 95% CI: 2.30–7.84 and RR = 3.85, 95% CI: 3.08–4.81, respectively), followed by tirzepatide 10 mg (RR = 3.52, 95% CI: 2.77–4.47). At ≥ 10% weight loss, oral semaglutide 50 mg (RR = 6.27, 95% CI: 3.48–11.27; *p* < 0.001) and cagrilintide 2.4 mg plus semaglutide 2.4 mg (RR = 6.11, 95% CI: 3.93–9.49; *p* < 0.001) showed comparable superiority. For ≥ 15% weight loss, tirzepatide 15 mg achieved the greatest effect (RR = 13.44, 95% CI: 9.44–19.12; *p* < 0.001), with oral semaglutide 14 mg (RR = 15.00, 95% CI: 1.92–117.24; *p* < 0.001) and cagrilintide 2.4 mg plus semaglutide 2.4 mg (RR = 12.68, 95% CI: 7.73–20.79; *p* < 0.001) demonstrating substantial efficacy. At the ≥ 20% weight loss threshold, cagrilintide 2.4 mg plus semaglutide 2.4 mg demonstrated marked superiority (RR = 27.82, 95% CI: 20.94–36.95; *p* < 0.001), followed by tirzepatide 15 mg (RR = 23.70, 95% CI: 18.20–30.86; *p* < 0.001) and tirzepatide 10 mg (RR = 20.18, 95% CI: 15.31–26.59; *p* < 0.001). See Figures S10.1–S10.4.

Substantial heterogeneity was observed at lower thresholds (*I* ^2^ = 83.0%, 82.2%, 66.9% and 0.0% for ≥ 5%, ≥ 10%, ≥ 15% and ≥ 20%, respectively). Tests of heterogeneity within designs yielded significant results for ≥ 5% (Q = 30.95, df = 9, *p* = 0.0003), ≥ 10% (Q = 34.85, df = 10, *p* = 0.0001) and ≥ 15% (Q = 27.74, df = 10, *p* = 0.002), but not for ≥ 20% (Q = 7.91, df = 8, *p* = 0.443). Global inconsistency assessments showed borderline evidence of incoherence at ≥ 5% (Q = 24.57, df = 4, *p* < 0.001) and ≥ 10% (Q = 20.04, df = 5, *p* = 0.001) thresholds but not at ≥ 15% (Q = 5.59, df = 4, *p* = 0.232) or ≥ 20% (Q = 3.08, df = 4, *p* = 0.545). Sensitivity analyses yielded consistent results across all thresholds (See Tables S11.5–S11.8). At ≥ 5%, tirzepatide 15 mg ranked highest (*p*‐score = 0.85), followed by oral semaglutide 14 mg (*p*‐score = 0.84) (See Table S8.5). At ≥ 10%, cagrilintide 2.4 mg plus semaglutide 2.4 mg ranked highest (*p*‐score = 0.73), followed by oral semaglutide 50 mg (*p*‐score = 0.72) (See Table S8.6). At ≥ 15%, tirzepatide 15 mg ranked highest (*p*‐score = 0.81), followed by cagrilintide 2.4 mg plus semaglutide 2.4 mg (*p*‐score = 0.74) (See Table S8.7). At ≥ 20%, cagrilintide 2.4 mg with semaglutide 2.4 mg ranked highest (*p*‐score = 0.97), followed by tirzepatide 15 mg (*p*‐score = 0.86) (See Table S8.8). Complete data for all interventions and thresholds, including network funnel plots, league tables and CINeMA, are provided in the Supporting Information (Figures S2.5–S2.8, S6.5–S6.8 and Tables S7.5–S7.8 and S9.5–S9.8).

### 3.6 Network Geometry

**FIGURE 6:**
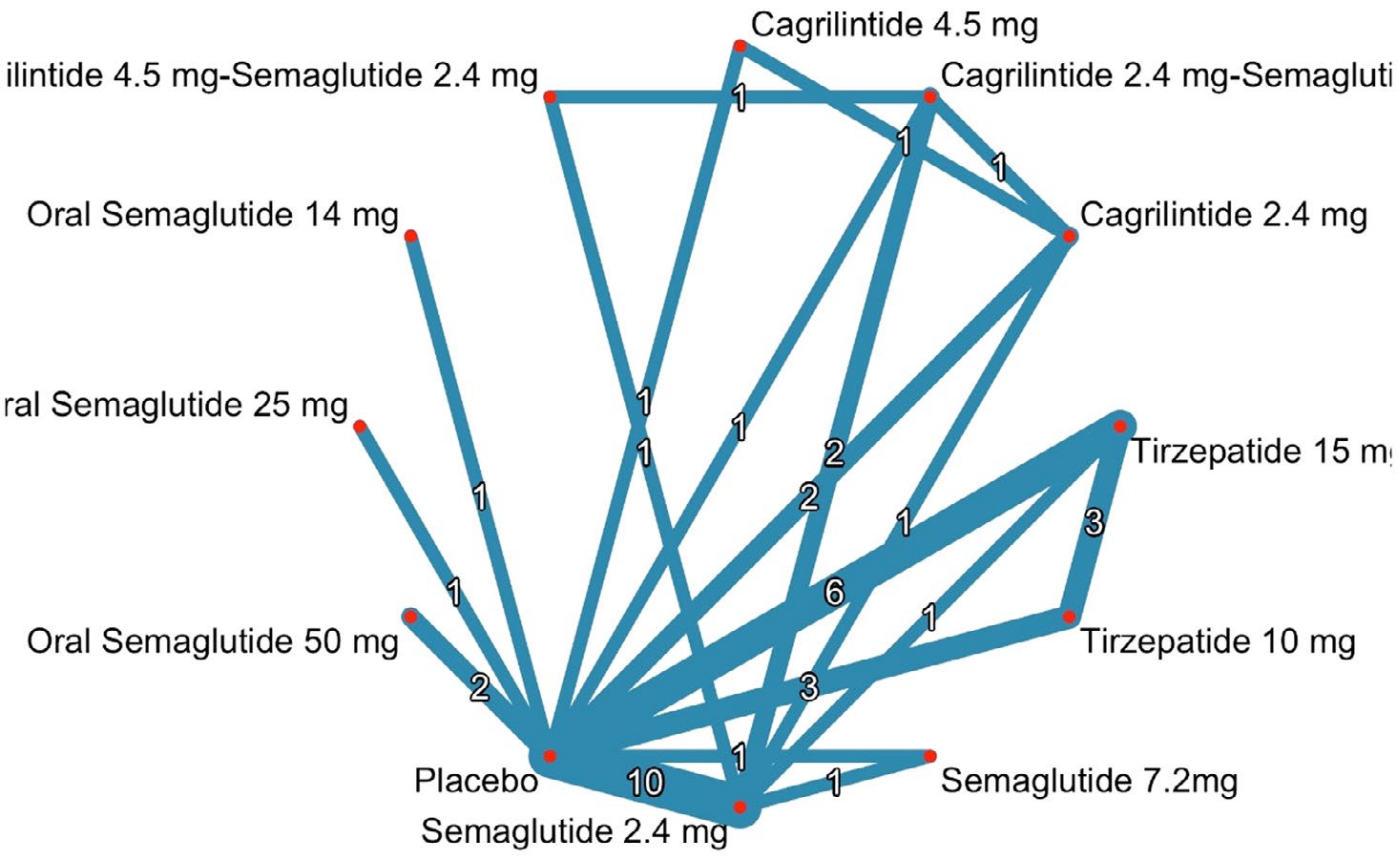
Network plot of change in percent weight.

The network geometry for all included interventions is illustrated in Figure 6. Node size reflects the number of studies involving each treatment, while edge thickness indicates the number of direct comparisons between treatments.

### 3.7 Change in HDL Cholesterol

**FIGURE 7:**
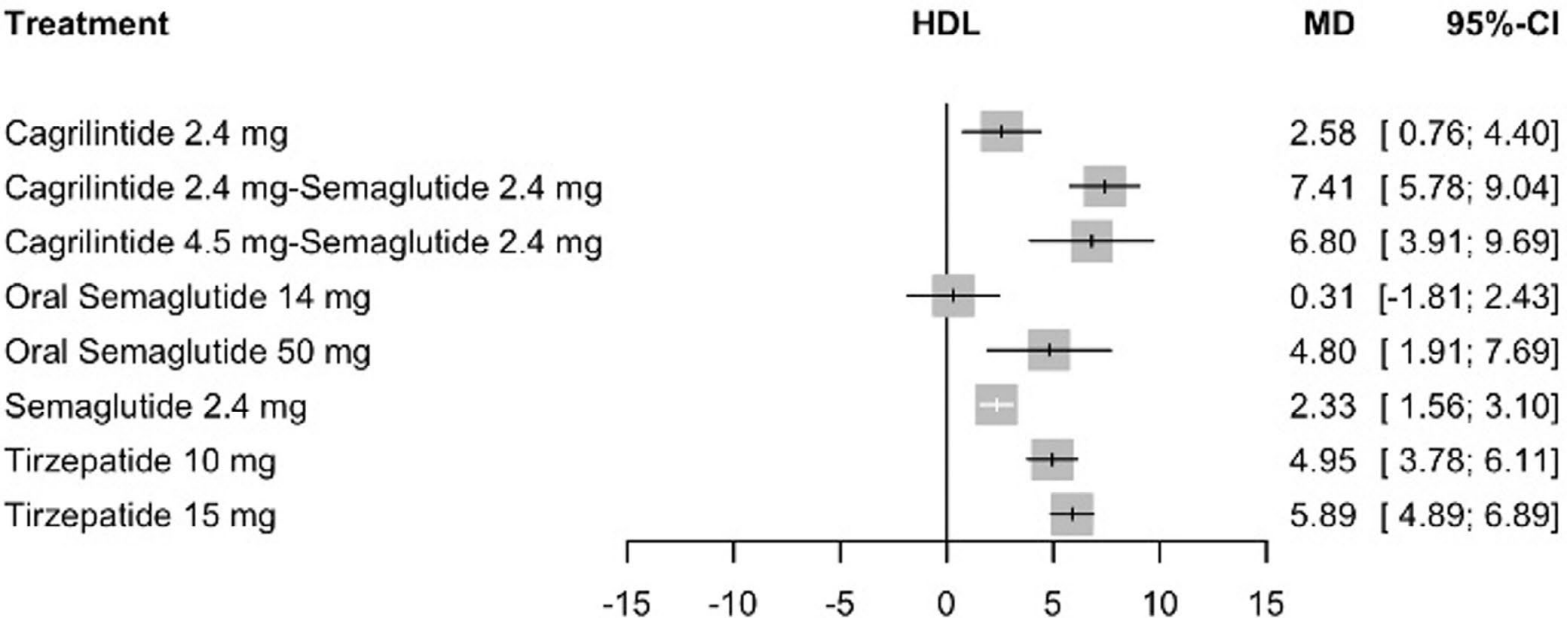
Forest plot for change in HDL cholesterol.

In the network meta‐analysis of HDL cholesterol change, all active interventions except oral semaglutide 14 mg were associated with significant increases compared with placebo. Cagrilintide 2.4 mg plus semaglutide 2.4 mg demonstrated the greatest HDL increase (MD 7.41 mg/dL, 95% CI: 5.78–9.04; *p* < 0.0001), followed by cagrilintide 4.5 mg plus semaglutide 2.4 mg (MD 6.80 mg/dL, 95% CI: 3.91–9.69; *p* < 0.0001) and tirzepatide 15 mg (MD 5.89 mg/dL, 95% CI: 4.89–6.89; *p* < 0.0001). Tirzepatide 10 mg and oral semaglutide 50 mg also demonstrated significant increases (MD 4.95 mg/dL, 95% CI: 3.78–6.11 and MD 4.80 mg/dL, 95% CI: 1.91–7.69, respectively), while cagrilintide 2.4 mg (MD 2.58 mg/dL, 95% CI: 0.76–4.40; *p* = 0.0055) and semaglutide 2.4 mg (MD 2.33 mg/dL, 95% CI: 1.56–3.10; *p* < 0.0001) showed modest but significant increases. Moderate heterogeneity was observed (*I* ^2^ = 68.2%, 95% CI: 40.3%–83.0%), and heterogeneity within study designs was statistically significant (Q = 18.20, df = 6, *p* = 0.006). The global inconsistency test indicated evidence of incoherence between designs (Q = 13.22, df = 4, *p* = 0.010), driven primarily by the tirzepatide dose‐comparison design. Meta‐regression did not identify any significant effect modifiers, with all tested covariates, including age, BMI, baseline weight and lipid parameters, failing to reach statistical significance (all *p* > 0.05). Sensitivity analyses excluding high‐risk‐of‐bias studies and small‐sample studies yielded closely comparable effect estimates, and leave‐one‐out analyses confirmed that no single study materially altered the treatment hierarchy, supporting the robustness of the HDL findings despite moderate heterogeneity (*I* ^2^ range: 46.9%–71.3%) across analyses (See Table S11.13). Ranking analysis favoured cagrilintide 2.4 mg plus semaglutide 2.4 mg the highest (*p*‐score = 0.94), followed by cagrilintide 4.5 mg plus semaglutide 2.4 mg (*p*‐score = 0.84) and tirzepatide 15 mg (*p*‐score = 0.75) (See Table S8.13). The certainty of evidence assessed using the CINeMA framework was rated Low to Very Low across all HDL comparisons (See Table S9.13), driven by substantial heterogeneity and imprecision, consistent with the pattern observed for other continuous efficacy outcomes. Full results are presented in Figure 7. The network plot, funnel plot and league table are provided in Figure S2.13, Tables S6.13 and S7.13, respectively.

### 3.8 Safety Outcomes

#### 3.8.1 Any Adverse Event

**FIGURE 8:**
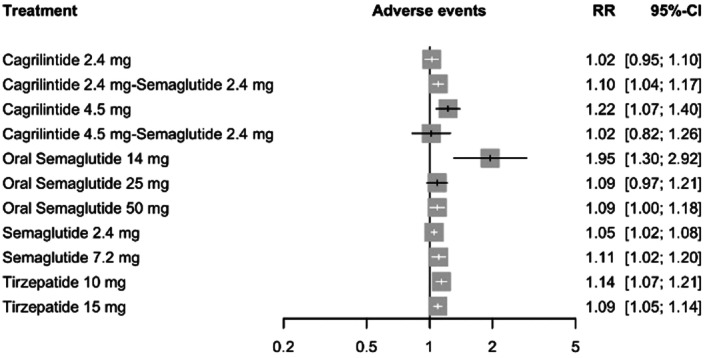
Forest plot for any adverse event.

In the random‐effects analysis of any adverse event (AE), oral semaglutide 14 mg was associated with the highest relative risk compared with placebo (RR = 1.95, 95% CI: 1.30–2.92). Elevated risks were also observed with cagrilintide 4.5 mg (RR = 1.22, 95% CI: 1.07–1.40) and tirzepatide 10 mg (RR = 1.14, 95% CI: 1.07–1.21). In contrast, cagrilintide 2.4 mg (RR = 1.02, 95% CI: 0.95–1.10) and cagrilintide 4.5 mg plus semaglutide 2.4 mg (RR = 1.02, 95% CI: 0.82–1.26) were associated with minimal relative increases. Full estimates are provided in Figure 8.

Moderate heterogeneity was detected (*I* ^2^ = 40.4%, 95% CI: 0.0%–65.5%), and heterogeneity within designs was not statistically significant (Q = 14.68, df = 12, *p* = 0.260). The global inconsistency test suggested possible incoherence, although it did not reach conventional statistical significance (Q = 11.61, df = 6, *p* = 0.071). Meta‐regression identified triglycerides as a significant effect modifier (coefficient = 0.0013, *p* = 0.036), whereas other covariates were not significant (all *p* > 0.05). Sensitivity analyses yielded comparable risk estimates and preserved the relative ordering of interventions, supporting the robustness of the network findings (See Table S11.9). Ranking probabilities indicated the highest AE risk with oral semaglutide 14 mg (*p*‐score = 0.0037), followed by cagrilintide 4.5 mg (*p*‐score = 0.1448) and tirzepatide 10 mg (*p*‐score = 0.2738) (See Table S8.9). The network plot, funnel plot, league table and CINeMA are provided in Figures S2.9 and S6.9, Tables S7.9 and S9.9, respectively.

#### 3.8.2 Serious Adverse Events

**FIGURE 9:**
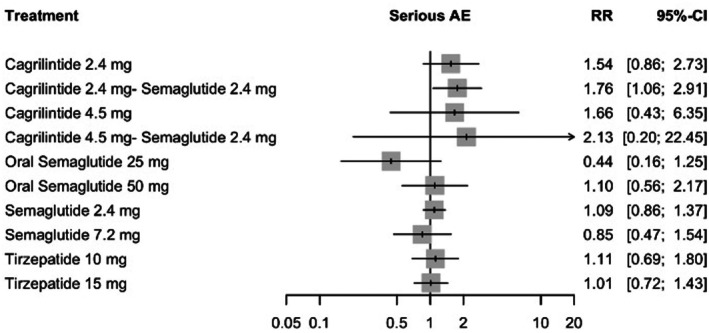
Forest plot for serious adverse events.

For serious adverse events (SAEs), the random‐effects model showed the highest relative risk with cagrilintide 4.5 mg plus semaglutide 2.4 mg compared with placebo (RR = 2.13, 95% CI: 0.20–22.45), followed by cagrilintide 2.4 mg plus semaglutide 2.4 mg (RR = 1.76, 95% CI: 1.06–2.91) and cagrilintide 4.5 mg (RR = 1.66, 95% CI: 0.43–6.35). Most other interventions were associated with risk ratios approximating or below unity, including tirzepatide 15 mg (RR = 1.01, 95% CI: 0.72–1.43), semaglutide 7.2 mg (RR = 0.85, 95% CI: 0.47–1.54) and oral semaglutide 25 mg (RR = 0.44, 95% CI: 0.16–1.25). Complete results are presented in Figure 9.

Moderate heterogeneity was observed (*I* ^2^ = 41.5%, 95% CI: 1.7%–65.2%), and heterogeneity within designs was statistically significant (Q = 27.82, df = 14, *p* = 0.015). However, global inconsistency testing did not indicate incoherence (Q = 3.61, df = 6, *p* = 0.729). Sensitivity analyses produced directionally consistent estimates without material shifts in magnitude, lending support to the stability of the SAE results despite wide confidence intervals for some comparisons (See Table S11.9). Ranking analysis placed cagrilintide 2.4 mg plus semaglutide 2.4 mg highest for SAE risk (*p*‐score = 0.17), whereas oral semaglutide 25 mg ranked lowest (*p*‐score = 0.93) (See Table S8.10). The network plot, funnel plot, league table and CINeMA are provided in Figures S2.10 and S6.10, Tables S7.10 and S9.10, respectively.

#### 3.8.3 GI Adverse Events

**FIGURE 10:**
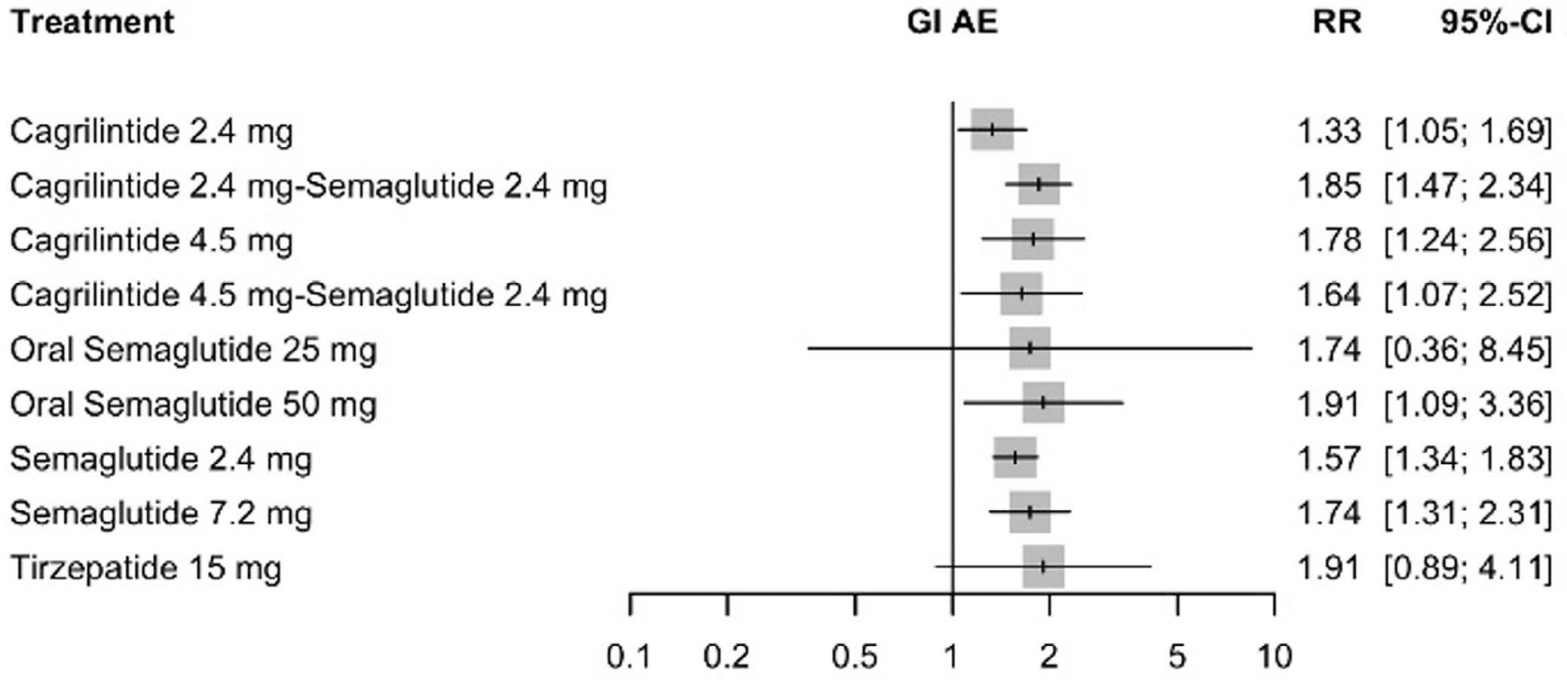
Forest plot for GI adverse events.

In the evaluation of gastrointestinal (GI) adverse events, oral semaglutide 50 mg and tirzepatide 15 mg were associated with the highest relative risks versus placebo (both RR = 1.91; 95% CI: 1.09–3.36 and 0.89–4.11, respectively). Elevated risks were also observed with cagrilintide 2.4 mg plus semaglutide 2.4 mg (RR = 1.85, 95% CI: 1.47–2.34) and cagrilintide 4.5 mg (RR = 1.78, 95% CI: 1.24–2.56). All evaluated interventions were associated with increased GI AE risk, although semaglutide 2.4 mg (RR = 1.57, 95% CI: 1.34–1.83) and cagrilintide 2.4 mg (RR = 1.33, 95% CI: 1.05–1.69) showed comparatively smaller relative increases. Detailed estimates are presented in Figure 10.

Substantial heterogeneity was present (*I* ^2^ = 63.3%, 95% CI: 24.6%–82.1%). While heterogeneity within designs was not statistically significant (Q = 2.54, df = 4, *p* = 0.637), the global inconsistency test indicated evidence of incoherence (Q = 19.26, df = 4, *p* = 0.001). Meta‐regression identified age (coefficient = 0.031, *p* = 0.042) and triglycerides (coefficient = 0.010, *p* = 0.027) as significant effect modifiers; other covariates were not significant (all *p* > 0.05). Notably, sensitivity analyses yielded similar effect estimates and maintained the comparative ranking structure, suggesting that the overall pattern of GI AE risk remained stable despite statistical evidence of inconsistency (See Table S11.11). Cagrilintide 2.4 mg plus semaglutide 2.4 mg ranked highest for GI AE risk (*p*‐score = 0.29), whereas cagrilintide 2.4 mg ranked lowest (*p*‐score = 0.76). The network plot, funnel plot, league table and CINeMA are provided in Figures S2.11 and S6.11, Tables S7.11 and S9.11, respectively.

#### 3.8.4 Adverse Events Leading to Treatment Discontinuation

**FIGURE 11:**
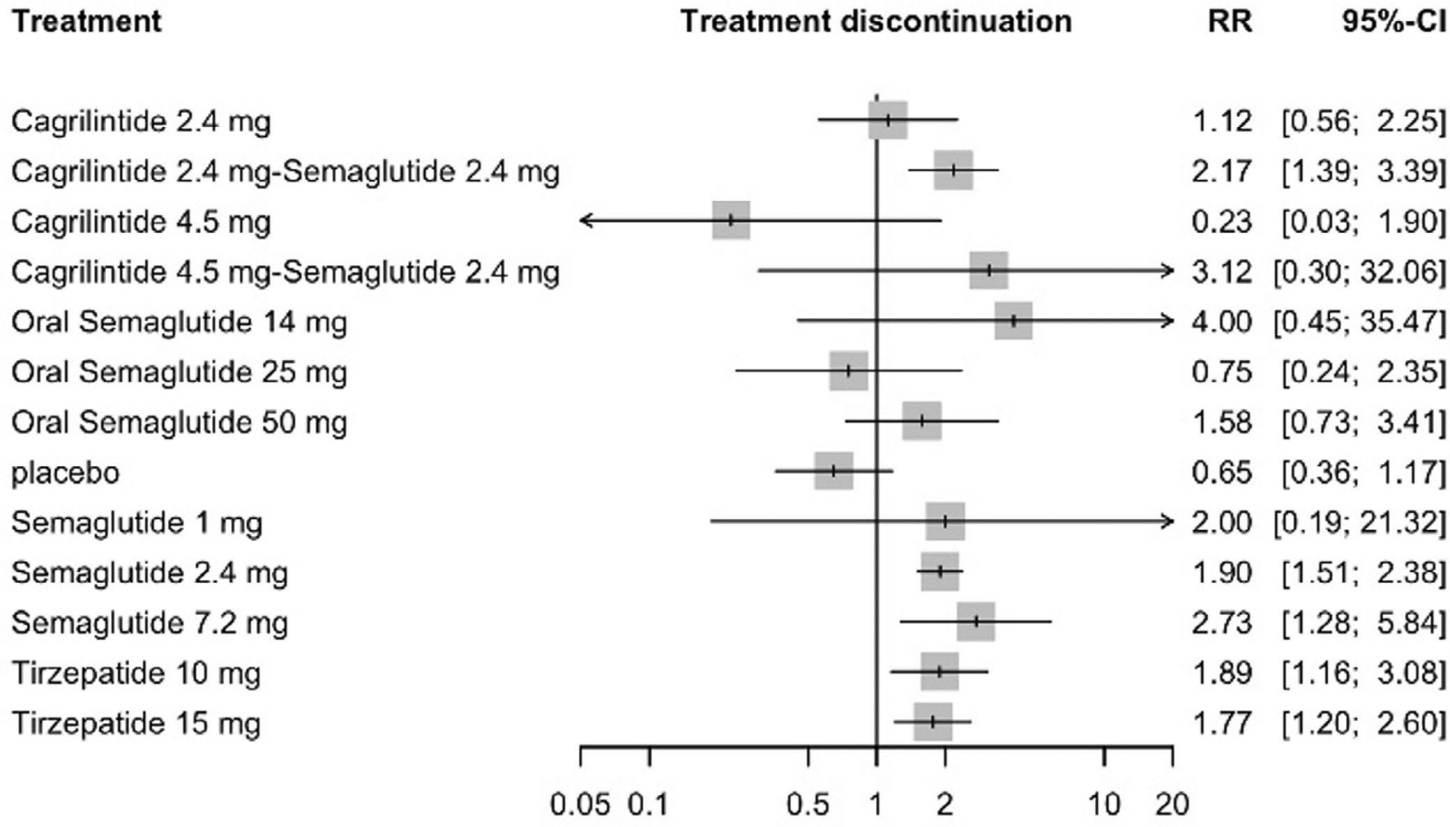
Forest plot for adverse events leading to treatment discontinuation.

In the random‐effects model assessing discontinuation due to adverse events, oral semaglutide 14 mg was associated with the highest relative risk compared with placebo (RR = 4.00, 95% CI: 0.45–35.67), followed by cagrilintide 4.5 mg plus semaglutide 2.4 mg (RR = 3.21, 95% CI: 0.31–33.22) and semaglutide 7.2 mg (RR = 3.09, 95% CI: 1.45–6.59). Increased discontinuation risk was also observed with semaglutide 2.4 mg (RR = 1.98, 95% CI: 1.57–2.50) and tirzepatide 15 mg (RR = 1.99, 95% CI: 1.39–2.85). Cagrilintide 4.5 mg showed a relative risk below unity (RR = 0.23, 95% CI: 0.03–1.94), although the confidence interval was wide. Full results are shown in Figure 11.

Heterogeneity was low (*I* ^2^ = 18.3%, 95% CI: 0.0%–52.8%), and neither within‐design heterogeneity (Q = 15.21, df = 12, *p* = 0.230) nor global inconsistency testing (Q = 5.26, df = 6, *p* = 0.510) suggested violation of model assumptions. Sensitivity analyses yielded closely aligned risk estimates, with no meaningful changes in direction or treatment ranking, supporting the robustness of the drug discontinuation findings (See Table S11.12). Semaglutide 7.2 mg ranked highest for discontinuation risk (*p*‐score = 0.19), whereas cagrilintide 4.5 mg ranked lowest (*p*‐score = 0.95) (See Table S8.12). The network plot, funnel plot, league table and CINeMA are provided in Figures S2.12 and S6.12, Tables S7.12 and S9.12, respectively.

## 4 Discussion

This network meta‐analysis demonstrated that incretin‐based therapies substantially improved weight‐related outcomes in adults with overweight or obesity compared with placebo. Higher‐dose tirzepatide and cagrilintide–semaglutide combination regimens produced the greatest reductions in percent body weight, with tirzepatide 15 mg achieving a mean difference of −17.97% (95% CI: −19.45% to −16.49%) versus placebo. Semaglutide 7.2 mg and the cagrilintide–semaglutide combinations demonstrated comparable efficacy, while semaglutide 2.4 mg achieved robust but smaller effects, and cagrilintide monotherapy showed more modest benefit. Similar hierarchical patterns were observed for absolute weight change, body mass index, waist circumference and clinically meaningful weight‐loss thresholds. Treatment ranking indicated the highest probability of benefit for tirzepatide 15 mg (*p*‐score 0.91), followed by the cagrilintide 2.4 mg plus semaglutide 2.4 mg combination (*p*‐score 0.87) and cagrilintide 4.5 mg plus semaglutide 2.4 mg combination (*p*‐score 0.87). Despite substantial heterogeneity across several outcomes (*I* ^2^ = 89.4% for percent weight change, *I* ^2^ = 83.9% for waist circumference), sensitivity analyses excluding high‐risk‐of‐bias studies and small‐sample‐size trials yielded consistent results, and meta‐regression identified trial phase as a significant modifier of weight outcomes (*p* = 0.030), with earlier‐phase trials showing greater reductions.

The hierarchical efficacy observed in this analysis aligns with the distinct mechanisms by which these therapies act. Tirzepatide, as a dual glucose‐dependent insulinotropic polypeptide (GIP) and glucagon‐like peptide‐1 (GLP‐1) receptor agonist, enhances satiety signalling, delays gastric emptying and improves insulin sensitivity through complementary pathways [7]. The cagrilintide–semaglutide combination leverages dual agonism of the amylin and GLP‐1 receptors, targeting both homeostatic appetite regulation and hedonic food‐reward pathways [19]. In contrast, GLP‐1 receptor agonist monotherapy (semaglutide 2.4 mg and 7.2 mg) achieved substantial but comparatively smaller weight reductions, while cagrilintide monotherapy showed modest effects, consistent with the hypothesis that multi‐receptor approaches provide additive or synergistic benefits. The dose‐dependent effects observed with both semaglutide and tirzepatide further underscore the importance of optimizing therapeutic intensity to maximize clinical benefit.

The magnitude of weight reduction observed with the most effective regimens has important clinical implications for treatment selection and management. Weight loss of at least 5% is associated with improvements in glycaemic control, blood lipids and blood pressure [3]. While reductions exceeding 10% are often necessary to achieve meaningful benefits in patients with obesity‐related comorbidities [3, 20, 21]. In our analysis, treatment effects increased progressively across weight‐loss thresholds. At the ≥ 20% threshold, cagrilintide 2.4 mg–semaglutide 2.4 mg and tirzepatide 15 mg demonstrated marked superiority compared with placebo (RR 27.82, 95% CI: 20.94–36.95 and RR 23.70, 95% CI: 18.20–30.86, respectively), indicating that patients receiving these therapies were nearly 24‐ to 28‐fold more likely to achieve substantial weight loss. Even at the ≥ 15% threshold, tirzepatide 15 mg (RR 13.44, 95% CI: 9.44–19.12) and the cagrilintide combination (RR 12.68, 95% CI: 7.73–20.79) substantially outperformed placebo. However, these highly effective regimens were also associated with increased gastrointestinal adverse events and treatment discontinuation. Cagrilintide 2.4 mg–semaglutide 2.4 mg showed elevated risks for gastrointestinal adverse events (RR 1.86, 95% CI: 1.47–2.34) and treatment discontinuation (RR 2.22, 95% CI: 1.39–3.54), while semaglutide 7.2 mg demonstrated the highest discontinuation rate (RR 3.09, 95% CI: 1.45–6.59). Importantly, serious adverse events did not differ significantly between most active treatments and placebo, with wide confidence intervals limiting definitive conclusions. From a clinical practice perspective, these findings suggest a tiered approach to treatment selection: tirzepatide 15 mg or cagrilintide–semaglutide combinations may be appropriate first‐line options for patients requiring substantial weight loss (≥ 15%–20%) or those with multiple obesity‐related comorbidities. Semaglutide 2.4 mg offers a balance of efficacy and tolerability for patients targeting moderate weight loss (≥ 10%), while lower‐dose regimens may suit those with milder obesity or heightened concerns about gastrointestinal tolerability.

Our findings align with earlier studies on anti‐obesity medications. Previous pairwise meta‐analyses have shown significant, dose‐dependent weight loss with tirzepatide compared to placebo, confirming the effectiveness of dual incretin therapy [19]. Large, randomized trials support these findings. In the SURMOUNT‐5 trial, tirzepatide led to more weight loss than semaglutide in adults with obesity who do not have diabetes [7] The REDEFINE‐1 trial showed that the cagrilintide and semaglutide combination achieved much greater weight loss and a higher rate of reaching a ≥ 20% weight‐loss threshold than either treatment alone [5]. The ranking noted in these trials matches what we found in our network analysis, where multi‐pathway regimens showed the strongest treatment effects. However, previous studies focused on single comparisons and could not assess the relative effectiveness of multiple treatments simultaneously. By combining direct and indirect evidence, this network meta‐analysis provides a clear framework for comparison in a field where direct head‐to‐head data, especially between tirzepatide and the cagrilintide‐semaglutide combination, remain unavailable. Baseline assessment of age and triglyceride levels may help identify patients at higher risk for adverse events, informing monitoring intensity and patient counselling.

This network meta‐analysis, while comprehensive, has several important limitations. Substantial heterogeneity was observed across efficacy outcomes (*I* ^2^ = 89.4% for percent weight change), reflecting variability in study populations, trial duration, lifestyle co‐interventions and outcome measurement methods. To address this, we employed random‐effects modelling, conducted sensitivity analyses excluding high‐risk‐of‐bias and small‐sample‐size studies and performed meta‐regression analyses, which identified trial phase as a significant modifier of weight outcomes. The consistency of treatment rankings across all sensitivity analyses supports the robustness of our comparative conclusions. Network meta‐analysis relies on assumptions of transitivity and consistency; global inconsistency tests demonstrated no major violations, supporting the validity of indirect comparisons. Safety outcomes may be subject to measurement variability due to differences in adverse event definitions and reporting intensity across trials. Reliance on published trial‐level data may introduce publication bias and preclude detailed individual patient‐level subgroup analyses, though meta‐regression identified age and triglycerides as modifiers of adverse event risk. Additionally, most included trials were industry‐sponsored, which may introduce sponsorship bias and should be considered when interpreting the reported outcomes. These limitations are directly reflected in the certainty of evidence assessments performed using the CINeMA framework (See Data S8). Most continuous efficacy outcomes, including change in percent body weight, absolute weight, BMI and waist circumference, were rated Low to Very Low certainty, predominantly driven by the substantial heterogeneity and imprecision described above, with key head‐to‐head comparisons such as CagriSema versus tirzepatide 15 mg rated Very Low, indicating that the true magnitude of differential effects between these regimens remains uncertain. In contrast, the certainty of evidence improved progressively across categorical weight‐loss thresholds: Moderate for ≥ 10% and ≥ 15% outcomes, and High for several clinically important comparisons at the ≥ 20% threshold, including placebo versus tirzepatide 15 mg, placebo versus CagriSema, and CagriSema versus tirzepatide 15 mg directly. Among safety outcomes, any adverse event comparisons were rated Moderate to High certainty, while serious adverse events were Moderate, limited by imprecision reflecting the rarity of events. GI adverse events were similarly rated Moderate, whereas treatment discontinuation was overwhelmingly rated Very Low certainty, driven by within‐study bias and imprecision. Taken together, while the comparative rankings observed in this NMA are consistent and clinically meaningful, the absolute effect estimates for continuous outcomes and treatment discontinuation should be interpreted with caution, and the High certainty evidence at the ≥ 20% weight‐loss threshold represents the most reliable finding of this analysis.

Therefore, future work should move beyond placebo‐controlled designs and focus on large, head‐to‐head randomized trials that directly compare the most effective regimens, including combination approaches. These studies should apply consistent lifestyle co‐interventions, use standardized outcome definitions and include longer follow‐up to clarify the durability of weight loss and its translation into sustained cardiometabolic benefit. In addition, trials would be far more clinically informative if they reported titration protocols in a reproducible way and described adverse events more precisely, particularly severity, timing during dose escalation and the specific reasons for treatment discontinuation to improve the interpretation of tolerability.

## 5 Conclusion

This network meta‐analysis compared 12 incretin‐based interventions for weight management in adults with overweight or obesity across 25 randomized controlled trials. Tirzepatide 15 mg, cagrilintide plus semaglutide combinations and semaglutide 7.2 mg emerged as the most effective treatments for weight reduction, with tirzepatide 15 mg achieving a mean difference of −17.97% body weight compared to placebo and substantially increasing the chance of meaningful weight loss (≥ 20% threshold: RR 23.70, 95% CI: 18.20–30.86). Despite notable heterogeneity among trials, sensitivity analyses confirmed the stability of treatment rankings and network consistency was maintained across most outcomes. Gastrointestinal adverse events were more common with active therapies, while serious adverse events generally did not differ significantly from placebo. Our findings offer valuable evidence for clinicians choosing among available anti‐obesity pharmacotherapies, supporting the use of dual‐pathway and combination incretin therapies for patients needing significant weight reduction. Future head‐to‐head randomized trials with longer follow‐up are necessary to definitively establish the comparative effectiveness, long‐term safety and durability of weight loss with the most promising regimens.

## Author Contributions

**Abdel Rahman Jaber:** conceptualization, methodology, data curation, writing – review and editing, validation. **Hazem Ayesh:** conceptualization, methodology, software, formal analysis, data curation, supervision, project administration, writing – review and editing, writing – original draft, funding acquisition. **Sultan Hamarsheh:** conceptualization, methodology, resources, writing – review and editing, investigation, project administration. **Celina R. Andonie:** conceptualization, methodology, investigation, data curation, writing – review and editing. **Omar Abu‐Khazneh:** conceptualization, methodology, writing – review and editing, data curation. **Saleem Majadleh:** conceptualization, methodology, data curation.

## Funding

The authors have nothing to report.

## Conflicts of Interest

The authors declare no conflicts of interest.

## Supporting information


**Data S1:** Search strategy.
**Figure S2:** Network plots of treatment comparisons.
**Figure S2:** 1: Network Plot of Treatment Comparisons for Changes in Percent Weight.
**Figure S2:** 2: Network Plot of Treatment Comparisons for Changes in Waist Circumference.
**Figure S2:** 3: Network Plot of Treatment Comparisons for Changes in Body Mass Index (BMI).
**Figure S2:** 4: Network Plot of Treatment Comparisons for Absolute Change in Body Weight.
**Figure S2:** 5: Network Plot of Treatment Comparisons for Achieving ≥ 5% weight loss.
**Figure S2:** 6: Network Plot of Treatment Comparisons for Achieving ≥ 10% weight loss.
**Figure S2:** 7: Network Plot of Treatment Comparisons for Achieving ≥ 15% weight loss.
**Figure S2:** 8: Network Plot of Treatment Comparisons for Achieving ≥ 20% weight loss.
**Figure S2:** 9: Network Plot of Any Adverse Event.
**Figure S2:** 10: Network Plot of Serious Adverse Event.
**Figure S2:** 11: Network Plot of GI Adverse Events.
**Figure S2:** 12: Network Plot of Adverse Events Leading to Drug Discontinuation.
**Figure S2:** 13: Network Plot of Change in HDL Cholesterol.
**Data S3:** Baseline characteristics of the included studies.
**Data S4:** Baseline characteristics of the participants.
**Data S5:** Risk of bias table.
**Data S6:** Publication bias (funnel plot).
**Figure S6:** 1: Percent Change in Body Weight.
**Figure S6:** 2: Change in Waist Circumference.
**Figure S6:** 3: Body Mass Index (BMI).
**Figure S6:** 4: Change in Body Weight.
**Figure S6:** 5: Achieving ≥ 5% weight loss.
**Figure S6:** 6: Achieving ≥ 10% weight loss.
**Figure S6:** 7: Achieving ≥ 15% weight loss.
**Figure S6:** 8: Achieving ≥ 20% weight loss.
**Figure S6:** 9: Any Adverse Event.
**Figure S6:** 10: Serious Adverse Event.
**Figure S6:** 11: GI Adverse Event.
**Figure S6:** 12: Adverse Event Leading to Treatment Discontinuation.
**Figure S6:** 13: Change in HDL Cholesterol.
**Table S7:** League Tables.
**Table S7:** 1: Percent Change in Body Weight League.
**Table S7:** 2: Waist Circumference League.
**Table S7:** 3: Body Mass Index (BMI) League.
**Table S7:** 4: Body Weight League.
**Table S7:** 5: Achieving ≥ 5% weight loss League.
**Table S7:** 6: Achieving ≥ 10% weight loss League.
**Table S7:** 7: Achieving ≥ 15% weight loss.
**Table S7:** 8: Achieving ≥ 20% weight loss.
**Table S7:** 9: Any Adverse Events League.
**Table S7:** 10: Serious Adverse Events League.
**Table S7:** 11: GI Adverse Events League.
**Table S7:** 12: Adverse Events Leading to Treatment Discontinuation League.
**Table S7:** 13: Change in HDL Cholesterol League.
**Data S8:** Treatment rankings (by p‐score).
**Data S8:** 1: Percent Change in body weight.
**Data S8:** 3: Change in BMI.
**Data S8:** 4: Absolute change in bodyweight.
**Data S5:** Proportion of patients achieving ≥ 5% weight loss.
**Data S8:** 6: Proportion of patients achieving ≥ 10% weight loss.
**Data S8:** 7: Proportion of patients achieving ≥ 15% weight loss.
**Data S8:** 8: Proportion of patients achieving ≥ 20% weight loss.
**Data S8:** 9: Any Adverse Event.
**Data S8:** 10: Serious Adverse Events.
**Data S8:** 11: GI Adverse Events.
**Data S8:** 12: Adverse Events leading to Treatment Discontinuation.
**Data S8:** 13: Change in HDL Cholesterol.
**Table S9:** CINeMA (Confidence in Network Meta‐Analysis).
**Table S9:** 1: Percent Change in Body Weight League.
**Table S9:** 2: Change in Waist Circumference.
**Table S9:** 3: Change in BMI.
**Table S9:** 4: Absolute Change in body weight.
**Table S9:** 5: Proportion of Patients Achieving > 5% Weight Loss.
**Table S9:** 6: Proportion of Patients Achieving > 10% Weight Loss.
**Table S9:** 7: Proportion of Patients Achieving > 15% Weight Loss.
**Table S9:** 8: Proportion of Patients Achieving > 20% Weight Loss.
**Table S9:** 9: Any Adverse Events.
**Table S9:** 10: Serious Adverse Events.
**Table S9:** 11: GI Adverse Events.
**Table S9:** 12: Adverse Events Leading to Treatment Discontinuation.
**Table S9:** 13: Change in HDL Cholesterol.
**Data S10:** Forest plot.
**Table S11:** Sensitivity Analysis.
**Table S11:** 1: Percent Change in Body Weight Sensitivity Analysis.
**Table S11:** 2: Change in Waist Circumference Sensitivity Analysis.
**Table S11:** 3: Change BMI Sensitivity Analysis.
**Table S11:** 4: Change in Absolute Weight Sensitivity Analysis.
**Table S11:** 5: Proportion of patients achieving ≥ 5% weight loss.
**Table S11:** 6: Proportion of patients achieving ≥ 10% weight loss.
**Table S11:** 6: Proportion of patients achieving ≥ 15% weight loss.
**Table S11:** 7: Proportion of patients achieving ≥ 20% weight loss.
**Table S11:** 8: Change in HDL Sensitivity Analysis.
**Table S11:** 9: Any Adverse Event Sensitivity Analysis.
**Table S11:** 10: Serious Adverse Events Sensitivity Analysis.
**Table S11:** 11: GI Adverse Events Sensitivity Analysis.
**Table S11:** 12: Adverse Events leading to Treatment Discontinuation Sensitivity Analysis.
**Table S11:** 13: Change in HDL Sensitivity Analysis.
**Data S12:** PRISMA Check list.

## Data Availability

The data that support the findings of this study are available from the corresponding author upon reasonable request.
