# Supplementary material for: Comparative Effectiveness of CagriSegma, Semaglutide, Cagrilintide and Tirzepatide in the Management of Overweight and Obesity: A Network Meta‐Analysis of Randomized Clinical Trials
Source: Endocrinol Diabetes Metab. 2026 May 28;9(4):e70248. doi: 10.1002/edm2.70248 (PMC13239642; doi:10.1002/edm2.70248)
Supplement: Supplementary file 1 — Data S1: Search strategy. Figure S2: Network plots of treatment comparisons. Figure S2: 1: Network Plot of Treatment Comparisons for Changes in Percent Weight. Figure S2: 2: Network Plot of Treatment Comparisons for Changes in Waist Circumference. Figure S2: 3: Network Plot of Treatment Comparisons for Changes in Body Mass Index (BMI). Figure S2: 4: Network Plot of Treatment Comparisons for Absolute Change in Body Weight. Figure S2: 5: Network Plot of Treatment Comparisons for Achieving ≥ 5% weight loss. Figure S2: 6: Network Plot of Treatment Comparisons for Achieving ≥ 10% weight loss. Figure S2: 7: Network Plot of Treatment Comparisons for Achieving ≥ 15% weight loss. Figure S2: 8: Network Plot of Treatment Comparisons for Achieving ≥ 20% weight loss. Figure S2: 9: Network Plot of Any Adverse Event. Figure S2: 10: Network Plot of Serious Adverse Event. Figure S2: 11: Network Plot of GI Adverse Events. Figure S2: 12: Network Plot of Adverse Events Leading to Drug Discontinuation. Figure S2: 13: Network Plot of Change in HDL Cholesterol. Data S3: Baseline characteristics of the included studies. Data S4: Baseline characteristics of the participants. Data S5: Risk of bias table. Data S6: Publication bias (funnel plot). Figure S6: 1: Percent Change in Body Weight. Figure S6: 2: Change in Waist Circumference. Figure S6: 3: Body Mass Index (BMI). Figure S6: 4: Change in Body Weight. Figure S6: 5: Achieving ≥ 5% weight loss. Figure S6: 6: Achieving ≥ 10% weight loss. Figure S6: 7: Achieving ≥ 15% weight loss. Figure S6: 8: Achieving ≥ 20% weight loss. Figure S6: 9: Any Adverse Event. Figure S6: 10: Serious Adverse Event. Figure S6: 11: GI Adverse Event. Figure S6: 12: Adverse Event Leading to Treatment Discontinuation. Figure S6: 13: Change in HDL Cholesterol. Table S7: League Tables. Table S7: 1: Percent Change in Body Weight League. Table S7: 2: Waist Circumference League. Table S7: 3: Body Mass Index (BMI) League. Table S7: 4: Body Weight League. Table S7: 5: Achievi [file EDM2-9-e70248-s001.docx]

Table of Contents

[Supplement S1. Search strategy 5](#_Toc225636744)

[Figure S2.1: Network Plot of Treatment Comparisons for Changes in Percent Weight 7](#_Toc225636745)

[Figure S2.4: Network Plot of Treatment Comparisons for Absolute Change in Body Weight 10](#_Toc225636746)

[Figure S2.8: Network Plot of Treatment Comparisons for Achieving ≥ 20 % weight loss 13](#_Toc225636747)

[Figure S2.10: Network Plot of Serious Adverse Event 16](#_Toc225636748)

[Figure S2.11: Network Plot of GI Adverse Events 17](#_Toc225636749)

[Figure S2.12: Network Plot of Adverse Events Leading to Drug Discontinuation 17](#_Toc225636750)

[Figure S2.13: Network Plot of Change in HDL Cholesterol 19](#_Toc225636751)

[Supplement S3 Baseline characteristics of the included studies 19](#_Toc225636752)

[Supplement S4. Baseline characteristics of the participants 24](#_Toc225636753)

[Abbreviations: 25](#_Toc225636754)

[Supplement S5. Risk of bias table 26](#_Toc225636755)

[Supplement S6. Publication bias (funnel plot) 32](#_Toc225636756)

[Figure S6.1: Percent Change in Body Weight 32](#_Toc225636757)

[Figure S6.2: Change in Waist Circumference 33](#_Toc225636758)

[Figure S6.3: Body Mass Index (BMI) 34](#_Toc225636759)

[Figure S6.4: Change in Body Weight 35](#_Toc225636760)

[Figure S6.8: Achieving ≥ 20% weight loss 40](#_Toc225636761)

[Figure S6.9: Any Adverse Event 41](#_Toc225636762)

[Figure S6.10: Serious Adverse Event 42](#_Toc225636763)

[Figure S6.11: GI Adverse Event 43](#_Toc225636764)

[Figure S6.12: Adverse Event Leading to Treatment Discontinuation 44](#_Toc225636765)

[Figure S6.13: Change in HDL Cholesterol 45](#_Toc225636766)

[Supplement S7: League Tables 46](#_Toc225636767)

[Table S7.1: Percent Change in Body Weight League 46](#_Toc225636768)

[Table S7.2: Waist Circumference League 47](#_Toc225636769)

[Table S7.3: Body Mass Index (BMI) League 47](#_Toc225636770)

[Table S7.4: Body Weight League 48](#_Toc225636771)

[Table S7.5: Achieving ≥ 5% weight loss League 49](#_Toc225636772)

[Table S7.6: Achieving ≥ 10% weight loss League 49](#_Toc225636773)

[Table S7.7: Achieving ≥ 15% weight loss 50](#_Toc225636774)

[Table S7.8: Achieving ≥ 20% weight loss 50](#_Toc225636775)

[Table S7.9: Any Adverse Events League 51](#_Toc225636776)

[Table S7.10: Serious Adverse Events League 52](#_Toc225636777)

[Table S7.11: GI Adverse Events League 52](#_Toc225636778)

[Table S7.12: Adverse Events Leading to Treatment Discontinuation League 53](#_Toc225636779)

[Table S7.13: Change in HDL Cholesterol League 54](#_Toc225636780)

[Supplement S8: Treatment rankings (by P-score): 55](#_Toc225636781)

[S8.1: Percent Change in body weight 55](#_Toc225636782)

[S8.3: Change in BMI 55](#_Toc225636783)

[S8.4: Absolute change in bodyweight 56](#_Toc225636784)

[S5: Proportion of patients achieving ≥ 5% weight loss 56](#_Toc225636785)

[S8.6: Proportion of patients achieving ≥ 10% weight loss 56](#_Toc225636786)

[S8.7: Proportion of patients achieving ≥ 15% weight loss 57](#_Toc225636787)

[S8.8: Proportion of patients achieving ≥ 20% weight loss 57](#_Toc225636788)

[S8.9: Any Adverse Event 57](#_Toc225636789)

[S8.10: Serious Adverse Events 58](#_Toc225636790)

[S8.11: GI Adverse Events 58](#_Toc225636791)

[S8.12: Adverse Events leading to treatment discontinuation 58](#_Toc225636792)

[S8.12: Change in HDL Cholesterol 59](#_Toc225636793)

[Supplement S9. CINeMA (Confidence in Network Meta-Analysis) 60](#_Toc225636794)

[Table S9.1: Percent Change in Body Weight League 60](#_Toc225636795)

[Table S9.2: Change in Waist Circumference 61](#_Toc225636796)

[Table S9.3: Change in BMI 62](#_Toc225636797)

[Table S9.4: Absolute Change in body weight 63](#_Toc225636798)

[Table S9.5: Proportion of Patients Achieving >5% Weight Loss 64](#_Toc225636799)

[Table S9.6: Proportion of Patients Achieving >10% Weight Loss 65](#_Toc225636800)

[Table S9.7: Proportion of Patients Achieving >15% Weight Loss 65](#_Toc225636801)

[Table S9.8: Proportion of Patients Achieving >20% Weight Loss 67](#_Toc225636802)

[Table S9.9: Any Adverse Events 68](#_Toc225636803)

[Table S9.10: Serious Adverse Events 69](#_Toc225636804)

[Table S9.11: GI Adverse Events 70](#_Toc225636805)

[Table S9.12: Adverse Events Leading to Treatment Discontinuation 71](#_Toc225636806)

[Table S9.12: Change in HDL Cholesterol 73](#_Toc225636807)

[Supplement S10. Forest plot. 74](#_Toc225636808)

[Supplement S11. Sensitivity Analysis 78](#_Toc225636809)

[Table S11.1: Percent Change in Body Weight Sensitivity Analysis 78](#_Toc225636810)

[Table S11.2: Change in Waist Circumference Sensitivity Analysis 79](#_Toc225636811)

[Table S11.3: Change BMI Sensitivity Analysis 79](#_Toc225636812)

[Table S11.4: Change in Absolute Weight Sensitivity Analysis 80](#_Toc225636813)

[Table S11.5: Proportion of patients achieving ≥ 5% weight loss 80](#_Toc225636814)

[Table S11.6: Proportion of patients achieving ≥ 10% weight loss 81](#_Toc225636815)

[Table S11.7: Proportion of patients achieving ≥ 15% weight loss 81](#_Toc225636816)

[Table S11.8: Proportion of patients achieving ≥ 20% weight loss 82](#_Toc225636817)

[Table S11.9: Change in HDL Sensitivity Analysis 82](#_Toc225636818)

[Table S11.10: Any Adverse Event Sensitivity Analysis 82](#_Toc225636819)

[Table S11.11: Serious Adverse Events Sensitivity Analysis 83](#_Toc225636820)

[Table S11.12: GI Adverse Events Sensitivity Analysis 83](#_Toc225636821)

[Table S11.13: GI Adverse Events Sensitivity Analysis 84](#_Toc225636822)

[S12. PRISMA Check list. 85](#_Toc225636823)

# **Supplement S1. Search strategy**

From inception to Nov 29^th,^ 2025.

**Pubmed**

(Tirzepatide OR LY3298176 OR Zepbound OR Mounjaro OR Semaglutide OR Ozempic OR Rybelsus OR Wegovy OR Cagrilintide OR CagriSema OR "Cagrilintide + Semaglutide" OR "CagriSema combination") AND (Obes* OR overweight OR "high BMI" OR "body mass index" OR BMI* OR adiposity) AND ("randomized controlled trial" OR RCT OR "clinical trial" OR trial* OR "placebo-controlled" OR multicenter)

**Web of Science**

(Tirzepatide OR LY3298176 OR Zepbound OR Mounjaro OR Semaglutide OR Ozempic OR Rybelsus OR Wegovy OR Cagrilintide OR CagriSema OR "Cagrilintide + Semaglutide" OR "CagriSema combination") AND (Obes* OR overweight OR "high BMI" OR "body mass index" OR BMI* OR adiposity) AND ("randomized controlled trial" OR RCT OR "clinical trial" OR trial* OR "placebo-controlled" OR multicenter)

**Scopus**

(Tirzepatide OR LY3298176 OR Zepbound OR Mounjaro OR Semaglutide OR Ozempic OR Rybelsus OR Wegovy OR Cagrilintide OR CagriSema OR "Cagrilintide + Semaglutide" OR "CagriSema combination") AND (Obes* OR overweight OR "high BMI" OR "body mass index" OR BMI* OR adiposity) AND ("randomized controlled trial" OR RCT OR "clinical trial" OR trial* OR "placebo-controlled" OR multicenter)

**Cochrane**

(Tirzepatide OR LY3298176 OR Zepbound OR Mounjaro OR Semaglutide OR Ozempic OR Rybelsus OR Wegovy OR Cagrilintide OR CagriSema OR "Cagrilintide + Semaglutide" OR "CagriSema combination") AND (Obes* OR overweight OR "high BMI" OR "body mass index" OR BMI* OR adiposity) AND ("randomized controlled trial" OR RCT OR "clinical trial" OR trial* OR "placebo-controlled" OR multicenter)

Supplement S2. Network plots of treatment comparisons

# **Figure S2.1: Network Plot of Treatment Comparisons for Changes in Percent Weight**


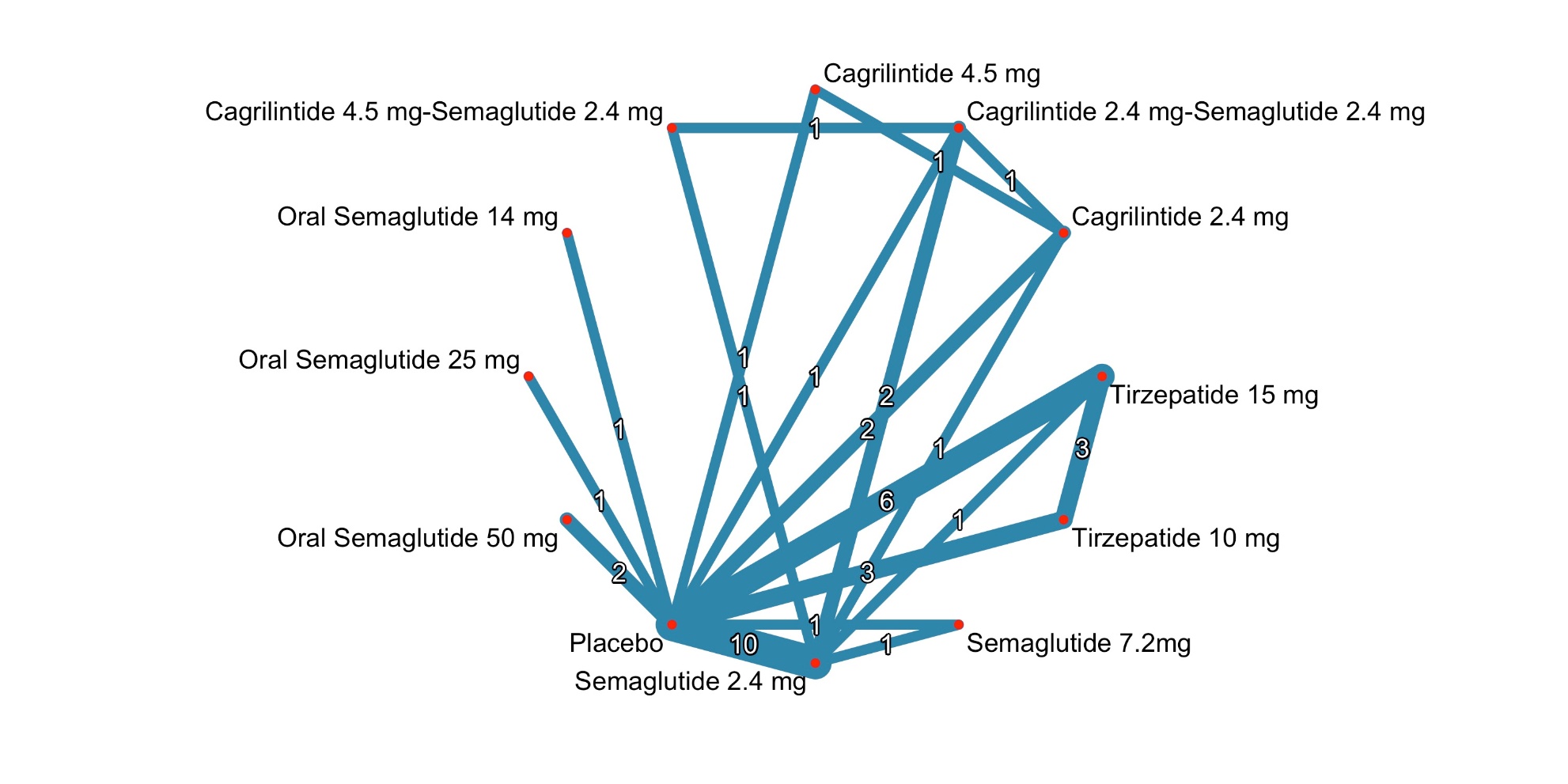


**Figure S2.2: Network Plot of Treatment Comparisons for Changes in Waist Circumference**


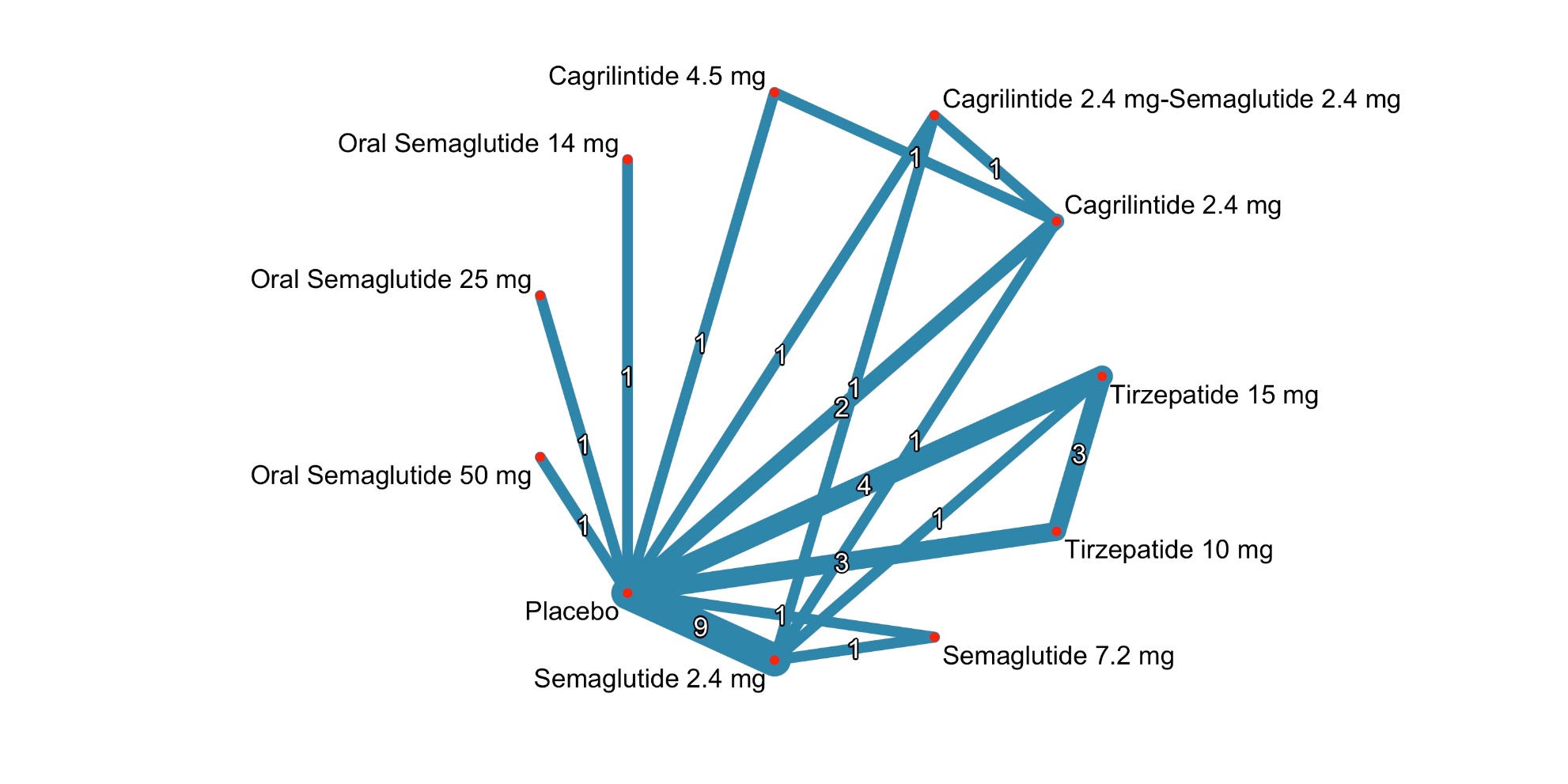


**Figure S2.3: Network Plot of Treatment Comparisons for Changes in Body Mass Index (BMI)**


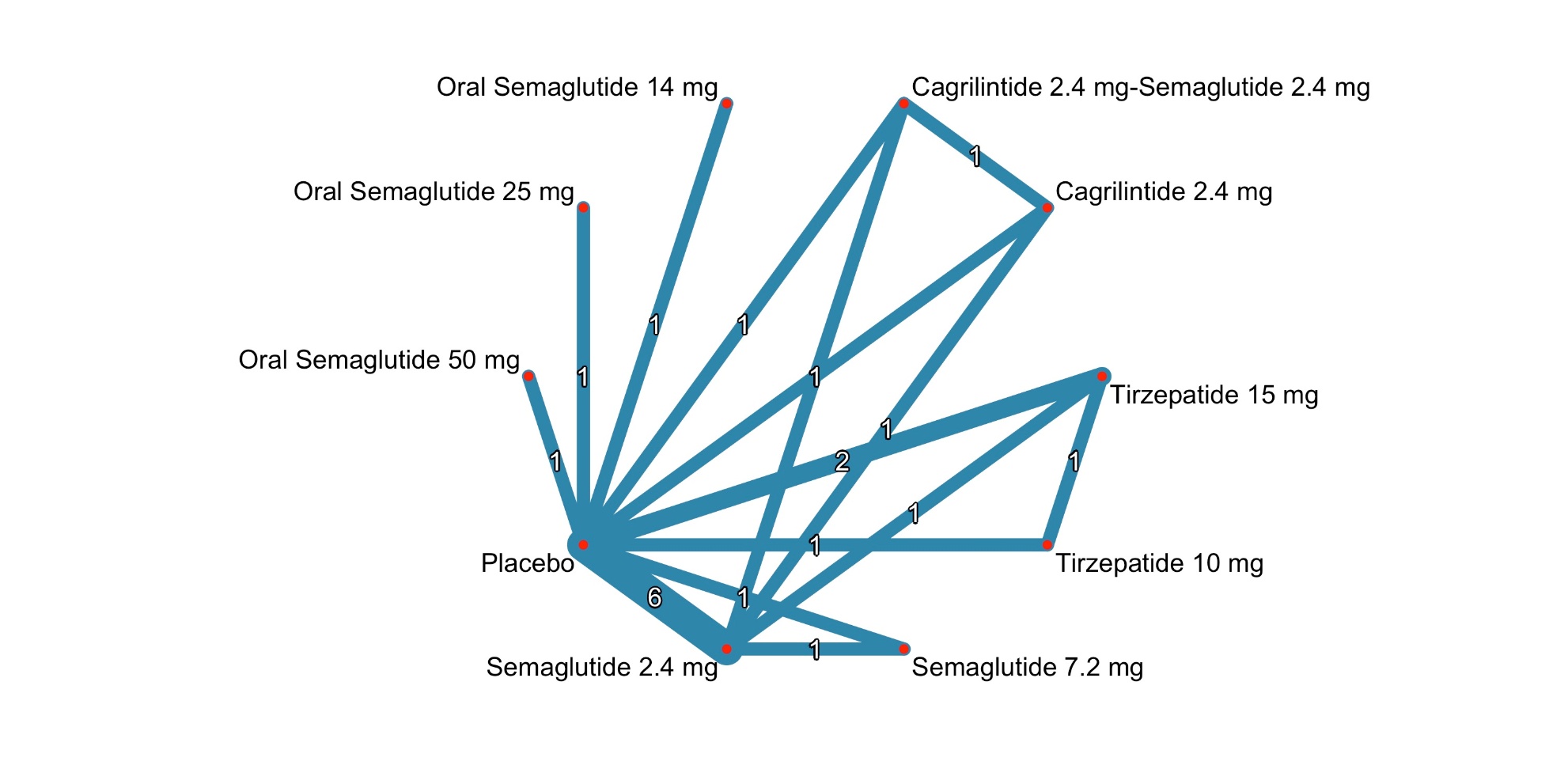


## **Figure S2.4: Network Plot of Treatment Comparisons for Absolute Change in Body Weight**


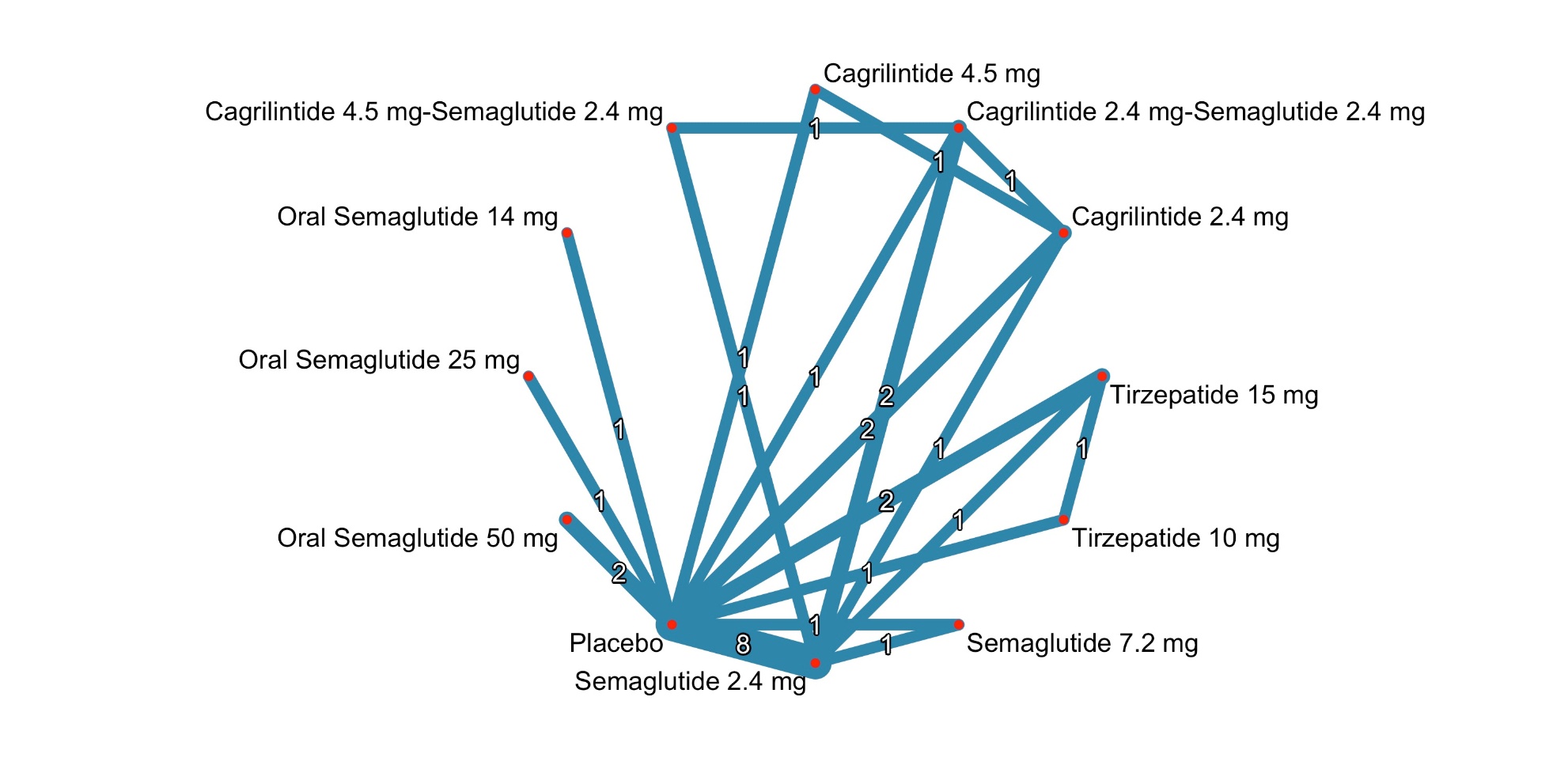


**Figure S2.5: Network Plot of Treatment Comparisons for Achieving ≥ 5% weight loss**


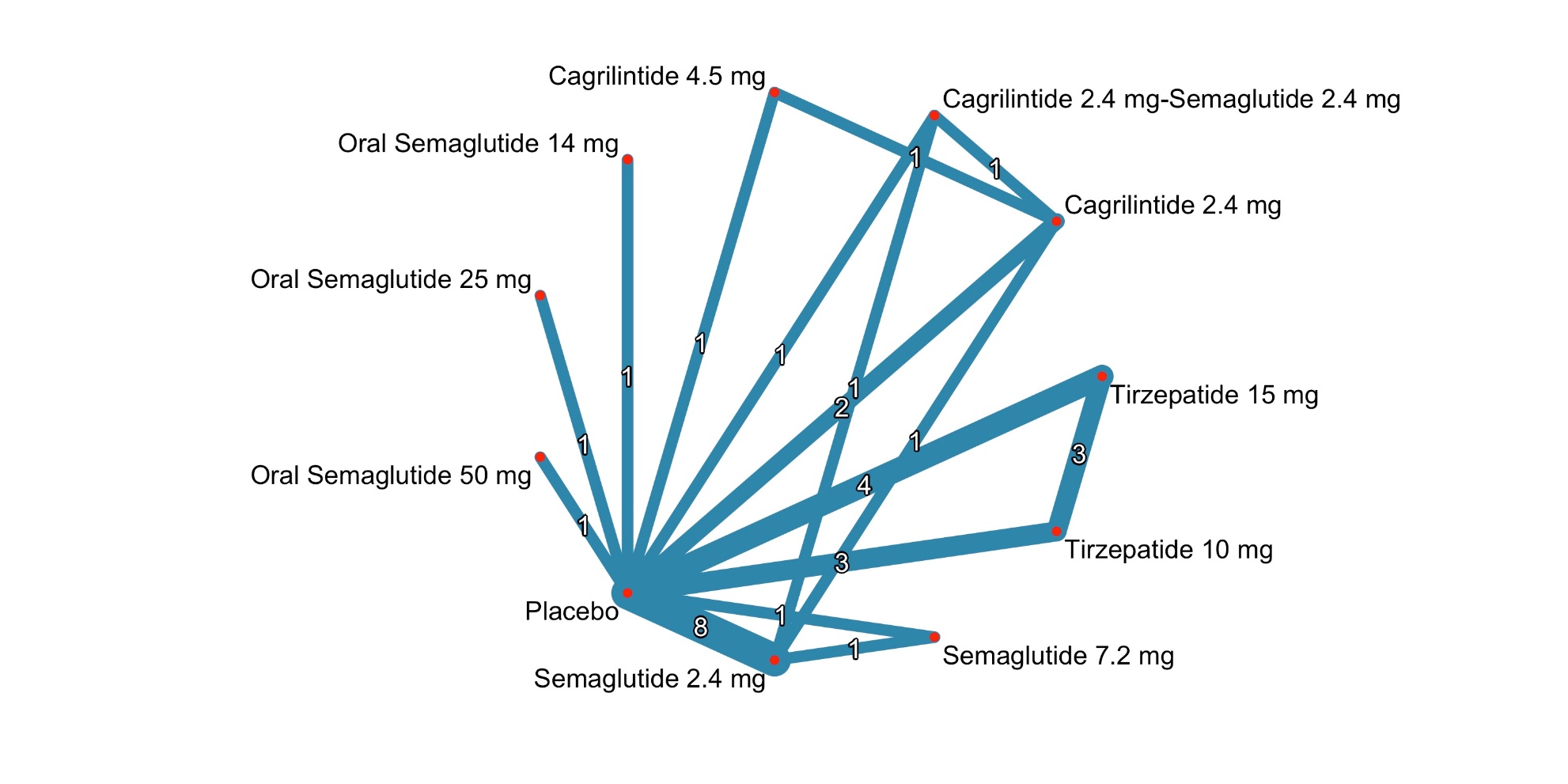


**Figure S2.6: Network Plot of Treatment Comparisons for Achieving ≥ 10 % weight loss**
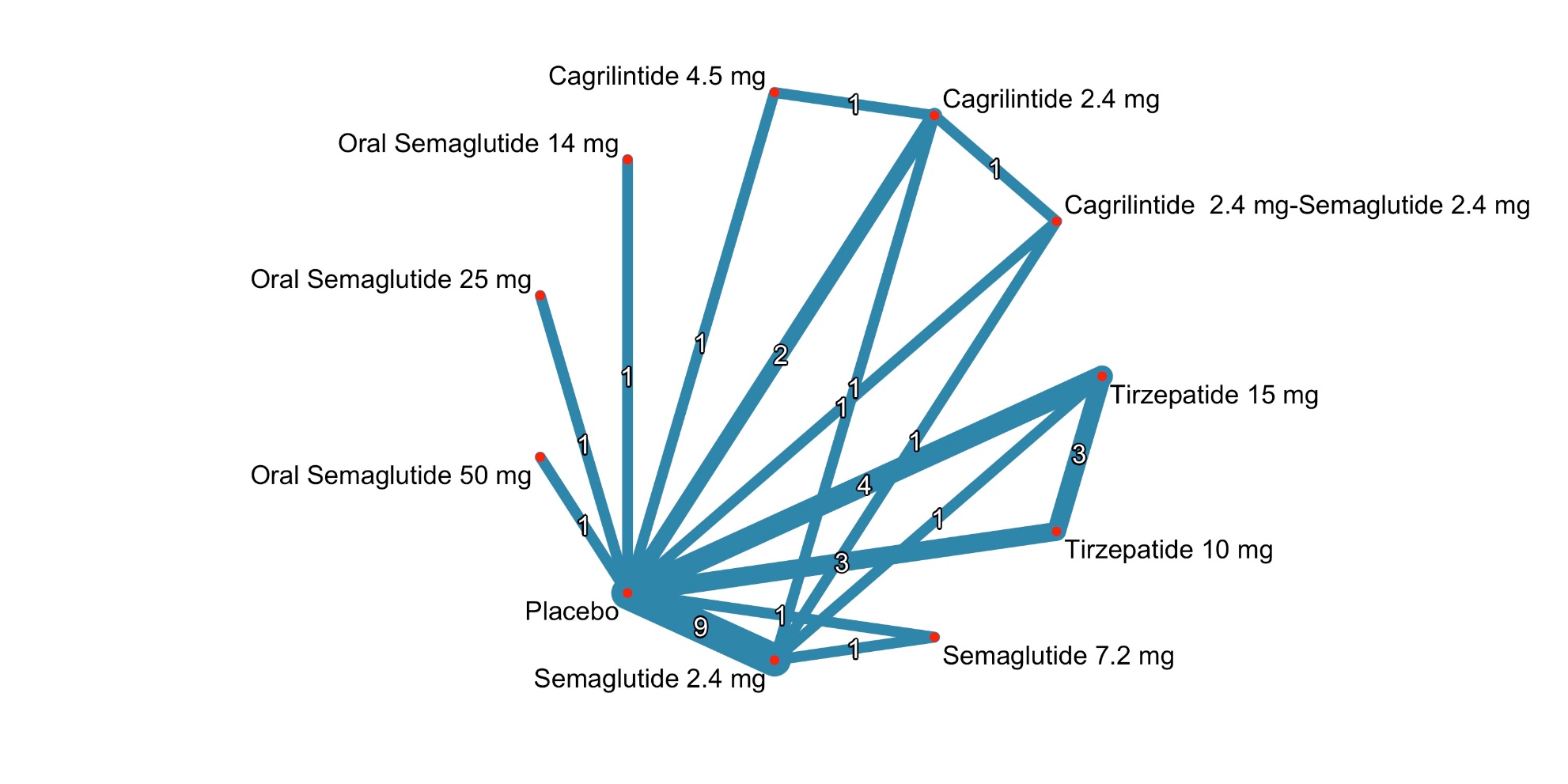


**Figure S2.7: Network Plot of Treatment Comparisons for Achieving ≥ 15 % weight loss**


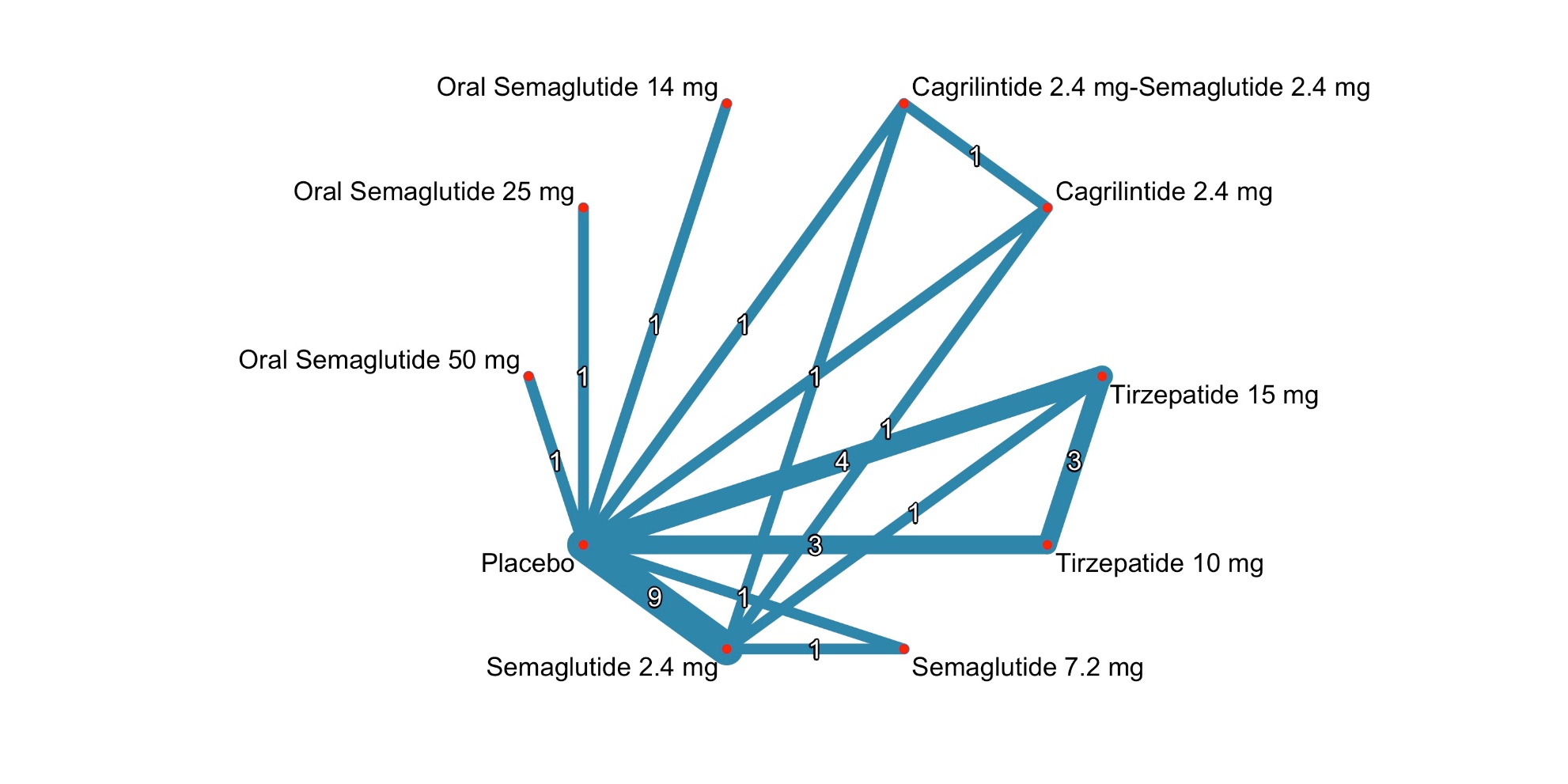


# **Figure S2.8: Network Plot of Treatment Comparisons for Achieving ≥ 20 % weight loss**


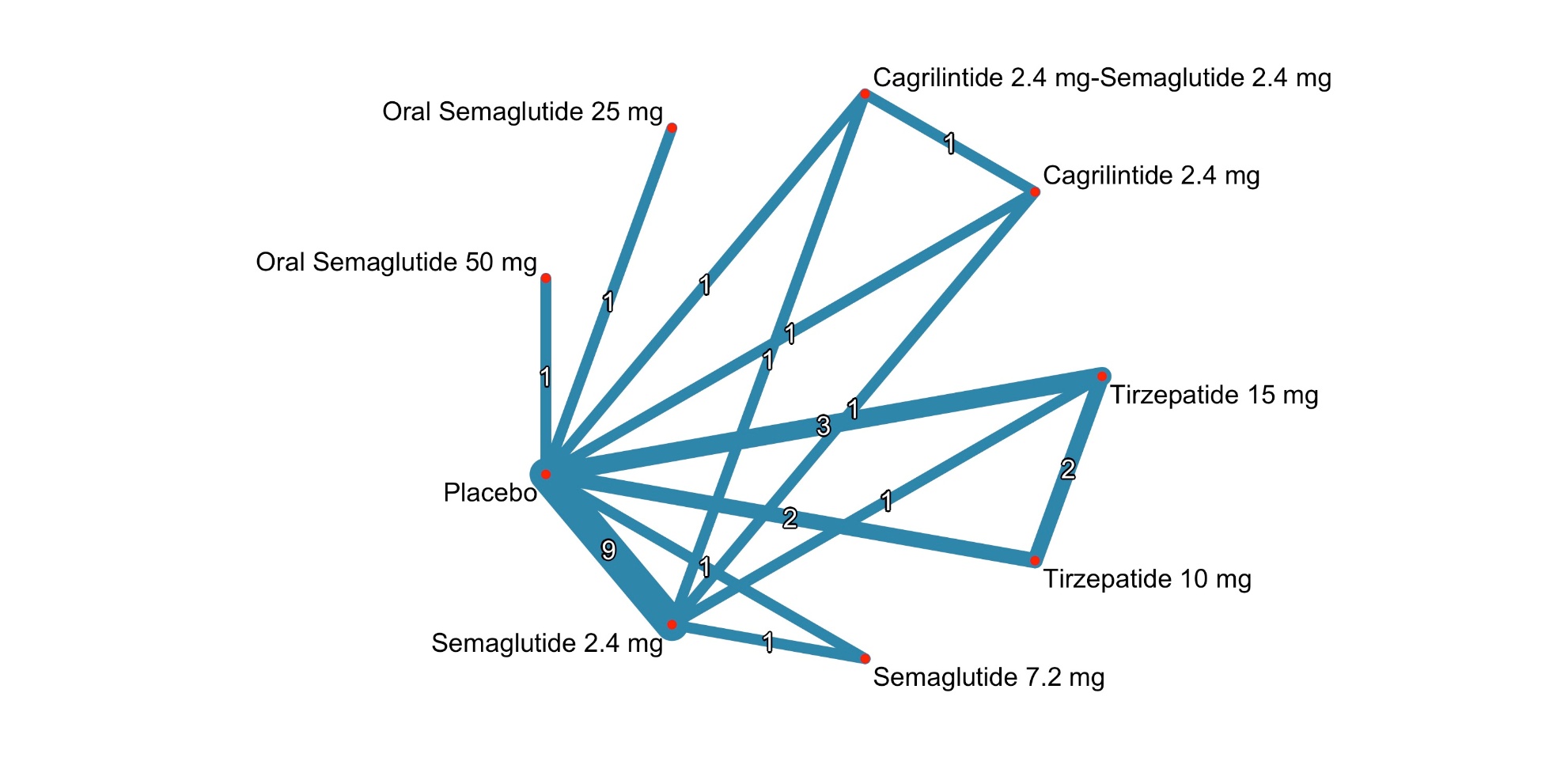


**Figure S2.9: Network Plot of Any Adverse Event**


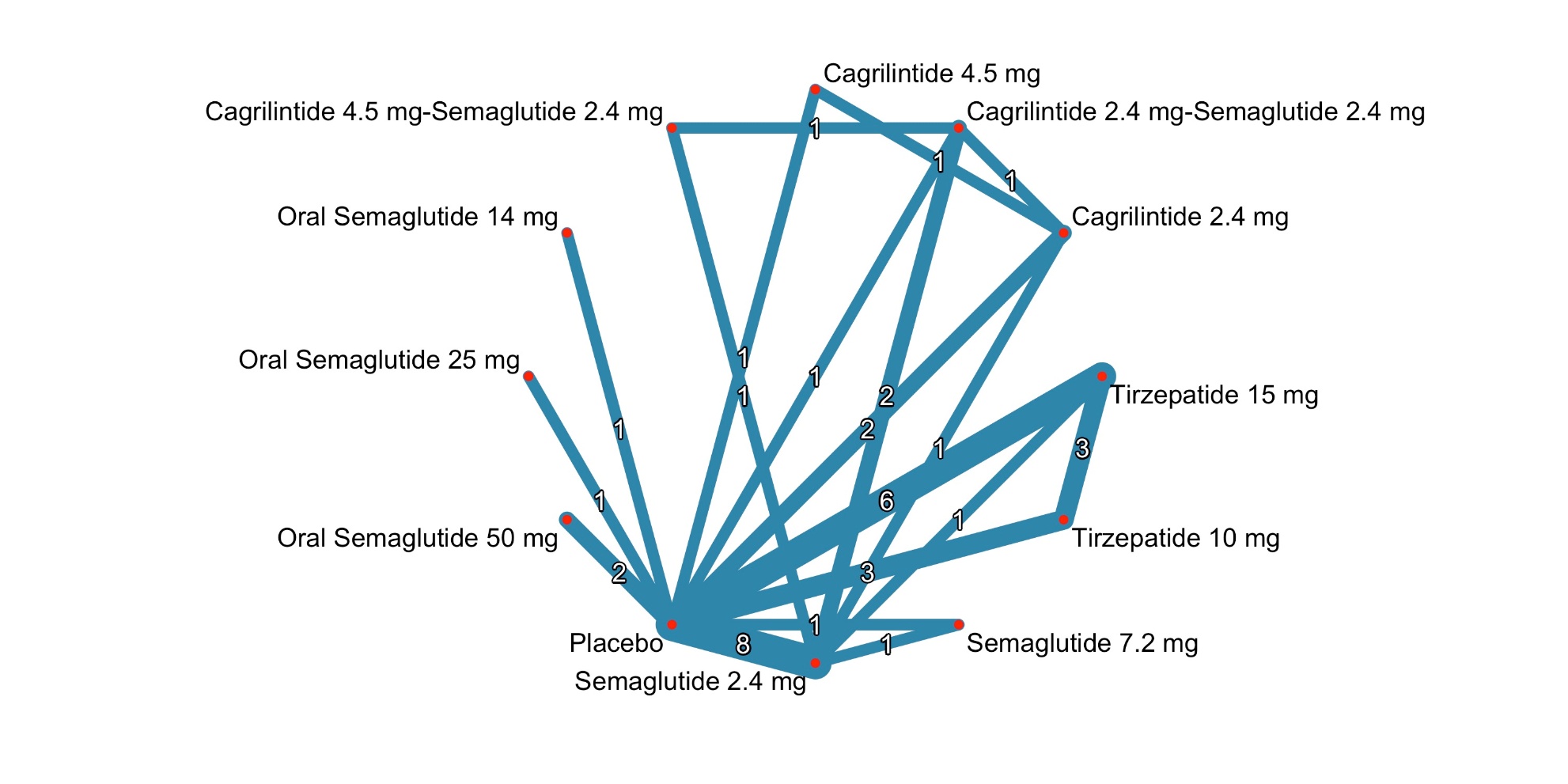


## **Figure S2.10: Network Plot of Serious Adverse Event**


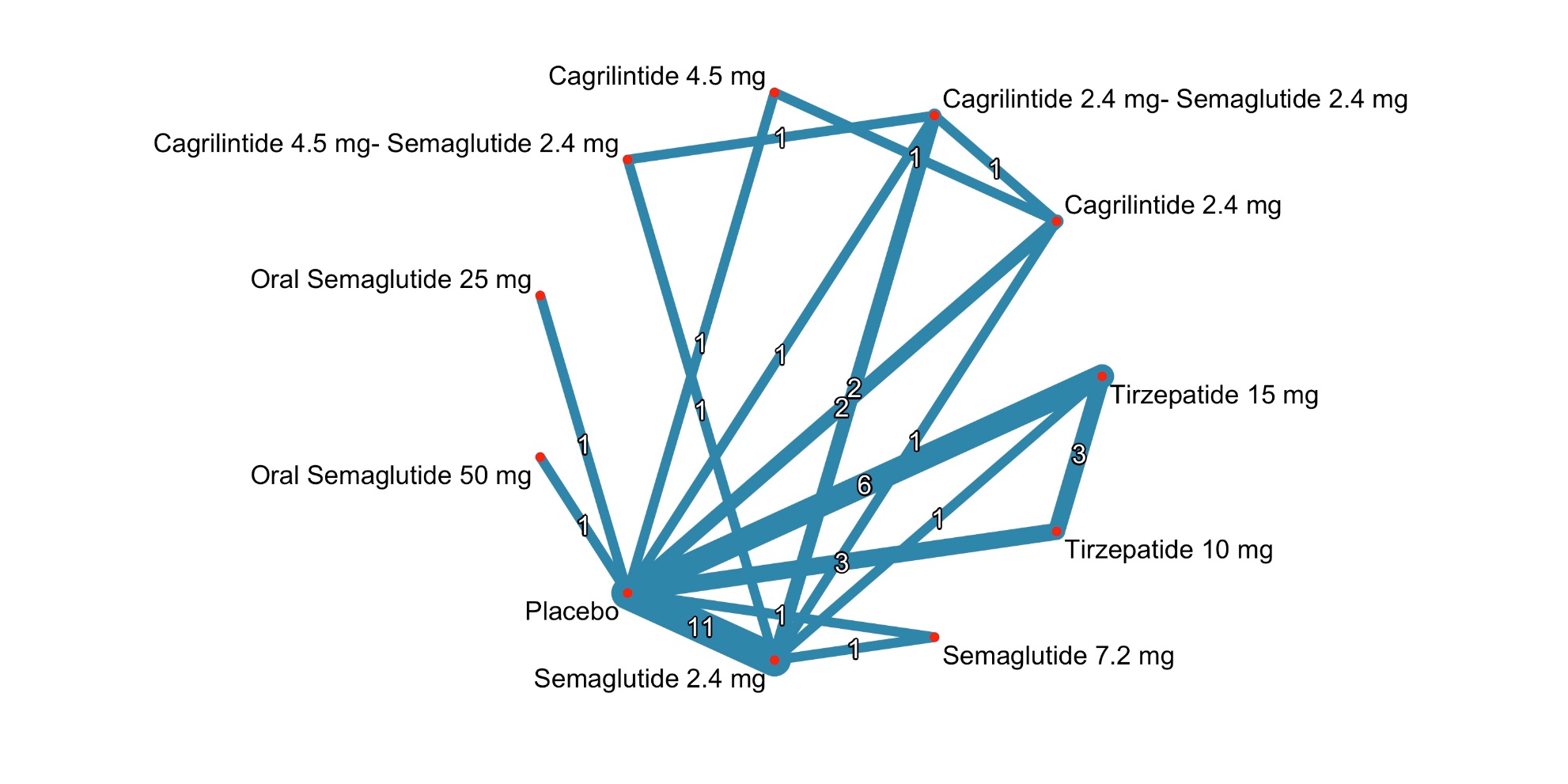


## **Figure S2.11: Network Plot of GI Adverse Events**


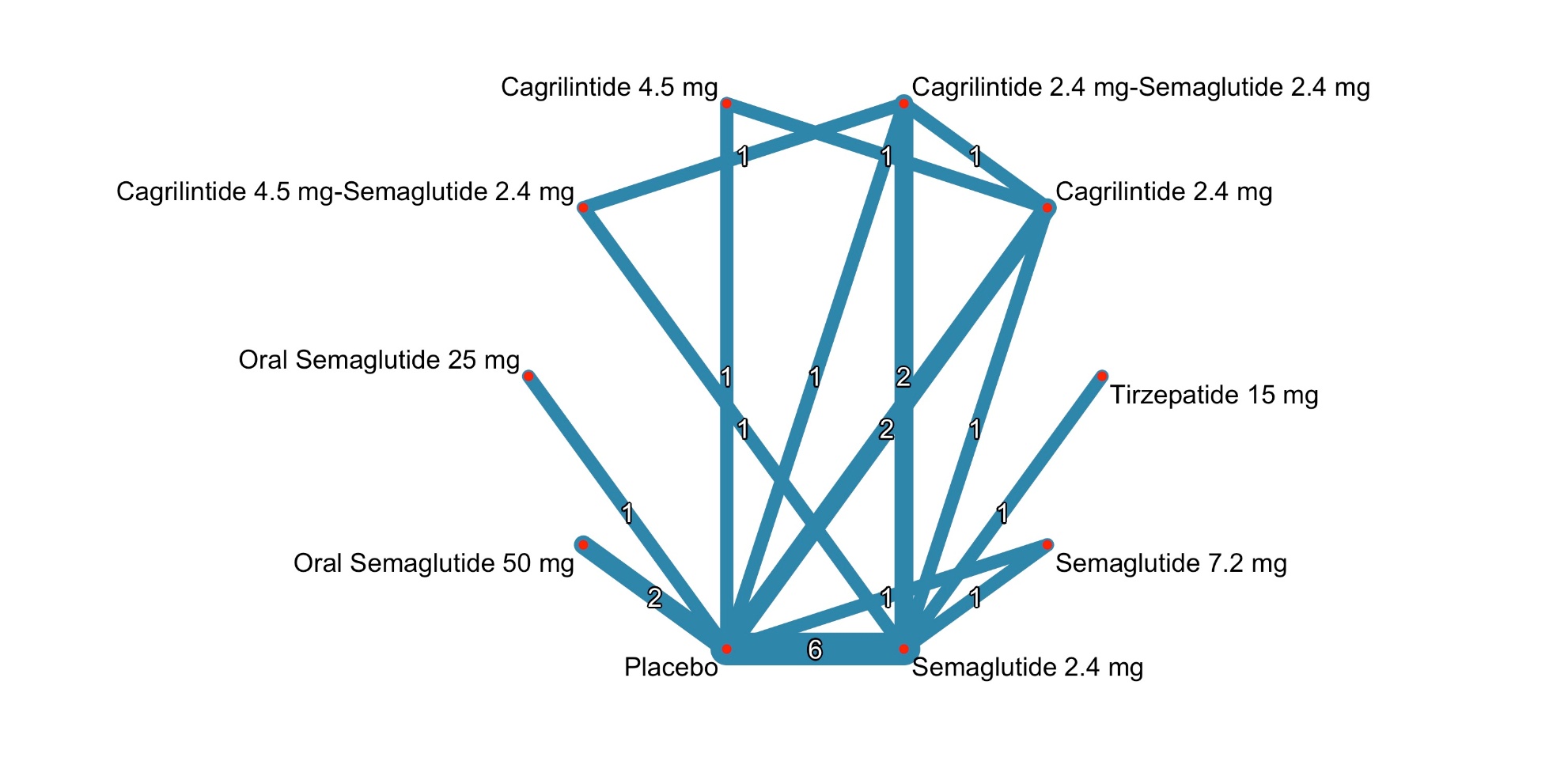


## **Figure S2.12: Network Plot of Adverse Events Leading to Drug Discontinuation**


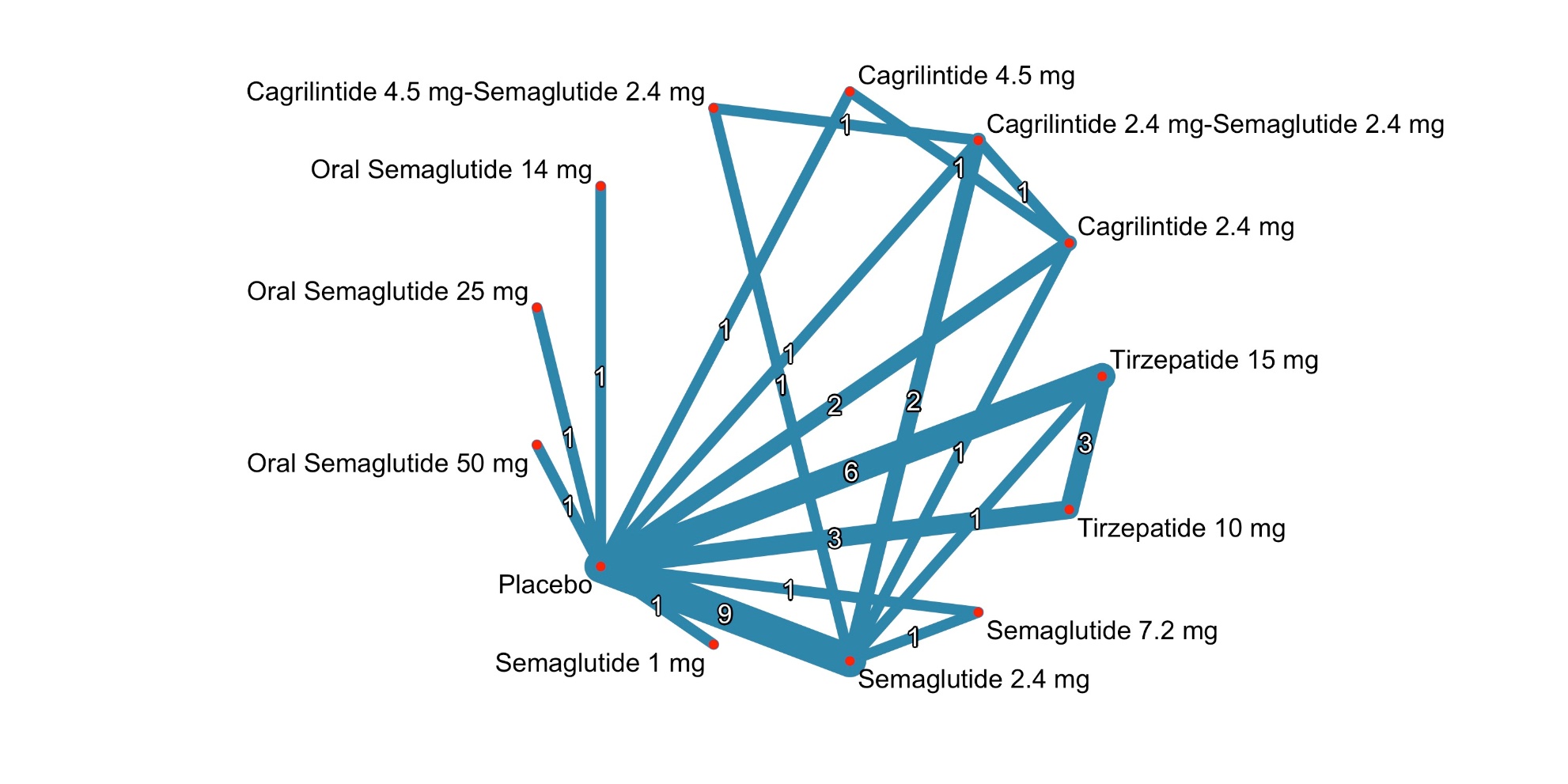


## **Figure S2.13: Network Plot of Change in HDL Cholesterol**


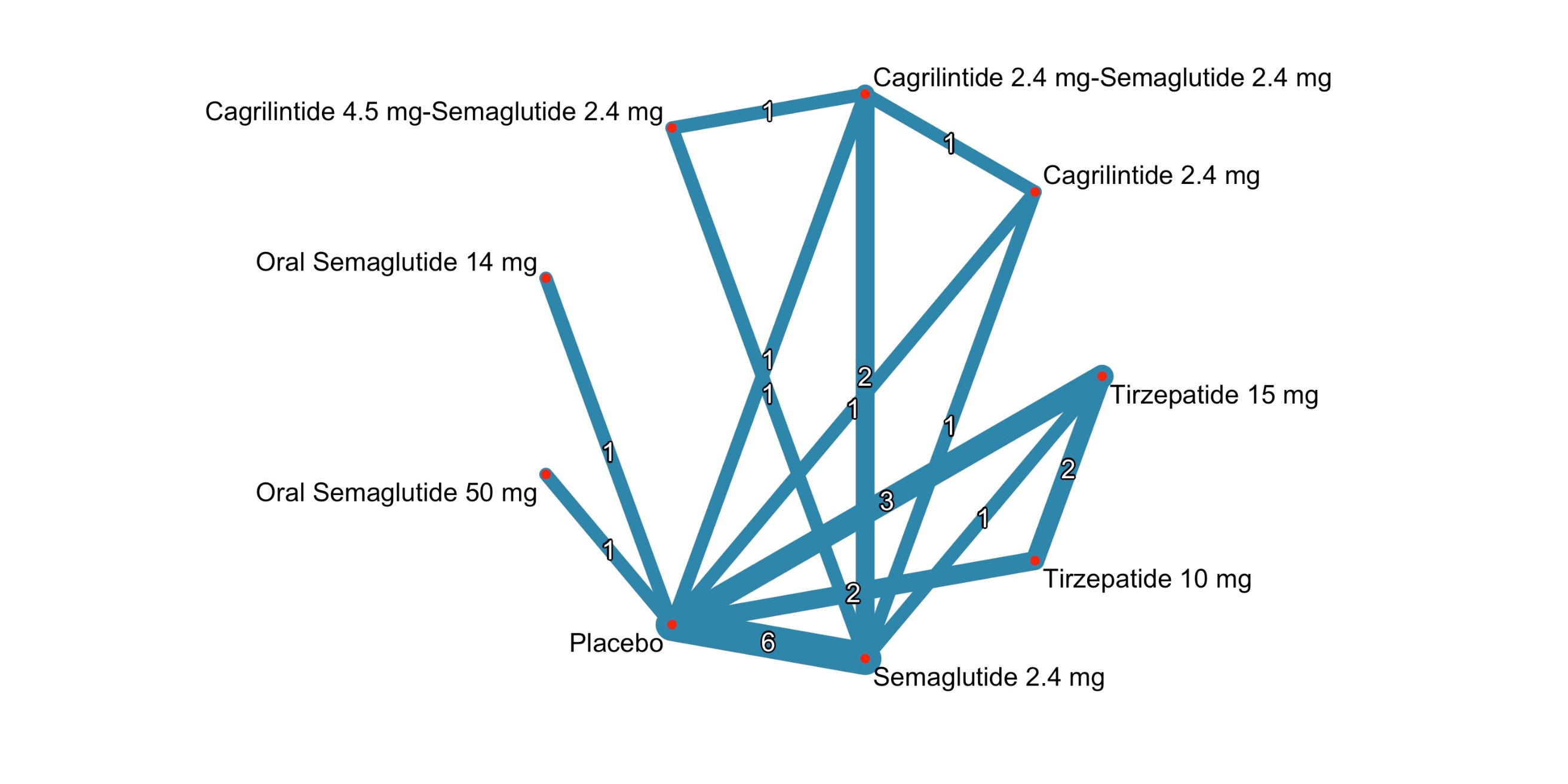


# **Supplement S3 Baseline characteristics of the included studies**

The table summarizes various clinical trials, detailing study design, registration, duration, treatment arms, and population characteristics.

| **Study ID** | **Trial Phase** | **Year** | **NCT Code** | **Population Included** | **Population Excluded** | **Number of Participants** | **Intervention** | **Comparator** | **Duration (weeks)** |
| --- | --- | --- | --- | --- | --- | --- | --- | --- | --- |
| Lau,2021 | Phase 3 | 2021 | NCT03856047 | Adults with BMI ≥30 kg/m² or BMI ≥ 27 kg/m² with comorbidity | DM 1 or 2, obesity treatment with surgery or a weight loss device. | 706 | Cagrilintide 0.3 mg  Cagrilintide 0.6 mg  Cagrilintide 1.2 mg  Cagrilintide 2.4 mg  Cagrilintide 4.5 mg | Placebo | 26   \|  \|  \| \| --- \| --- \| |
| Katrevula,2025 | Phase 4 | 2025 | NCT05442450 | Adults ≥18, BMI ≥ 30 kg/m² or ≥ 27 kg/m² with comorbidities | DM, History of chronic or acute pancreatitis, previous surgical obesity treatment, and recent use of anti-obesity medication within 90 days. | 116 | Oral Semaglutide 14 mg | Placebo | 28 |
| Knop,2023 | Phase 3 | 2023 | NCT05035095 | Adults ≥18, BMI ≥ 30 kg/m² or ≥ 27 kg/m² with body weight related comorbidity | Weight changes of more than 5 kg in the last 90 days, history of DM type 1 or 2 | 709 | Oral Semaglutide 50 mg | Placebo | 68 |
| Friedrichsen,2020 | Phase 1 | 2020 | NCT03842202 | Adults ≥18, BMI of 30 to 45 kg/m² | Change in body ≥ 5% last 90 days, presence of GI disorder or symptoms, use of weight-lowering drugs last 12 months | 72 | Semaglutide 2.4 mg | Placebo | 20 |
| Garvey,2025 | Phase 3 | 2025 | NCT05567796 | Adults ≥18, BMI ≥ 30 kg/m² or ≥ 27 kg/m² with at least one obesity comorbidity, except DM. | Prior surgical obesity treatment and use of GLP-1 RA or anti-obesity medication last 90 days | 3417 | Semaglutide 2.4 mg-Cagrilintide 2.4 mg. | Semaglutide 2.4 mg, Cagrilintide 2.4 mg, Placebo | 68 |
| Enebo,2021 | Phase 1 | 2021 | NCT03600480 | Healthy adults, aged 18–55 years, with a BMI of 27.0−39.9 kg/m² | HbA1c ≥ 6.5%, any weight loss ≥ 5%, adults ≥ 40 with risk of atherosclerotic cardiovascular disease | 95 | Cagrilintide 0.16 mg-Semaglutide 2.4 mg Cagrilintide 0.3 mg-Semaglutide 2.4 mg Cagrilintide 0.6 mg-Semaglutide 2.4 mg Cagrilintide 1.2 mg-Semaglutide 2.4 mg Cagrilintide 2.4 mg-Semaglutide 2.4 mg Cagrilintide 4.5 mg-Semaglutide 2.4 mg | Placebo-semaglutide 2.4 mg | 20 |
| Rubino,2022 | Phase 3 | 2022 | NCT04074161 | Adults with BMI ≥ 30 or ≥ 27 kg/m² with comorbidity and failed dietary attempts for weight loss | Diabetes, body weight change of more than 5 kg last 90 days | 338 | Semaglutide 2.4 mg | Placebo | 68 |
| Blundell,2017 | Phase 1 | 2017 | NCT02079870 | Adults ≥18, with BMI 30-45 kg/m², HbA1c <6.5% | T1DM or T2DM | 58 | Semaglutide 1.0 mg | Placebo | 28 |
| Aronne,2025 | Phase 3 | 2025 | NCT05822830 | Adults with BMI ≥ 30 or ≥ 27 kg/m² with obesity-related comorbidity, except T2DM | Treatment with weight-lowering medication or GLP-1A last 90 days and surgical treatment for obesity | 750 | Tirzepatide 10 mg or 15 mg (MTD) | Semaglutide 1.7mg or 2.4mg (MTD) | 72 |
| Gabe,2024 | Phase 1 | 2024 | NCT05236517 | Healthy adults with a BMI between 30-45 kg/m² | Patients with clinically relevant metabolic, renal, endocrine and GI disorders | 61 | Oral Semaglutide 50 mg | Placebo | 20 |
| McGowan, 2024 | Phase 3 | 2024 | NCT05040971 | Adults ≥ 18, BMI ≥ 30 kg/m² with prediabetes | History of T1DM or T2DM | 207 | Semaglutide 2.4mg | Placebo | 80 |
| Zhao,2024 | Phase 3 | 2024 | NCT05024032 | Chinese adults ≥ 18, BMI ≥ 28 kg/m² or 24 kg/m² with comorbidity | T1DM or T2DM, self-reported weight loss of 5 kg or more last 3 months | 210 | Tirzepatide 10 mg, Tirzepatide 15 mg | Placebo | 52 |
| Wharton,2025_b | Phase 3 | 2025 | NCT05646706 | Adults ≥ 18, BMI ≥ 30 kg/m² with failed dietary effort to lose bodyweight | History of DM, self-reported change in body weight >5kg in the last 90 days, HbA1c ≥ 6.5 | 1407 | Semaglutide 7.2mg, Semaglutide 2.4mg | Placebo | 72 |
| Kadowaki,2025 | Phase 3 | 2025 | NCT04844918 | BMI between 27 kg/m² and 35 kg/m²with at least two obesity complications or BMI > 35 kg/m² and at least one obesity comorbidity | History of DM, use of weight-lowering medications and liver disease other than MASLD | 225 | Tirzepatide 10 mg, Tirzepatide 15 mg | Placebo | 72 |
| Wharton,2025_a | Phase 3 | 2025 | NCT05564117 | Adults with BMI ≥ 30 kg/m² or ≥ 27 kg/m² with weight related comorbidity and failed weight loss attempt | History of T1DM or T2DM | 307 | Oral Semaglutide 25 mg | placebo | 64 |
| Lim,2025 | Phase 3 | 2025 | NCT04998136 | Adults with BMI ≥25 kg/m² with at least one unsuccessful weight-loss attempt | History of DM, change in bodyweight of more than 5 kg, or use weight weight-lowering medications last 3 months | 150 | Semaglutide 2.4 mg | Placebo | 44 |
| Bliddal,2024, | Phase 3 | 2024 | NCT05064735 | Adults ≥ 18 with a diagnosis of osteoarthritis, BMI ≥ 30 kg/m² | Knee replacement surgery, history of DM, and use of weight-lowering medications in the last 90 days | 407 | Semaglutide 2.4 mg | Placebo | 68 |
| O’Neil,2018 | Phase 2 | 2018 | NCT02453711 | Adults (≥18 years) with BMI>30 and undergone at least one failed weight-lowering non-surgical intervention | History of DM, a change in weight of 5 kg or more last 90 days, and a secondary endocrine cause for obesity | 957 | Semaglutide 0.05 mg  Semaglutide 0.1 mg  Semaglutide 0.2 mg  Semaglutide 0.3 mg  Semaglutide 0.4 mg | Placebo | 52 |
| Wadden,2023 | Phase 3 | 2023 | NCT04657016 | Adults with BMI ≥ 30 kg/m² or ≥ 27 kg/m² with weight related comorbidity | History of T1DM or T2DM | 579 | Tirzepatide 10 or 15 mg (MTD) | placebo | 72 |
| Malhotra,2024_Trial 1 | Phase 3 | 2024 | NCT05412004 | Moderate to severe OSA, with BMI ≥30 kg/m² or ≥27 kg/m² in Japan | History of T1DM or T2DM, planned surgery for OSA, and a change in weight > 5 kg in the last 90 days | 234 | Tirzepatide 10 mg or 15 mg (MTD) | Placebo | 52 |
| Malhotra,2024_Trial 2 | Phase 3 | 2024 | NCT05412004 | Moderate to severe OSA, with BMI ≥30 kg/m² or ≥27 kg/m² in Japan | History of T1DM or T2DM, planned surgery for OSA, and a change in weight > 5 kg in the last 90 days | 235 | Tirzepatide 10 mg or 15 mg (MTD) | Placebo | 52 |
| Jastreboff,2022 | Phase 3 | 2022 | NCT04184622 | Adults with BMI ≥ 30 kg/m² or ≥ 27 kg/m² with weight related comorbidity | History of DM, weight-lowering medication last 90 days, and change in weight >5 kg last 3 months | 2539 | Tirzepatide 5 mg,  Tirzepatide 10 mg  Tirzepatide 15 mg | Placebo | 72 |
| Garvey,2022 | Phase 3 | 2022 | NCT03693430 | Adults with BMI ≥ 30 kg/m² or ≥ 27 kg/m² with weight-related comorbidity and a history of one or more failed dietary attempts. | History of DM, planned bariatric surgery, and uncontrolled endocrine disorder | 304 | Semaglutide 2.4 mg | Placebo | 104 |
| Lincoff,2023 | Phase 3 | 2023 | NCT03574597 | Adults ≥ 45 with BMI ≥27 kg/m² and had establishe CVD disease | History of DM, HF with NYHA class IV heart failure, and end-stage kidney disease or dialysis | 17604 | Semaglutide 2.4 mg | Placebo | 104 |
| Wilding,2021 | Phase 3 | 2021 | NCT03548935 | Adults ≥ 18 with BMI ≥ 30 kg/m² or ≥ 27 kg/m² with weight-related comorbidity. | T1DM or T2DM, history of acute or chronic pancreatitis last 6 months, and use of weight-lowering drugs last 90 days | 1961 | Semaglutide 2.4 mg | Placebo | 68 |
| Waddan,2021 | Phase 3 | 2021 | NCT03611582 | Adults ≥ 18 with BMI ≥ 30 kg/m² or ≥ 27 kg/m² with weight-related comorbidity and unsuccessful dietary weight losing attempt or more | History of DM, planned obesity surgery, and use of anti-obesity medications last 90 days | 611 | Semaglutide 2.4 mg | Placebo | 68 |

**Abbreviations**:

**OSA:** Obstructive sleep apnea

**BMI:** Body Mass Index

**DM:** Diabetes Miletus

**HF**: Heart Failure

**GI:** Gastrointestinal

**NYHA:** New York Heart Association

**CVD**: Cardiovascular Disease

**GLP-1 RA:** Glucagon-like peptide-1 receptor agonists

**MASLD:** Metabolic Dysfunction-Associated Steatosis Liver Disease

**MTD:** Maximum Tolerated Dose

# **Supplement S4. Baseline characteristics of the participants**

The table summarizes the baseline characteristics of patients included in the study. Characteristics include Age, Male, Body Weight, BMI, Waist Circumference, SBP, DBP, HbA1c, waist TG, and LDL.

| **Study ID** | **Age**  **(mean, SD)** | **Male (%)** | **BMI (kg/m²)** | **Body Weight (kg)** | **Waist Circumference (cm)** | **SBP** | **DBP** | **HbA1c (%)** | **TGs (mg/Dl)** | **LDL cholesterol (mg/Dl)** |
| --- | --- | --- | --- | --- | --- | --- | --- | --- | --- | --- |
| Lau,2021 | 52.3 ± 10.6 | 37.8 | 37.8 ± 7 | 107.4 ± 24.2 | 115.4 ± 15.4 | 132 ± 14.6 | 82.7 ± 9.6 | 5.6 ± 0.4 | NR | NR |
| Katrevula,2025 | 44±9 | 36.2 | 33.2±3.7 | 91.1±14.9 | 109.7±8.9 | 131±12 | 87±10 | 5.8±0.4 | 150.4 | 115.5 |
| Knop,2023 | 50±13 | 27 | 37.5±6.5 | 105.4±22.2 | 113.6±14.6 | 129±15 | 82±11 | 5.6±0.3 | 124 | 112.14 ±32.7 |
| Friedrichsen,2020 | 42.8±11.1 | 61.1 | 34.4±3 | 105.5±15 |  |  |  |  |  |  |
| Garvey,2025 | 47±11.8 | 32.4 | 37.9±6.7 | 106.9±23.1 | 114.7±15.5 | 127.1±14.2 | 82.1±9.2 | 5.5±0.4 | 121.2 | 138.8 |
| Enebo,2021 | 40.6±9.2 | 59 | 32.1±3.4 | 95.7±13.6 |  |  |  | 5.3±0.4 | 88.57±65.54 | 116.01 ±23.2 |
| Blundell,2017 | 42 | 66.7 | 33.8 | 101.3 |  |  |  |  |  |  |
| Aronne,2025 | 44.7±12.8 | 35.3 | 39.4±7.6 | 113±25.6 | 118.3±16.9 | 125.7±13.02 | 81.3±8.26 | 5.6±0.36 | 130.3±88 | 114.1±31.2 |
| Gabe,2024 | 44±11 | 65.6 | 34.9±3.8 | 106.1±16 |  |  |  | 5.3±0.4 |  |  |
| McGowan,2024 | 53±11 | 29 | 40.1±6.9 | 111.6±22.2 | 120.1±14.7 | 131±15 |  | 5.9±0.3 | 88.57 | 77.34 |
| Wharton,2025_b | 47±12 | 26.3 | 39.9±7.1 | 113±24.1 | 118.7±15.8 |  |  | 5.7±0.3 |  |  |
| Kadowaki,2025 | 50.8±10.7 | 59 | 33.5±4.4 | 92±14.9 | 108±10.4 | 125.4±12.6 | 79.8±9 | 5.66±0.33 | 186±90.1 |  |
| Lim,2025 | 39±11 | 26 | 31.3±5.2 | 83.8±18.1 | 98.1±12.3 | 127±13 | 78±10 | 5.6±0.3 | 132.85 | 112.14 |
| O’Neil,2018 | 47±12 | 35 | 93.3±7 | 111.15±23.4 | 117.8±14.9 |  |  | 5.5±0.4 | 132.86 | 116.01 |
| Bliddal,2024 | 56±10 | 18.4 | 40.3±7.2 | 108.6±24.2 | 118.7±15.8 | 132±15 | 82±10 |  |  |  |
| Wadden,2023 | 45.6±12.2 | 37.1 | 35.9±6.3 | 101.9±21.4 | 109.4±15 | 121±12.6 | 78.6±9.1 | 5.4±0.4 | 120±54.5 | 112.4±32.4 |
| Jastreboff,2022 | 44.9±12.5 | 32.5 | 38±6.81 | 104.8±22.12 | 114.1±15.16 | 123.3±12.73 | 79.5±8.16 | 5.6±0.38 | 128.4 | 109.5 |
| Zhao,2024 | 36.09±9.03 | 50.95 | 32.33±3.8 | 91.83±16 | 104.83±10.35 | 119.99±12.31 | 82.33±8.66 | 5.61±0.32 | 151.85 | 110.11 |
| Wharton,2025_a | 47.6±12.99 | 21.17 | 37.6±6.5 | 105.86±22.92 | 113.87±15.42 | 131.2±16.66 | 83.07±9.98 | 5.7±0.37 |  |  |
| Garvey,2022 | 47.35±11 | 22.4 | 38.55±6.94 | 106.05±21.95 | 115.75±14.98 | 125.5±14.49 | 80±9.5 | 5.7±0.35 | 110.5 | 112.14 |
| Lincoff,2023 | 61.6±8.85 | 72.34 | 33.35±5 | 96.65±17.65 | 111.35±13.1 | 130.95±15.45 | 79.3±15.15 | 5.78±0.34 | 141±66.36 | 80.33±30.4 |
| Wilding,2021 | 46.33±12.68 | 25.93 | 37.87±6.63 | 105.33±21.9 | 114.67±14.66 | 126.33±14 | 79.33±16.52 | 5.7±0.3 | 126.77 | 111.03 |
| Waddan,2021 | 46±13 | 20.03 | 38±6.76 | 105.83±22.86 | 113±15.94 | 124±15 | 80.33±10 | 5.75±0.3 | 108.9±48.55 | 109.07±3.64 |
| Malhotra,2024_Trial_1 | 47.9 ±11.5 | 67.1 | 39.1±7 | 114.7±23.7 | 121.2±15.7 | 129.4± 11.5 | 83.8±8.7 | 5.67 ± 0.36 |  |  |
| Malhotra,2024_Trial_2 | 51.7 ± 11 | 72.3 | 38.7± 6 | 115.5 ± 22 | 120.9 ± 13.5 | 130.5± 13.5 | 81.8 ± 8.5 | 5.63 ± 0.41 |  |  |
| Rubino,2022 | 49.21± 13.28 | 20.73 | 37.3± 7.09 | 105.04 ± 24.58 | 113.25±15.89 | 124.19± 14 | 80.19 ± 9.03 | 5.54 ± 0.35 | 109.33 ±49 | 105.9± 32.9 |

## **Abbreviations**:

**BMI:** Body Mass Index **HbA1c:** Hemoglobin A1c

**SBP:** Systolic Blood Pressure **LDL:** Low density lipoprotein

**DPB:** Diastolic Blood Pressure **TG:** Triglyceride

# **Supplement S5. Risk of bias table**

| **Study ID** | **Random Sequence Generation** | **Allocation Concealment** | **Blinding of Participants/Personnel** | **Blinding of Outcome Assessment** | **Incomplete Outcome Data** | **Selective Reporting** | **Other Bias** | **Overall Judgment** |
| --- | --- | --- | --- | --- | --- | --- | --- | --- |
| Lau,2021 | Low Risk | Low Risk | Low Risk | Low Risk | Low Risk | Low Risk | No | Low Risk |
| Katrevula,2025 | Low Risk | High Risk | High Risk | High Risk | Low Risk | Low Risk | No | High Risk |
| Friedrichsen,2020 | Some Concern | Some Concern | Low Risk | Low Risk | Low Risk | Low Risk | Funded by Novo Nordisk | Some Concern |
| Knop,2023 | Low Risk | Low Risk | Low Risk | Low Risk | Low Risk | Low Risk | Funded by Novo Nordisk | Low Risk |
| Garvey,2025 | Low Risk | Low Risk | Low Risk | Low Risk | Low Risk | Low Risk | Funded by Novo Nordisk | Low Risk |
| Enebo,2021 | Low Risk | Low Risk | Low Risk | Low Risk | Low Risk | Low Risk | Funded by Novo Nordisk | Low Risk |
| Rubino,2022 | Low Risk | Low Risk | Low Risk | Low Risk | Low Risk | Low Risk | Funded by Novo Nordisk, randomization to semaglutide or liraglutide was not masked | Low Risk |
| Blundell,2017 | Some Concern | Low Risk | Low Risk | Some Concern | Some Concern | Low Risk | Funded by Novo Nordisk | Some Concern |
| Aronne,2025 | Low Risk | High Risk | High Risk | High Risk | Low Risk | Low Risk | Funded by Eli Lilly | High Risk |
| McGowan, 2024 | Low Risk | Low Risk | Low Risk | Low Risk | Low Risk | Low Risk | Funded by Novo Nordisk | Low Risk |
| Gabe,2024 | Low Risk | Low Risk | Low Risk | Some Concern | Some Concern | Low Risk | Funded by Novo Nordisk | Some Concern |
| Zhao.2024 | Low Risk | Low Risk | Low Risk | Low Risk | Low Risk | Low Risk | Funded by Eli Lilly | Low Risk |
| Wharton,2025b | Low Risk | Low Risk | Low Risk | Low Risk | Low Risk | Low Risk | Funded by Novo Nordisk | Low Risk |
| Kadowaki,2025 | Low Risk | Low Risk | Low Risk | Low Risk | Low Risk | Low Risk | Funded by Eli Lilly | Low Risk |
| Lim.2025 | Low Risk | Low Risk | Low Risk | Low Risk | Low Risk | Low Risk | Funding Novo Nordisk. | Low Risk |
| O’Neil,2025 | Low Risk | Low Risk | Low Risk | Low Risk | Low Risk | Low Risk | The funder of the study had a role in the study design and management, data collection, data interpretation, and data analysis. (funded by Novo Nordisk) | Low Risk |
| Wadden,2023 | Low Risk | Low Risk | Low Risk | Low Risk | Some Concern | Low Risk | Funded by Eli Lilly, >20% dropped out | Some Concern |
| Wharton,2025 | Low Risk | Low Risk | Low Risk | Low Risk | Low Risk | Low Risk | Funded by Novo Nordisk | Low Risk |
| Bliddal,2024 | Low Risk | Low Risk | Low Risk | Low Risk | Low Risk | Low Risk | Funded by Novo Nordisk | Low Risk |
| Malhotra,2024 | Low Risk | Low Risk | Low Risk | Low Risk | Low Risk | Low Risk | Funded by Eli Lilly | Low Risk |
| Jastreboff,2022 | Low Risk | Low Risk | Low Risk | Low Risk | Low Risk | Low Risk | Supported by Eli Lily | Low Risk |
| Garvey,2022 | Low Risk | Low Risk | Low Risk | Low Risk | Low Risk | Low Risk | Funded by Novo Nordisk | Low Risk |
| Lincoff,2023 | Low Risk | Low Risk | Low Risk | Low Risk | Low Risk | Low Risk | Funded by Novo Nordisk | Low Risk |
| Wilding,2021 | Low Risk | Low Risk | Low Risk | Low Risk | Low Risk | Low Risk | Funded by Novo Nordisk | Low Risk |


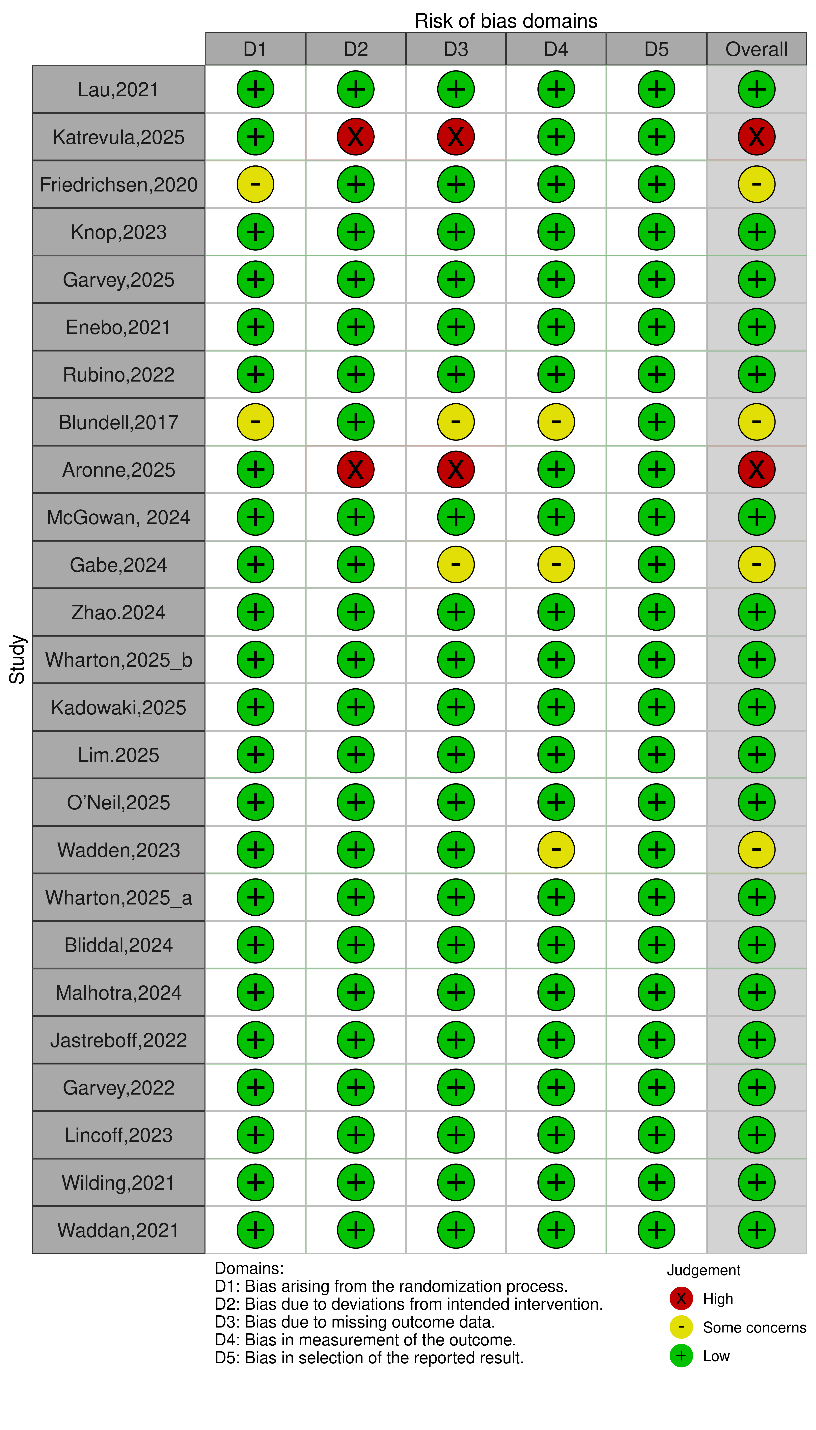


# **Supplement S6. Publication bias (funnel plot)**

## Figure S6.1: Percent Change in Body Weight


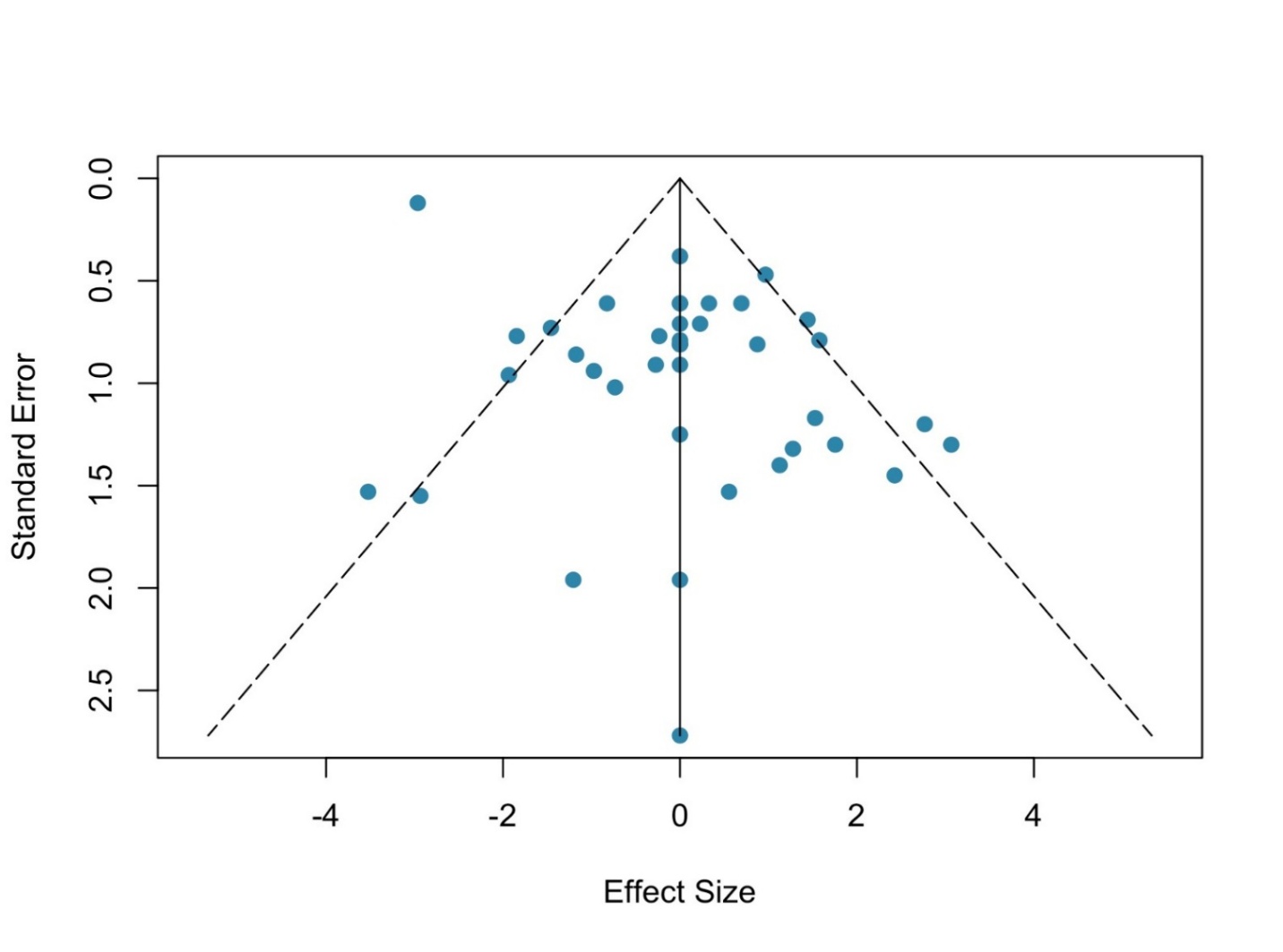


## Figure S6.2: Change in Waist Circumference


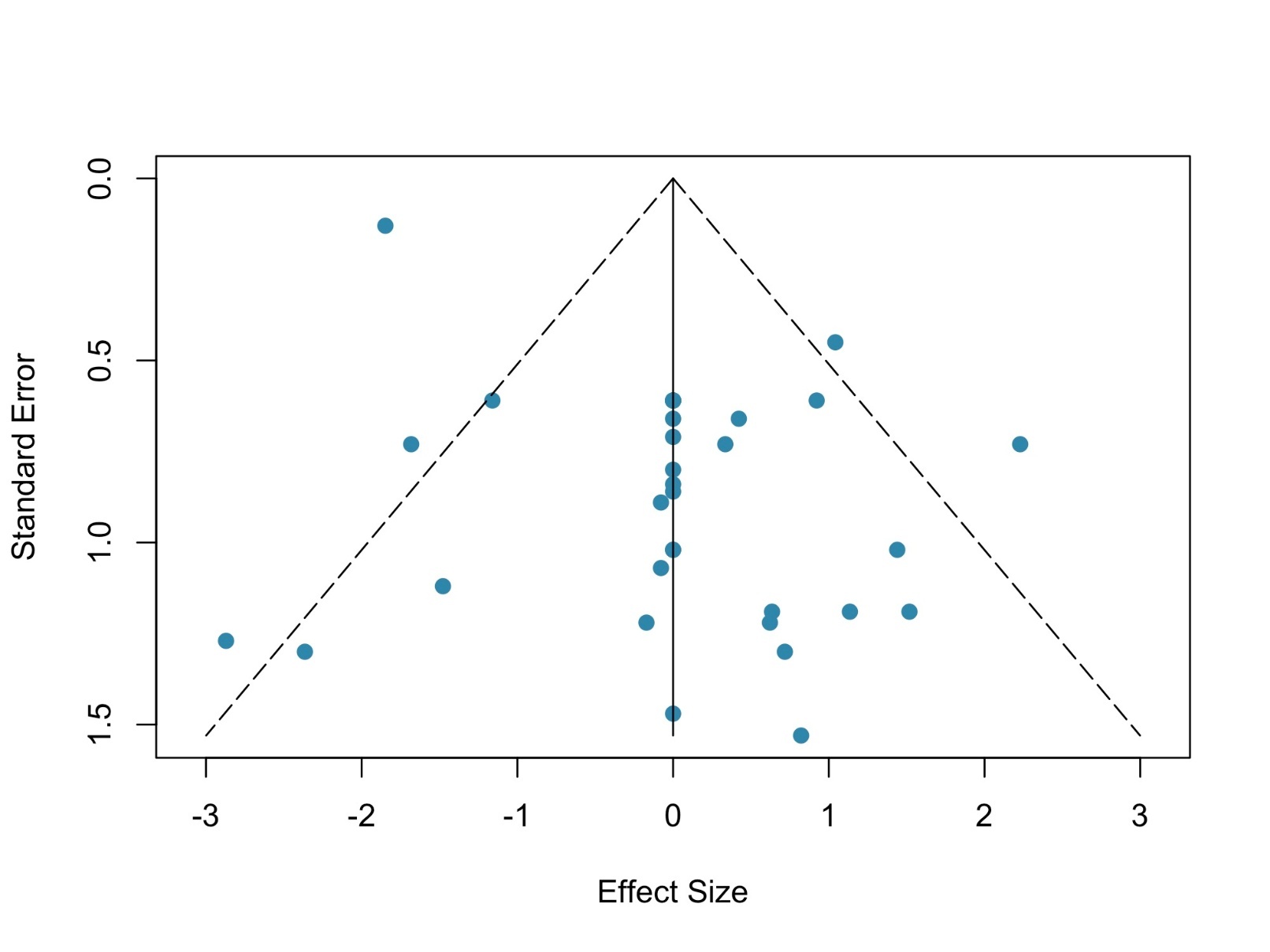


## Figure S6.3: Body Mass Index (BMI)


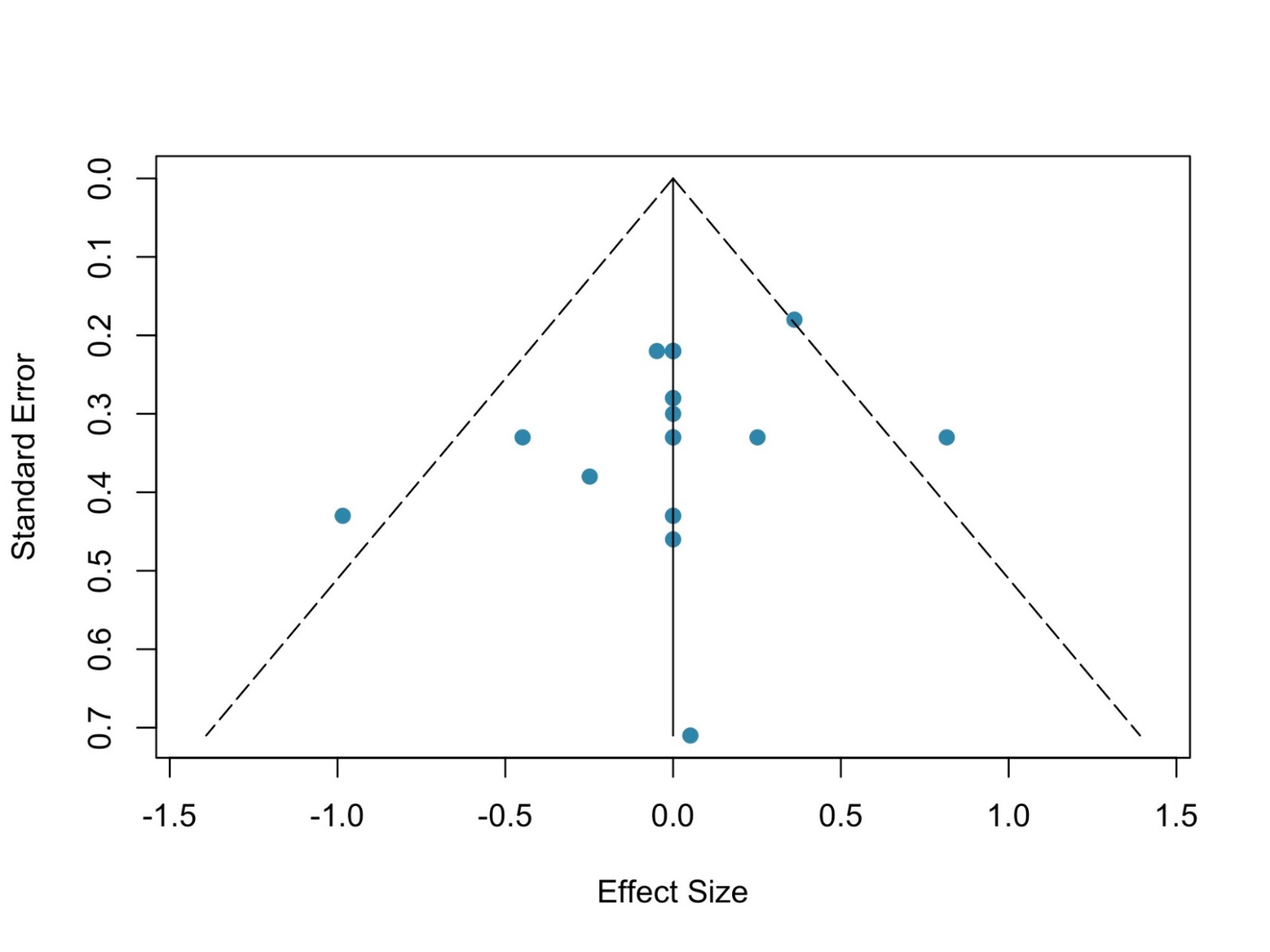


## Figure S6.4: Change in Body Weight


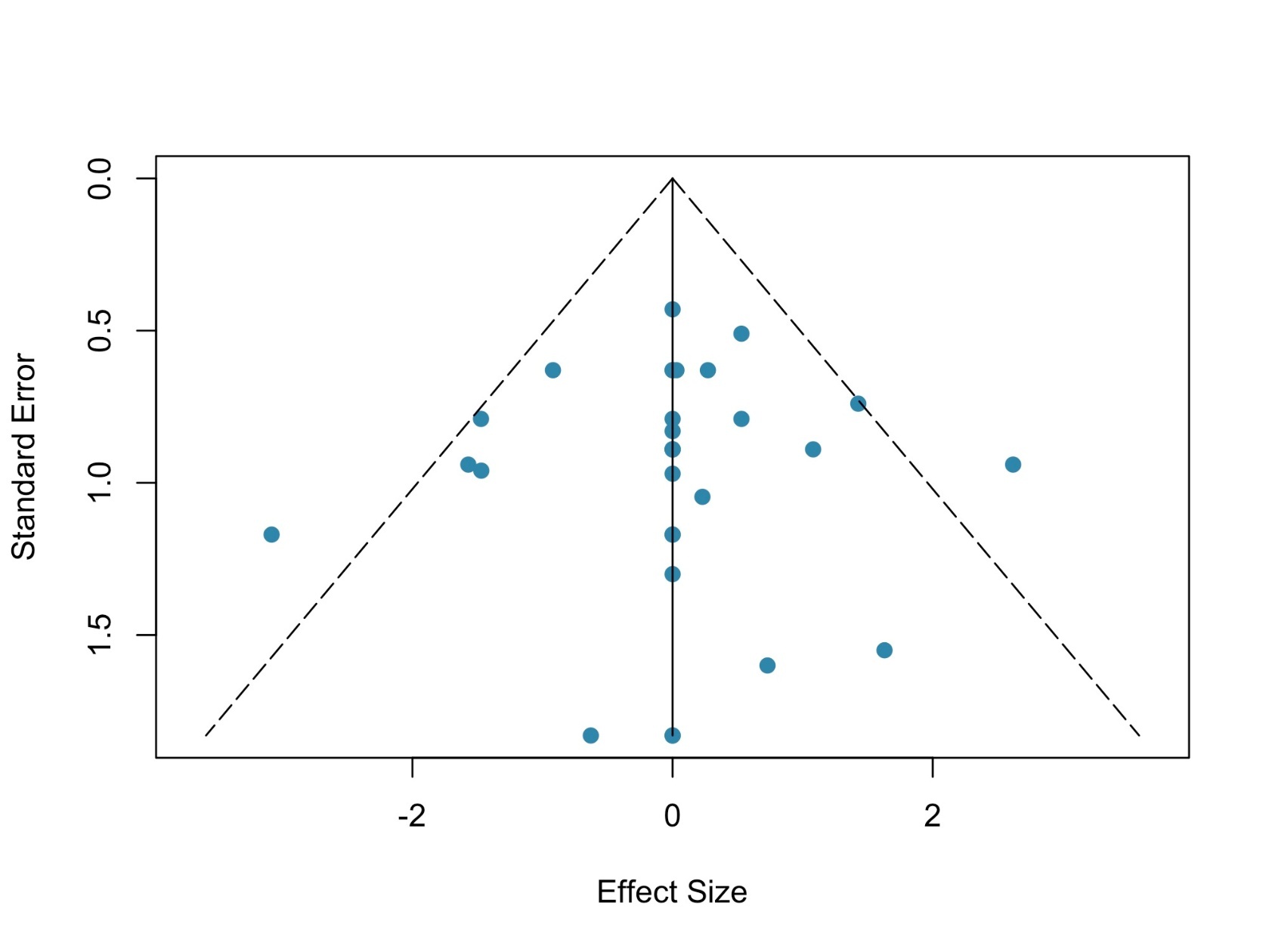


Figure S6.5: Achieving ≥ 5% weight loss


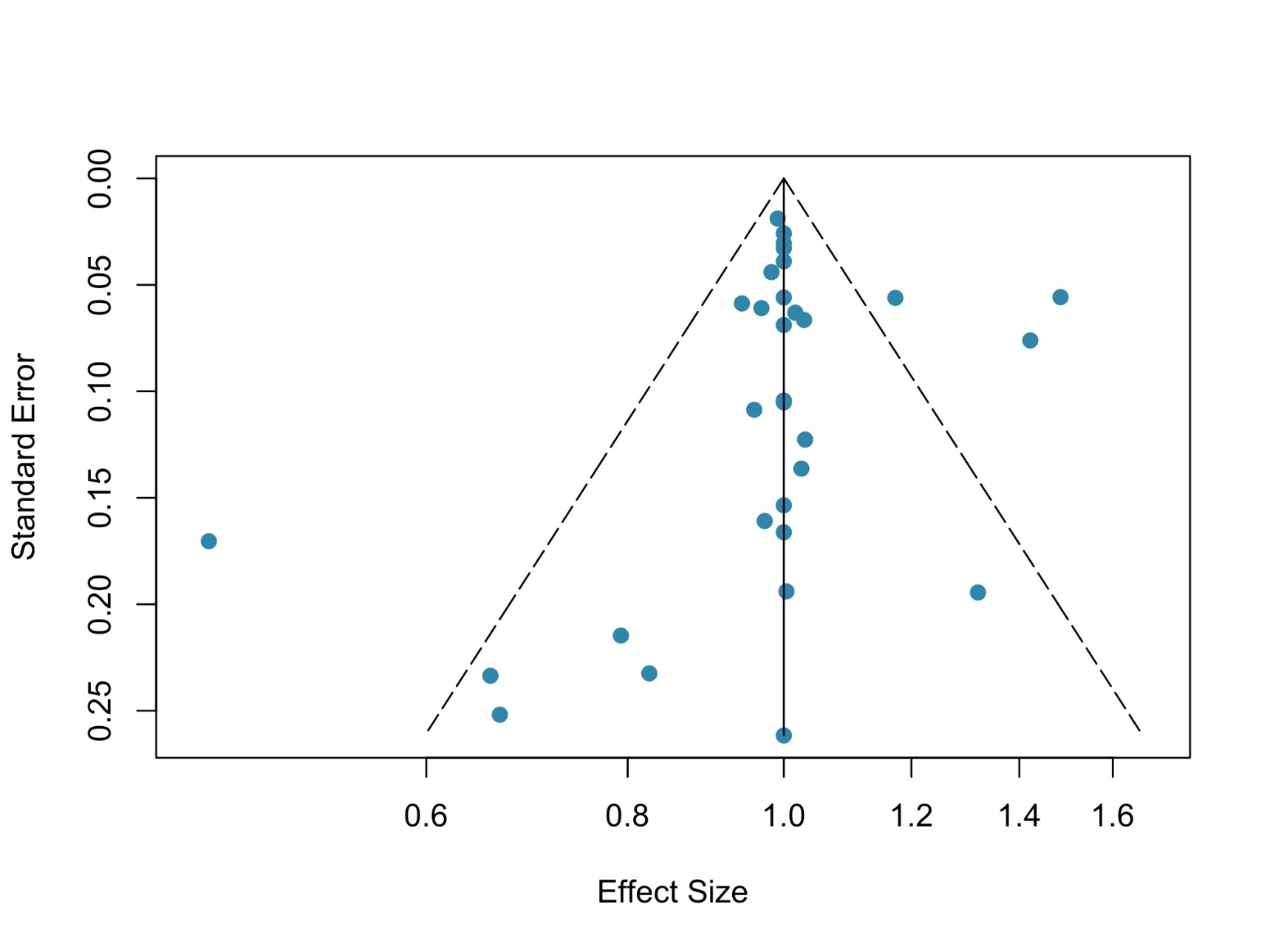


Figure S6.6: Achieving ≥ 10% weight loss


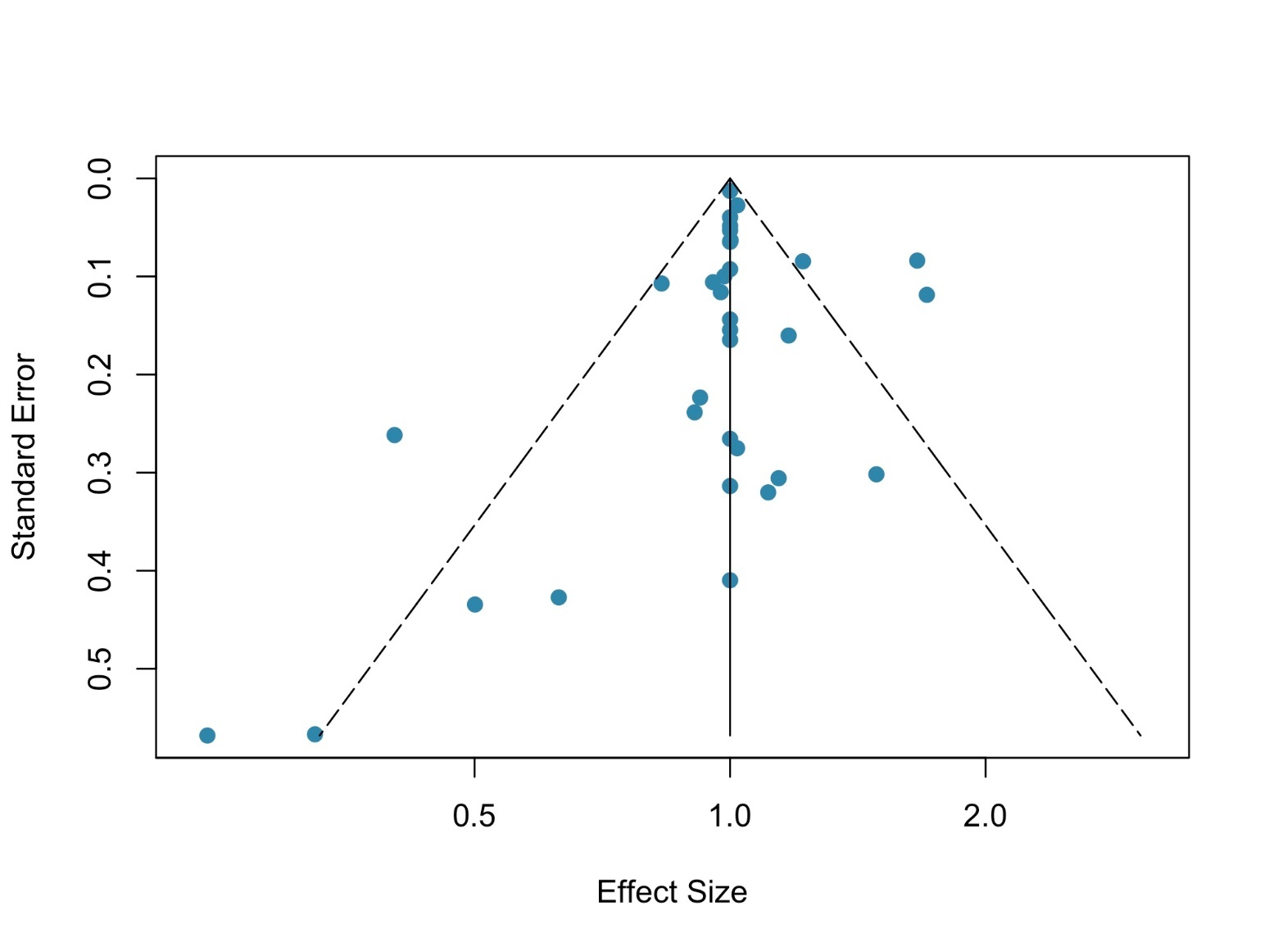


Figure S6.7: Achieving ≥ 15% weight loss


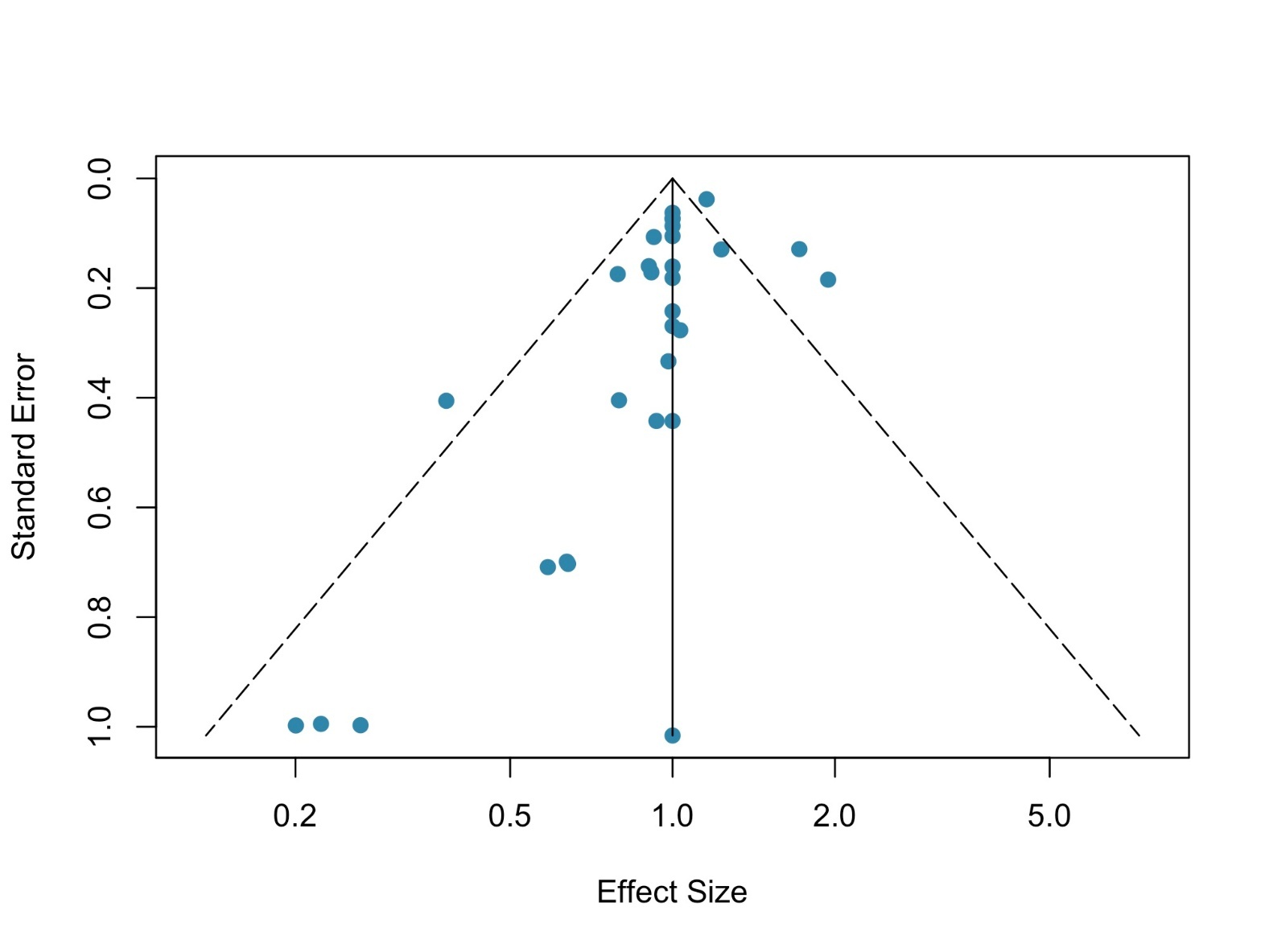


## Figure S6.8: Achieving ≥ 20% weight loss


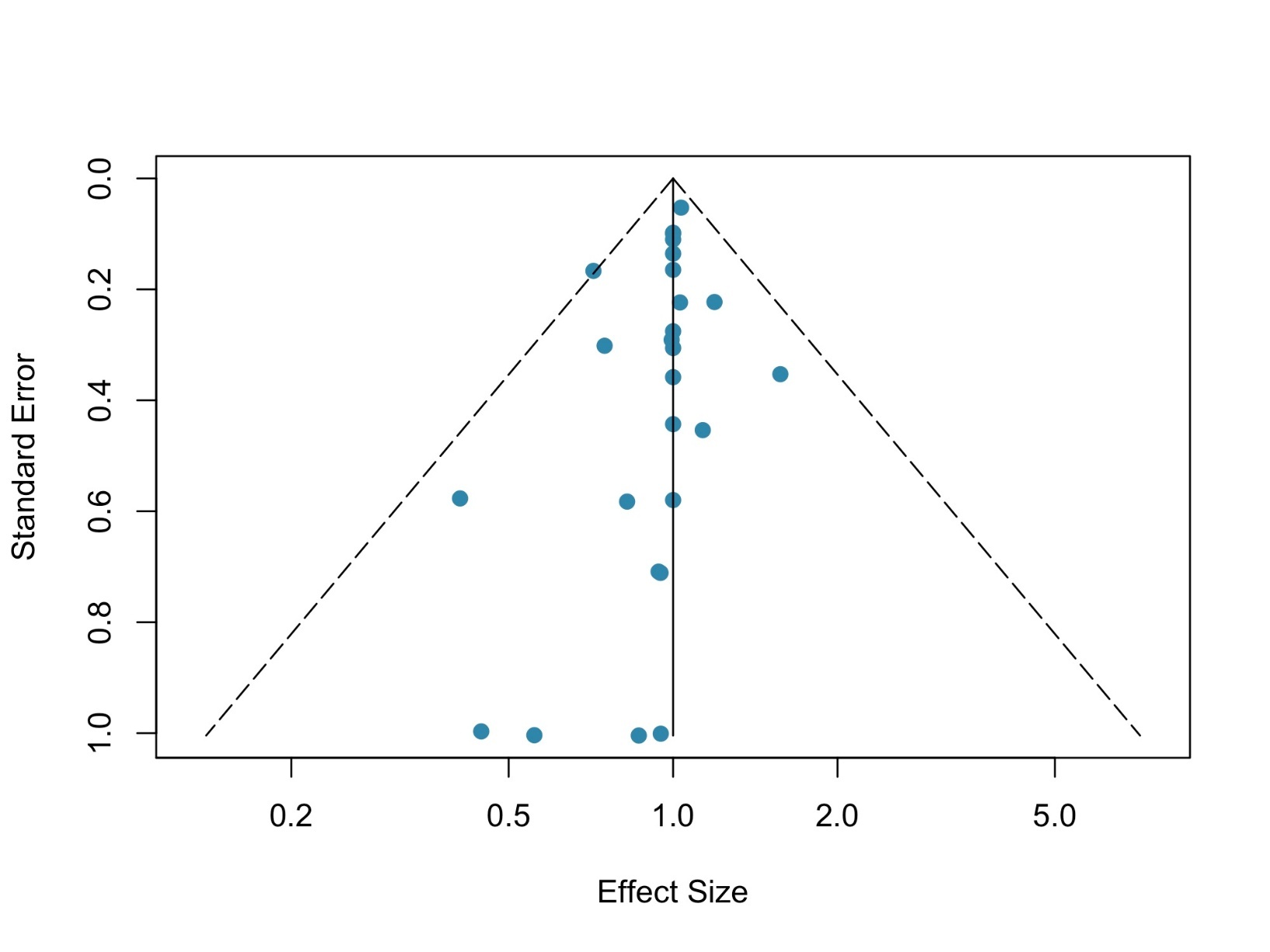


## Figure S6.9: Any Adverse Event


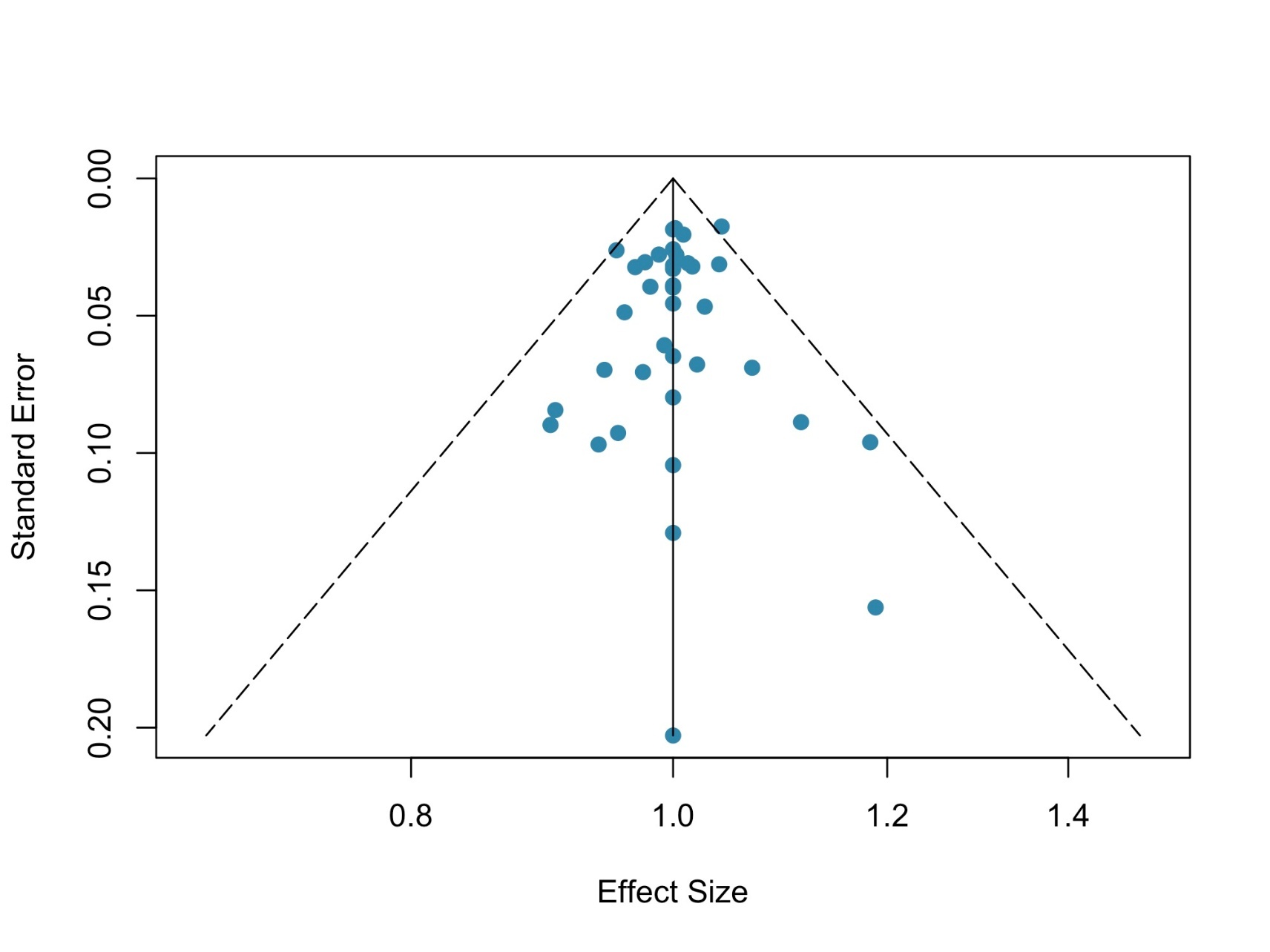


## Figure S6.10: Serious Adverse Event


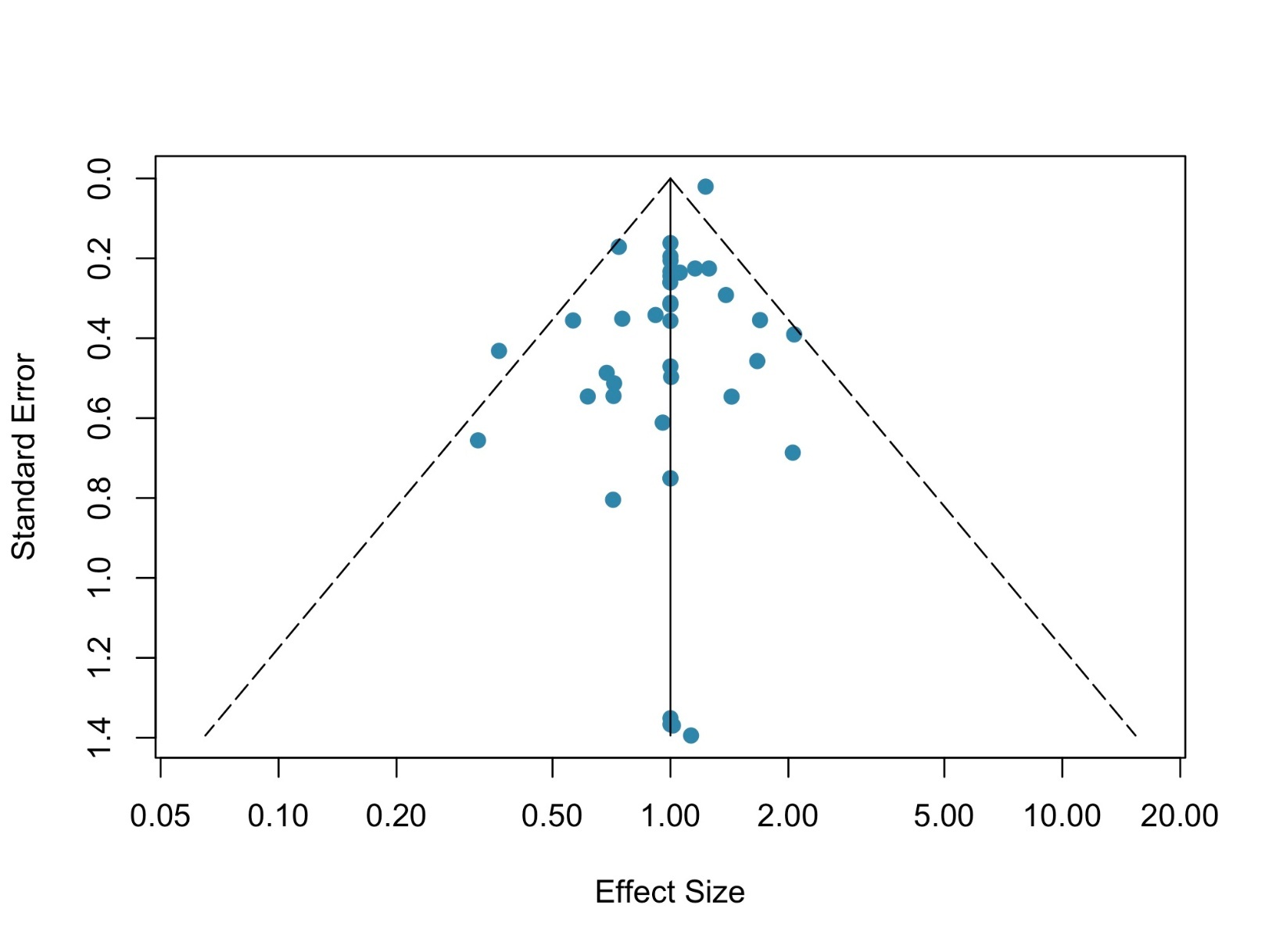


## Figure S6.11: GI Adverse Event


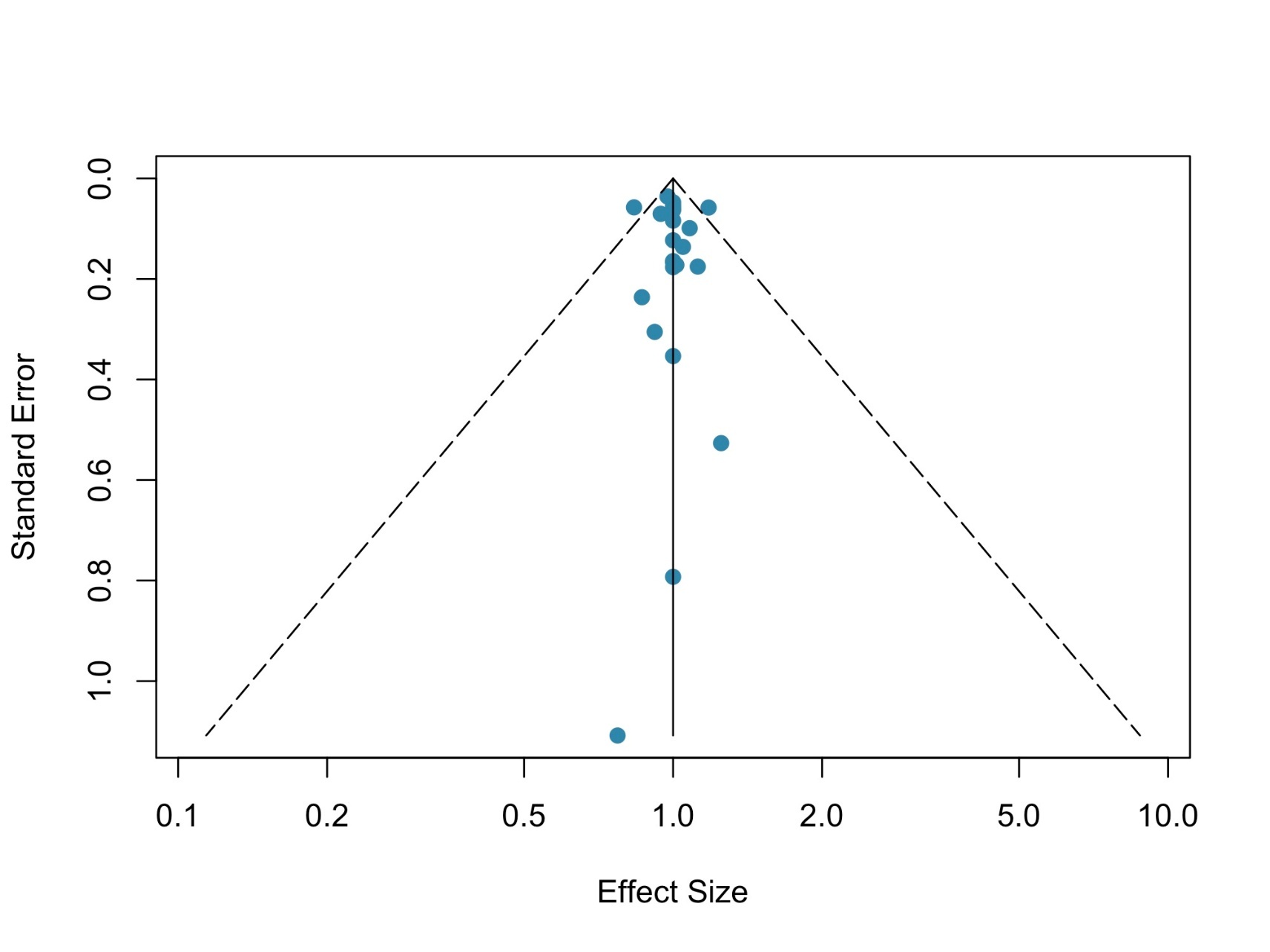


## Figure S6.12: Adverse Event Leading to Treatment Discontinuation


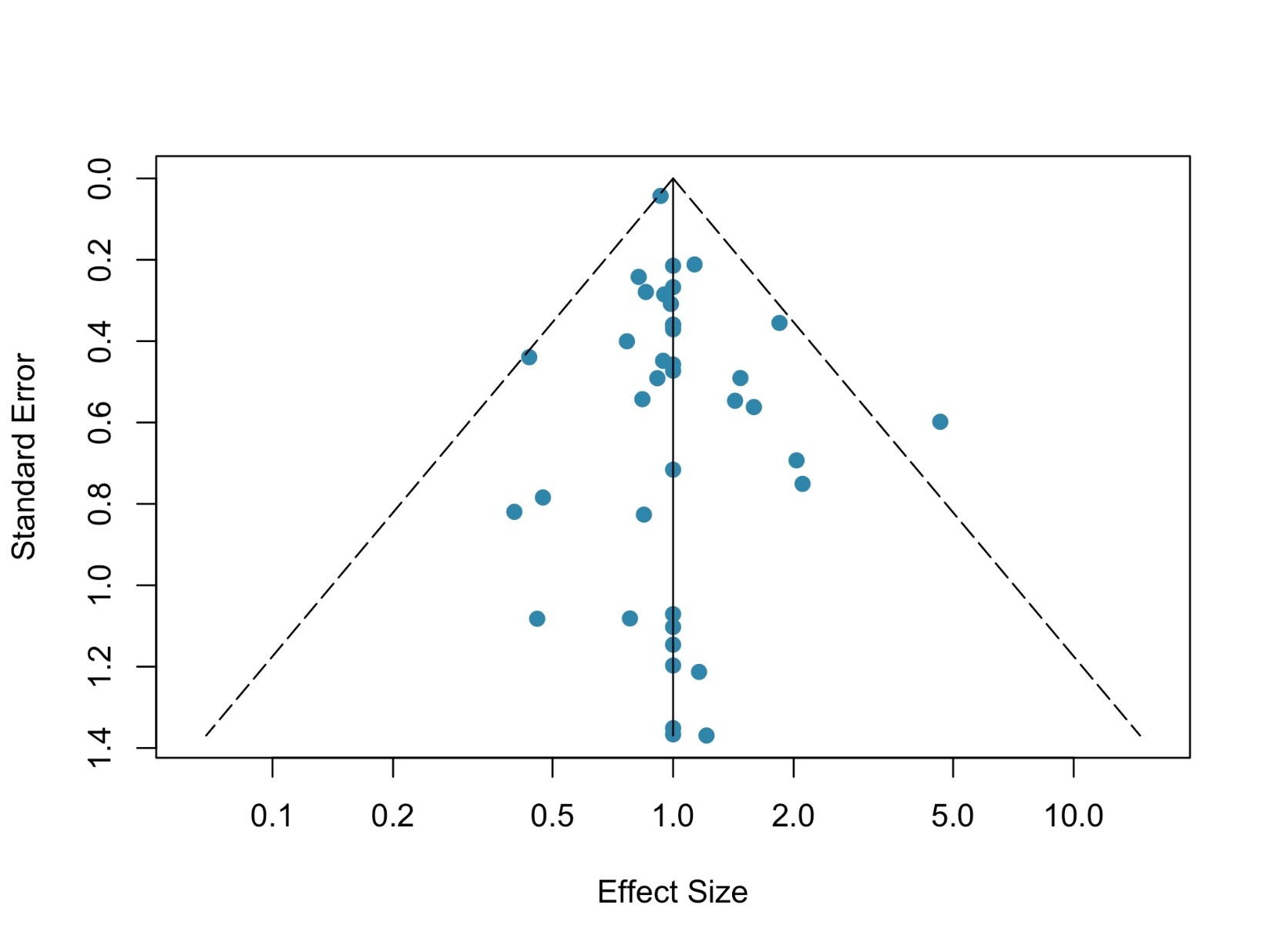


## Figure S6.13: Change in HDL Cholesterol

**
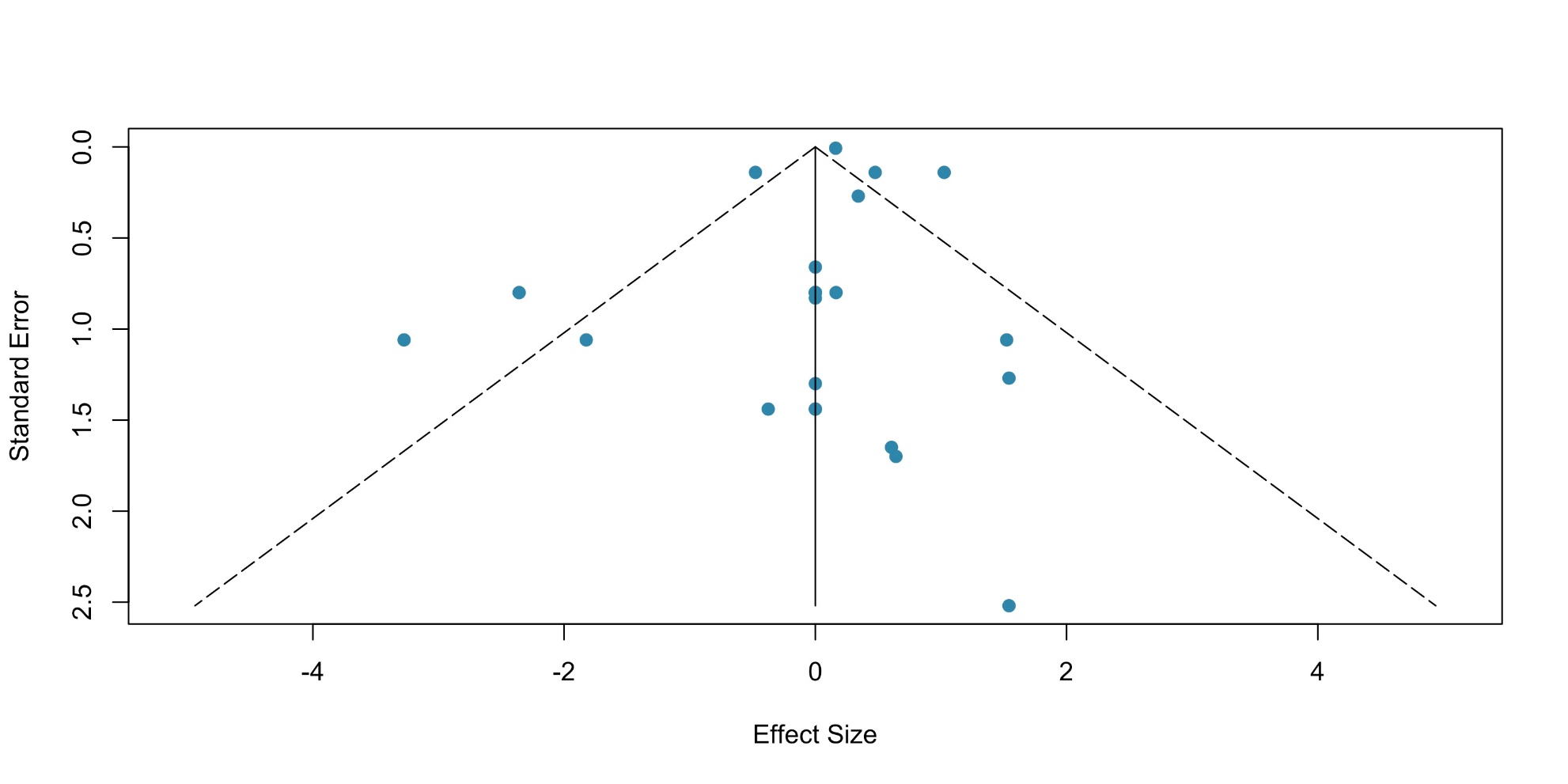
**

# Supplement S7: League Tables

## Table S7.1: Percent Change in Body Weight League

|  | Cagrilintide 2.4 mg | Cagrilintide 2.4 mg-Semaglutide 2.4 mg | Cagrilintide 4.5 mg | Cagrilintide 4.5 mg-Semaglutide 2.4 mg | Oral Semaglutide 14 mg | Oral Semaglutide 25 mg | Oral Semaglutide 50 mg | Placebo | Semaglutide 2.4 mg | Semaglutide 7.2mg | Tirzepatide 10 mg | Tirzepatide 15 mg |
| --- | --- | --- | --- | --- | --- | --- | --- | --- | --- | --- | --- | --- |
| Cagrilintide 2.4 mg | Cagrilintide 2.4 mg | **8.90 [ 4.65; 13.15]** | 1.10 [ -3.28; 5.48] | . | . | . | . | **-7.57 [-10.63; -4.52]** | 3.40 [ -0.85; 7.65] | . | . | . |
| Cagrilintide 2.4 mg-Semaglutide 2.4 mg | **9.77 [ 6.12; 13.41]** | Cagrilintide 2.4 mg-Semaglutide 2.4 mg | . | 0.00 [ -5.60; 5.60] | . | . | . | **-17.30 [-21.45; -13.15]** | **-6.19 [ -9.58; -2.81]** | . | . | . |
| Cagrilintide 4.5 mg | 0.60 [ -3.44; 4.65] | **-9.16 [-14.04; -4.29]** | Cagrilintide 4.5 mg | . | . | . | . | **-7.80 [-12.18; -3.42]** | . | . | . | . |
| Cagrilintide 4.5 mg-Semaglutide 2.4 mg | **10.15 [3.91; 16.39]** | 0.38 [ -5.06; 5.83] | **9.55 [ 2.54; 16.55]** | Cagrilintide 4.5 mg-Semaglutide 2.4 mg | . | . | . | . | **-7.40 [-14.11; -0.69]** | . | . | . |
| Oral Semaglutide 14 mg | 0.37 [ -4.91; 5.64] | **-9.40 [-14.82; -3.98]** | -0.24 [ -6.25; 5.78] | **-9.78 [-17.15; -2.42]** | Oral Semaglutide 14 mg | . | . | **-8.06 [-12.51; -3.61]** | . | . | . | . |
| Oral Semaglutide 25 mg | 3.71 [ -1.83; 9.24] | **-6.06 [-11.74; -0.38]** | 3.10 [ -3.14; 9.35] | -6.44 [-13.99; 1.11] | 3.34 [ -3.18; 9.86] | Oral Semaglutide 25 mg | . | **-11.40 [-16.16; -6.64]** | . | . | . | . |
| Oral Semaglutide 50 mg | 3.55 [ -0.61; 7.71] | **-6.22 [-10.56; -1.88]** | 2.95 [ -2.12; 8.01] | -6.60 [-13.21; 0.01] | 3.18 [ -2.22; 8.58] | -0.16 [ -5.81; 5.49] | Oral Semaglutide 50 mg | **-11.24 [-14.29; -8.19]** | . | . | . | . |
| Placebo | -7.69 [-10.52; -4.87] | **-17.46 [-20.55; -14.37]** | -8.30 [-12.34; -4.25] | **-17.84 [-23.71; -11.98]** | **-8.06 [-12.51; -3.61]** | -11.40 [-16.16; -6.64] | -11.24 [-14.29; -8.19] | Placebo | **11.47 [10.07; 12.88]** | **14.80 [ 10.49; 19.11]** | **14.82 [ 12.10; 17.55]** | **18.03 [ 16.14; 19.93]** |
| Semaglutide 2.4 mg | **3.72 [ 0.79; 6.66]** | **-6.05 [ -9.09; -3.00]** | 3.12 [ -1.07; 7.31] | **-6.43 [-12.23; -0.62]** | 3.35 [ -1.29; 8.00] | 0.01 [ -4.93; 4.96] | 0.17 [ -3.15; 3.50] | **11.41 [10.08; 12.74]** | Semaglutide 2.4 mg | 3.10 [ -1.26; 7.46] | **.** | **6.20 [ 1.82; 10.58]** |
| Semaglutide 7.2mg | **6.97 [ 2.28; 11.66]** | -2.80 [ -7.61; 2.01] | **6.36 [ 0.83; 11.90]** | -3.18 [-10.09; 3.73] | **6.60 [ 0.73; 12.47]** | 3.26 [ -2.84; 9.36] | 3.42 [ -1.47; 8.31] | **14.66 [10.84; 18.48]** | 3.25 [ -0.58; 7.07] | Semaglutide 7.2mg | . | . |
| Tirzepatide 10 mg | **7.08 [ 3.30; 10.85]** | -2.69 [ -6.66; 1.28] | **6.47 [ 1.71; 11.23]** | -3.07 [ -9.44; 3.29] | **6.71 [ 1.59; 11.82]** | 3.37 [ -2.02; 8.75] | 3.53 [ -0.43; 7.48] | **14.77 [12.25; 17.29]** | **3.35 [ 0.56; 6.15]** | 0.11 [ -4.45; 4.67] | Tirzepatide 10 mg | **3.25 [ 0.52; 5.97]** |
| Tirzepatide 15 mg | **10.28 [ 6.98; 13.57]** | 0.51 [ -3.00; 4.02] | **9.67 [ 5.27; 14.07]** | 0.12 [ -5.96; 6.21] | **9.91 [ 5.12; 14.69]** | **6.57 [ 1.49; 11.64]** | 6.73 [ 3.21; 10.24] | **17.97 [ 16.21; 19.72]** | **6.55 [ 4.48; 8.62]** | 3.31 [ -0.86; 7.48] | **3.20 [ 0.68; 5.71]** | Tirzepatide 15 mg |

## Table S7.2: Waist Circumference League

|  | Cagrilintide 2.4 mg | Cagrilintide 2.4 mg-Semaglutide 2.4 mg | Cagrilintide 4.5 mg | Oral Semaglutide 14 mg | Oral Semaglutide 25 mg | Oral Semaglutide 50 mg | Placebo | Semaglutide 2.4 mg | Semaglutide 7.2 mg | Tirzepatide 10 mg | Tirzepatide 15 mg |
| --- | --- | --- | --- | --- | --- | --- | --- | --- | --- | --- | --- |
| Cagrilintide 2.4 mg | Cagrilintide 2.4 mg | **6.60 [ 3.33; 9.87]** | 0.60 [ -3.04; 4.24] | . | . | . | -5.64 [ -8.07; -3.21] | 2.50 [ -0.77; 5.77] | . | . | . |
| Cagrilintide 2.4 mg-Semaglutide 2.4 mg | **7.13 [ 4.07; 10.19]** | Cagrilintide 2.4 mg-Semaglutide 2.4 mg | . | . | . | . | -13.40 [-16.67; -10.13] | **-4.10 [ -7.37; -0.83]** | . | . | . |
| Cagrilintide 4.5 mg | -0.06 [ -3.41; 3.29] | **-7.19 [-11.35; -3.04]** | Cagrilintide 4.5 mg | . | . | . | **-4.80 [ -8.44; -1.16]** | . | . | . | . |
| Oral Semaglutide 14 mg | 0.84 [ -3.26; 4.94] | **-6.30 [-10.73; -1.87]** | 0.90 [ -3.89; 5.69] | Oral Semaglutide 14 mg | . | . | -6.36 [ -9.78; -2.94] | . | . | . | . |
| Oral Semaglutide 25 mg | 3.98 [ -0.78; 8.74] | -3.16 [ -8.20; 1.89] | 4.04 [ -1.32; 9.40] | 3.14 [ -2.27; 8.55] | Oral Semaglutide 25 mg | . | -9.50 [-13.69; -5.31] | . | . | . | . |
| Oral Semaglutide 50 mg | **4.48 [ 0.44; 8.51]** | -2.66 [ -7.03; 1.71] | 4.54 [ -0.19; 9.27] | 3.64 [ -1.14; 8.42] | 0.50 [ -4.86; 5.86] | Oral Semaglutide 50 mg | **-10.00 [-13.34; -6.66]** | . | . | . | . |
| Placebo | -5.52 [ -7.78; -3.26] | **-12.66 [-15.47; -9.84]** | **-5.46 [ -8.81; -2.11]** | -6.36 [ -9.78; -2.94] | -9.50 [-13.69; -5.31] | -10.00 [-13.34; -6.66] | Placebo | **8.38 [ 7.22; 9.54]** | **11.70 [ 8.40; 15.00]** | **11.57 [ 9.44; 13.70]** | **14.16 [ 12.30; 16.03]** |
| Semaglutide 2.4 mg | **2.82 [ 0.46; 5.18]** | **-4.31 [ -7.15; -1.47]** | 2.88 [ -0.59; 6.36] | 1.99 [ -1.61; 5.58] | -1.15 [ -5.49; 3.18] | -1.65 [ -5.18; 1.87] | **8.35 [ 7.24; 9.45]** | Semaglutide 2.4 mg | 2.90 [ -0.56; 6.36] | . | **5.40 [ 1.92; 8.88]** |
| Semaglutide 7.2 mg | **5.97 [ 2.27; 9.67]** | -1.16 [ -5.21; 2.88] | **6.03 [ 1.56; 10.50]** | **5.13 [ 0.59; 9.68]** | 1.99 [ -3.15; 7.14] | 1.49 [ -2.99; 5.98] | **11.49 [ 8.51; 14.48]** | **3.15 [ 0.14; 6.16]** | Semaglutide 7.2 mg | . | . |
| Tirzepatide 10 mg | **6.10 [ 3.08; 9.12]** | -1.03 [ -4.48; 2.41] | **6.16 [ 2.25; 10.07]** | **5.26 [ 1.29; 9.24]** | 2.12 [ -2.53; 6.78] | 1.62 [ -2.29; 5.53] | **11.62 [ 9.60; 13.65]** | **3.28 [ 1.03; 5.52]** | 0.13 [ -3.46; 3.72] | Tirzepatide 10 mg | **2.38 [ 0.25; 4.51]** |
| Tirzepatide 15 mg | **8.55 [ 5.77; 11.32]** | 1.41 [ -1.82; 4.64] | **8.61 [ 4.88; 12.33]** | **7.71 [ 3.91; 11.51]** | **4.57 [ 0.06; 9.08]** | **4.07 [ 0.34; 7.80]** | **14.07 [ 12.41; 15.73]** | **5.72 [ 3.87; 7.58]** | 2.57 [ -0.81; 5.96] | **2.45 [ 0.42; 4.47]** | Tirzepatide 15 mg |

## Table S7.3: Body Mass Index (BMI) League

|  | Cagrilintide 2.4 mg | Cagrilintide 2.4 mg-Semaglutide 2.4 mg | Oral Semaglutide 14 mg | Oral Semaglutide 25 mg | Oral Semaglutide 50 mg | Placebo | Semaglutide 2.4 mg | Semaglutide 7.2 mg | Tirzepatide 10 mg | Tirzepatide 15 mg |
| --- | --- | --- | --- | --- | --- | --- | --- | --- | --- | --- |
| Cagrilintide 2.4 mg | Cagrilintide 2.4 mg | **3.60 [ 2.52; 4.68]** | . | . | . | **-3.00 [-4.08; -1.92]** | **1.20 [ 0.12; 2.28]** | . | . | . |
| Cagrilintide 2.4 mg-Semaglutide 2.4 mg | **3.60 [ 2.52; 4.68]** | Cagrilintide 2.4 mg-Semaglutide 2.4 mg | . | . | . | **-6.60 [-7.68; -5.52]** | **-2.40 [-3.48; -1.32]** | . | . | . |
| Oral Semaglutide 14 mg | -0.48 [-1.98; 1.03] | **-4.08 [-5.58; -2.57]** | Oral Semaglutide 14 mg | . | . | **-2.61 [-3.77; -1.45]** | . | . | . | . |
| Oral Semaglutide 25 mg | 1.21 [-0.44; 2.87] | **-2.39 [-4.04; -0.73]** | 1.69 [-0.08; 3.46] | Oral Semaglutide 25 mg | . | **-4.30 [-5.64; -2.96]** | . | . | . | . |
| Oral Semaglutide 50 mg | **1.61 [ 0.12; 3.11]** | **-1.99 [-3.48; -0.49]** | **2.09 [ 0.47; 3.71]** | 0.40 [-1.36; 2.16] | Oral Semaglutide 50 mg | **-4.70 [-5.84; -3.56]** | . | . | . | . |
| Placebo | -3.09 [-4.05; -2.12] | **-6.69 [-7.65; -5.72]** | **-2.61 [-3.77; -1.45]** | **-4.30 [-5.64; -2.96]** | **-4.70 [-5.84; -3.56]** | Placebo | **4.25 [ 3.76; 4.74]** | **5.80 [ 4.66; 6.94]** | **5.20 [ 3.90; 6.50]** | **8.08 [ 7.21; 8.96]** |
| Semaglutide 2.4 mg | **1.29 [ 0.32; 2.25]** | **-2.31 [-3.28; -1.35]** | **1.76 [ 0.52; 3.01]** | 0.07 [-1.35; 1.49] | -0.33 [-1.56; 0.90] | **4.37 [ 3.91; 4.84]** | Semaglutide 2.4 mg | 1.30 [ 0.11; 2.49] | . | **2.60 [ 1.41; 3.79]** |
| Semaglutide 7.2 mg | **2.66 [ 1.28; 4.03]** | -0.94 [-2.32; 0.43] | **3.13 [ 1.59; 4.67]** | 1.44 [-0.25; 3.13] | 1.04 [-0.49; 2.57] | **5.74 [ 4.72; 6.76]** | 1.37 [ 0.34; 2.40] | Semaglutide 7.2 mg | . | **.** |
| Tirzepatide 10 mg | **2.41 [ 0.89; 3.93]** | -1.19 [-2.71; 0.33] | **2.89 [ 1.23; 4.54]** | 1.20 [-0.60; 2.99] | 0.80 [-0.85; 2.44] | **5.50 [ 4.31; 6.68]** | 1.12 [-0.12; 2.37] | -0.25 [-1.80; 1.31] | Tirzepatide 10 mg | **1.90 [ 0.60; 3.20]** |
| Tirzepatide 15 mg | **4.61 [ 3.43; 5.78]** | 1.01 [-0.17; 2.18] | **5.08 [ 3.72; 6.45]** | **3.39 [ 1.86; 4.92]** | **2.99 [ 1.64; 4.34]** | **7.69 [ 6.97; 8.42]** | **3.32 [ 2.55; 4.09]** | **1.95 [ 0.73; 3.17]** | **2.20 [ 1.01; 3.38]** | Tirzepatide 15 mg |

## Table S7.4: Body Weight League

|  | Cagrilintide 2.4 mg | Cagrilintide 2.4 mg-Semaglutide 2.4 mg | Cagrilintide 4.5 mg | Cagrilintide 4.5 mg-Semaglutide 2.4 mg | Oral Semaglutide 14 mg | Oral Semaglutide 25 mg | Oral Semaglutide 50 mg | Placebo | Semaglutide 2.4 mg | Semaglutide 7.2 mg | Tirzepatide 10 mg | Tirzepatide 15 mg |
| --- | --- | --- | --- | --- | --- | --- | --- | --- | --- | --- | --- | --- |
| Cagrilintide 2.4 mg | Cagrilintide 2.4 mg | 9.40 [ 6.46; 12.34] | 1.30 [ -1.89; 4.49] | . | . | . | . | **-7.98 [-10.14; -5.82]** | **3.30 [ 0.36; 6.24]** | . | . | . |
| Cagrilintide 2.4 mg-Semaglutide 2.4 mg | **10.16 [ 7.59; 12.73]** | Cagrilintide 2.4 mg-Semaglutide 2.4 mg | . | 0.30 [ -4.17; 4.77] | . | . | . | **-18.30 [-21.10; -15.50]** | **-6.37 [ -8.83; -3.92]** | . | . | . |
| Cagrilintide 4.5 mg | 0.65 [ -2.29; 3.59] | -9.51 [-13.03; -6.00] | Cagrilintide 4.5 mg | . | . | . | . | **-8.20 [-11.39; -5.01]** | **.** | . | . | . |
| Cagrilintide 4.5 mg-Semaglutide 2.4 mg | **10.92 [ 6.53; 15.32]** | 0.76 [ -3.26; 4.78] | **10.27 [ 5.30; 15.24]** | Cagrilintide 4.5 mg-Semaglutide 2.4 mg | . | . | . | . | **-7.30 [-11.77; -2.83]** | . | . | . |
| Oral Semaglutide 14 mg | -1.27 [ -4.98; 2.44] | -11.43 [-15.26; -7.61] | -1.92 [ -6.21; 2.37] | **-12.19 [-17.35; -7.04]** | Oral Semaglutide 14 mg | . | . | **-6.93 [-10.05; -3.81]** | . | . | . | . |
| Oral Semaglutide 25 mg | 3.80 [ -0.40; 8.00] | -6.36 [-10.66; -2.07] | 3.15 [ -1.57; 7.86] | **-7.12 [-12.64; -1.61]** | **5.07 [ 0.24; 9.90]** | Oral Semaglutide 25 mg | . | **-12.00 [-15.69; -8.31]** | . | . | . | . |
| Oral Semaglutide 50 mg | **3.33 [ 0.37; 6.28]** | -6.84 [ -9.92; -3.75] | 2.68 [ -0.97; 6.32] | **-7.59 [-12.23; -2.96]** | **4.60 [ 0.80; 8.40]** | -0.47 [ -4.75; 3.80] | Oral Semaglutide 50 mg | **-11.53 [-13.69; -9.36]** | . | . | . | . |
| Placebo | -8.20 [-10.21; -6.19] | -18.36 [-20.57; -16.16] | **-8.85 [-11.79; -5.91]** | **-19.12 [-23.22; -15.02]** | -6.93 [-10.05; -3.81] | -12.00 [-15.69; -8.31] | **-11.53 [-13.69; -9.36]** | Placebo | 12.17 [ 11.01; 13.33] | **16.30 [ 13.22; 19.38]** | 14.50 [ 10.98; 18.02] | **22.38 [ 20.00; 24.77]** |
| Semaglutide 2.4 mg | **4.08 [ 1.97; 6.19]** | -6.08 [ -8.27; -3.90] | **3.43 [ 0.36; 6.50]** | **-6.84 [-10.86; -2.82]** | 5.35 [ 2.04; 8.66] | 0.28 [ -3.57; 4.13] | 0.75 [ -1.67; 3.18] | **12.28 [ 11.18; 13.38]** | Semaglutide 2.4 mg | **3.60 [ 0.41; 6.79]** | . | **7.30 [ 4.02; 10.58]** |
| Semaglutide 7.2 mg | **7.90 [ 4.53; 11.28]** | -2.26 [ -5.72; 1.20] | **7.25 [ 3.24; 11.27]** | -3.02 [ -7.88; 1.85] | **9.17 [ 5.00; 13.35]** | 4.10 [ -0.51; 8.72] | **4.58 [ 1.06; 8.10]** | **16.10 [ 13.33; 18.88]** | **3.82 [ 1.03; 6.61]** | Semaglutide 7.2 mg | . | **.** |
| Tirzepatide 10 mg | **7.36 [ 3.60; 11.12]** | -2.81 [ -6.66; 1.05] | **6.71 [ 2.37; 11.04]** | -3.57 [ -8.74; 1.61] | **8.63 [ 4.15; 13.10]** | 3.56 [ -1.33; 8.44] | **4.03 [ 0.17; 7.89]** | **15.56 [ 12.36; 18.76]** | 3.28 [ -0.05; 6.60] | -0.55 [ -4.76; 3.67] | Tirzepatide 10 mg | **4.80 [ 1.28; 8.32]** |
| Tirzepatide 15 mg | **13.21 [ 10.45; 15.97]** | **3.05 [ 0.17; 5.92]** | **12.56 [ 9.05; 16.08]** | 2.29 [ -2.19; 6.77] | **14.48 [ 10.79; 18.17]** | **9.41 [ 5.23; 13.59]** | **9.88 [ 6.96; 12.81]** | **21.41 [ 19.45; 23.38]** | **9.13 [ 7.08; 11.19]** | **5.31 [ 1.96; 8.65]** | **5.86 [ 2.66; 9.06]** | Tirzepatide 15 mg |

## Table S7.5: Achieving ≥ 5% weight loss League

|  | Cagrilintide 2.4 mg | Cagrilintide 2.4 mg-Semaglutide 2.4 mg | Cagrilintide 4.5 mg | Oral Semaglutide 14 mg | Oral Semaglutide 25 mg | Oral Semaglutide 50 mg | Placebo | Semaglutide 2.4 mg | Semaglutide 7.2 mg | Tirzepatide 10 mg | Tirzepatide 15 mg |
| --- | --- | --- | --- | --- | --- | --- | --- | --- | --- | --- | --- |
| Cagrilintide 2.4 mg | Cagrilintide 2.4 mg | 0.86 [0.61; 1.20] | 0.83 [0.58; 1.18] | . | . | . | **2.46 [1.86; 3.26]** | 0.94 [0.67; 1.32] | . | . | . |
| Cagrilintide 2.4 mg-Semaglutide 2.4 mg | 0.85 [0.62; 1.18] | Cagrilintide 2.4 mg-Semaglutide 2.4 mg | . | . | . | . | **2.92 [2.05; 4.15]** | 1.09 [0.78; 1.53] | . | . | . |
| Cagrilintide 4.5 mg | 0.83 [0.59; 1.16] | 0.97 [0.63; 1.51] | Cagrilintide 4.5 mg | . | . | . | **2.90 [1.85; 4.55]** | . | . | . | . |
| Oral Semaglutide 14 mg | 0.57 [0.30; 1.11] | 0.67 [0.34; 1.33] | 0.69 [0.34; 1.41] | Oral Semaglutide 14 mg | . | . | **4.25 [2.30; 7.84]** | . | . | . | . |
| Oral Semaglutide 25 mg | 0.90 [0.53; 1.54] | 1.06 [0.61; 1.84] | 1.09 [0.60; 1.97] | 1.57 [0.73; 3.40] | Oral Semaglutide 25 mg | . | **2.70 [1.69; 4.31]** | . | . | . | . |
| Oral Semaglutide 50 mg | 0.69 [0.43; 1.10] | 0.81 [0.50; 1.32] | 0.83 [0.49; 1.43] | 1.20 [0.58; 2.49] | 0.77 [0.42; 1.41] | Oral Semaglutide 50 mg | **3.53 [2.39; 5.22]** | . | . | . | . |
| Placebo | **2.44 [1.89; 3.16**] | **2.86 [2.12; 3.85]** | **2.94 [2.03; 4.25]** | **4.25 [2.30; 7.84]** | **2.70 [1.69; 4.31]** | **3.53 [2.39; 5.22**] | Placebo | **0.39 [0.33; 0.45]** | **0.37 [0.25; 0.54]** | **0.33 [0.26; 0.43]** | **0.26 [0.20; 0.32]** |
| Semaglutide 2.4 mg | 0.94 [0.72; 1.23] | 1.11 [0.82; 1.49] | 1.14 [0.78; 1.67] | 1.64 [0.88; 3.08] | 1.05 [0.64; 1.70] | 1.37 [0.90; 2.07] | **0.39 [0.34; 0.45]** | Semaglutide 2.4 mg | 0.99 [0.70; 1.39] | . | . |
| Semaglutide 7.2 mg | 0.92 [0.62; 1.36] | 1.07 [0.70; 1.64] | 1.10 [0.68; 1.78] | 1.60 [0.80; 3.18] | 1.01 [0.58; 1.78] | 1.32 [0.80; 2.19] | **0.38 [0.27; 0.52]** | 0.97 [0.71; 1.32] | Semaglutide 7.2 mg | . | . |
| Tirzepatide 10 mg | 0.69 [0.49; 0.99] | 0.81 [0.55; 1.19] | 0.84 [0.54; 1.30] | 1.21 [0.63; 2.33] | 0.77 [0.45; 1.30] | 1.00 [0.63; 1.59] | **0.28 [0.22; 0.36]** | **0.73 [0.56; 0.97]** | 0.76 [0.51; 1.13] | Tirzepatide 10 mg | 0.99 [0.81; 1.20] |
| Tirzepatide 15 mg | 0.63 [0.45; 0.89] | 0.74 [0.51; 1.08] | 0.76 [0.50; 1.18] | 1.10 [0.58; 2.12] | 0.70 [0.42; 1.18] | 0.92 [0.58; 1.44] | **0.26 [0.21; 0.32]** | **0.67 [0.51; 0.88]** | 0.69 [0.47; 1.02] | 0.91 [0.75; 1.11] | Tirzepatide 15 mg |

## Table S7.6: Achieving ≥ 10% weight loss League

|  | Cagrilintide 2.4 mg-Semaglutide 2.4 mg | Cagrilintide 2.4 mg | Cagrilintide 4.5 mg | Oral Semaglutide 14 mg | Oral Semaglutide 25 mg | Oral Semaglutide 50 mg | Placebo | Semaglutide 2.4 mg | Semaglutide 7.2 mg | Tirzepatide 10 mg | Tirzepatide 15 mg |
| --- | --- | --- | --- | --- | --- | --- | --- | --- | --- | --- | --- |
| Cagrilintide 2.4 mg-Semaglutide 2.4 mg | Cagrilintide 2.4 mg-Semaglutide 2.4 mg | 1.52 [0.92; 2.51] | . | . | . | . | **5.83 [3.46; 9.83]** | 1.21 [0.73; 1.98] | . | . | . |
| Cagrilintide 2.4 mg | 1.49 [0.92; 2.41] | Cagrilintide 2.4 mg | 0.81 [0.46; 1.43] | . | . | . | **4.02 [2.58; 6.26]** | 0.79 [0.48; 1.32] | . | . | . |
| Cagrilintide 4.5 mg | 1.18 [0.60; 2.34] | 0.79 [0.46; 1.35] | Cagrilintide 4.5 mg | . | . | . | **5.50 [2.51; 12.07]** | . | . | . | . |
| Oral Semaglutide 14 mg | 1.31 [0.46; 3.70] | 0.88 [0.32; 2.44] | 1.11 [0.36; 3.38] | Oral Semaglutide 14 mg | . | . | **4.67 [1.82; 11.96]** | . | . | . | . |
| Oral Semaglutide 25 mg | 1.32 [0.57; 3.05] | 0.88 [0.39; 2.00] | 1.12 [0.44; 2.84] | 1.01 [0.31; 3.29] | Oral Semaglutide 25 mg | . | **4.63 [2.27; 9.47]** | . | . | . | . |
| Oral Semaglutide 50 mg | 0.97 [0.47; 2.03] | 0.65 [0.32; 1.33] | 0.82 [0.36; 1.91] | 0.74 [0.25; 2.26] | 0.74 [0.29; 1.86] | Oral Semaglutide 50 mg | 6.27 [3.48; 11.27] | . | . | . | . |
| Placebo | **6.11 [3.93; 9.49]** | **4.09 [2.75; 6.10]** | **5.17 [2.83; 9.42]** | 4.67 [1.82; 11.96] | **4.63 [2.27; 9.47]** | **6.27 [3.48; 11.27]** | Placebo | 0.21 [0.17; 0.26] | 0.22 [0.13; 0.40] | 0.20 [0.13; 0.30] | 0.14 [0.10; 0.19] |
| Semaglutide 2.4 mg | 1.18 [0.76; 1.83] | 0.79 [0.53; 1.19] | 1.00 [0.54; 1.84] | 0.90 [0.34; 2.36] | 0.90 [0.43; 1.88] | 1.21 [0.65; 2.25] | 0.19 [0.16; 0.24] | Semaglutide 2.4 mg | 0.91 [0.55; 1.49] | . | 1.37 [0.84; 2.24] |
| Semaglutide 7.2 mg | 1.17 [0.63; 2.18] | 0.79 [0.43; 1.43] | 0.99 [0.47; 2.10] | 0.90 [0.31; 2.56] | 0.89 [0.38; 2.09] | 1.20 [0.57; 2.55] | 0.19 [0.12; 0.31] | 0.99 [0.63; 1.57] | Semaglutide 7.2 mg | . | . |
| Tirzepatide 10 mg | 1.18 [0.68; 2.04] | 0.79 [0.47; 1.32] | 0.99 [0.50; 1.98] | 0.90 [0.33; 2.45] | 0.89 [0.40; 1.97] | 1.21 [0.61; 2.38] | 0.19 [0.14; 0.27] | 1.00 [0.69; 1.45] | 1.00 [0.57; 1.77] | Tirzepatide 10 mg | 0.92 [0.68; 1.23] |
| Tirzepatide 15 mg | 1.05 [0.63; 1.76] | 0.71 [0.44; 1.14] | 0.89 [0.46; 1.73] | 0.81 [0.30; 2.16] | 0.80 [0.37; 1.73] | 1.08 [0.56; 2.08] | 0.17 [0.13; 0.23] | 0.89 [0.65; 1.22] | 0.90 [0.53; 1.53] | 0.90 [0.67; 1.19] | Tirzepatide 15 mg |

## Table S7.7: Achieving ≥ 15% weight loss

|  | Cagrilintide 2.4 mg | Cagrilintide 2.4 mg-Semaglutide 2.4 mg | Oral Semaglutide 14 mg | Oral Semaglutide 25 mg | Oral Semaglutide 50 mg | Placebo | Semaglutide 2.4 mg | Semaglutide 7.2 mg | Tirzepatide 10 mg | Tirzepatide 15 mg |
| --- | --- | --- | --- | --- | --- | --- | --- | --- | --- | --- |
| Cagrilintide 2.4 mg | Cagrilintide 2.4 mg | 0.44 [0.26; 0.76] | . | . | . | **5.93 [3.18; 11.07]** | 0.66 [0.38; 1.15] | . | . | . |
| Cagrilintide 2.4 mg-Semaglutide 2.4 mg | 0.44 [0.26; 0.76] | Cagrilintide 2.4 mg-Semaglutide 2.4 mg | . | . | . | **13.36 [7.32; 24.38]** | 1.49 [0.88; 2.53] | . | . | . |
| Oral Semaglutide 14 mg | 0.38 [0.04; 3.13] | 0.85 [0.10; 7.00] | Oral Semaglutide 14 mg | . | . | **15.00 [1.92; 117.24]** | . | . | . | . |
| Oral Semaglutide 25 mg | 0.59 [0.19; 1.83] | 1.33 [0.43; 4.08] | 1.57 [0.16; 15.50] | Oral Semaglutide 25 mg | . | **9.55 [3.49; 26.16]** | . | . | . | . |
| Oral Semaglutide 50 mg | 0.56 [0.24; 1.35] | 1.27 [0.54; 2.99] | 1.50 [0.17; 13.20] | 0.96 [0.28; 3.27] | Oral Semaglutide 50 mg | **9.97 [4.96; 20.05]** | . | . | . | . |
| Placebo | **5.63 [3.34; 9.48]** | **12.68 [7.73; 20.79]** | **15.00 [1.92; 117.24]** | **9.55 [3.49; 26.16]** | **9.97 [4.96; 20.05]** | Placebo | 0.12 [0.09; 0.16] | 0.10 [0.05; 0.21] | 0.11 [0.06; 0.18] | 0.07 [0.05; 0.11] |
| Semaglutide 2.4 mg | 0.68 [0.41; 1.14] | 1.54 [0.95; 2.49] | 1.82 [0.23; 14.48] | 1.16 [0.41; 3.28] | 1.21 [0.58; 2.55] | 0.12 [0.09; 0.16] | Semaglutide 2.4 mg | 0.81 [0.48; 1.39] | . | 0.62 [0.37; 1.06] |
| Semaglutide 7.2 mg | 0.56 [0.28; 1.14] | 1.27 [0.64; 2.51] | 1.50 [0.18; 12.54] | 0.96 [0.31; 2.98] | 1.00 [0.42; 2.39] | 0.10 [0.06; 0.17] | 0.82 [0.50; 1.35] | Semaglutide 7.2 mg | . | . |
| Tirzepatide 10 mg | 0.54 [0.28; 1.04] | 1.22 [0.65; 2.28] | 1.45 [0.18; 11.77] | 0.92 [0.31; 2.73] | 0.96 [0.43; 2.16] | 0.10 [0.06; 0.15] | 0.79 [0.51; 1.23] | 0.96 [0.50; 1.84] | Tirzepatide 10 mg | 0.82 [0.59; 1.12] |
| Tirzepatide 15 mg | 0.42 [0.23; 0.77] | 0.94 [0.53; 1.69] | 1.12 [0.14; 8.99] | 0.71 [0.24; 2.07] | 0.74 [0.34; 1.62] | 0.07 [0.05; 0.11] | 0.61 [0.42; 0.89] | 0.74 [0.41; 1.36] | 0.77 [0.57; 1.06] | Tirzepatide 15 mg |

## Table S7.8: Achieving ≥ 20% weight loss

|  | Cagrilintide 2.4 mg | Cagrilintide 2.4 mg-Semaglutide 2.4 mg | Oral Semaglutide 25 mg | Oral Semaglutide 50 mg | Placebo | Semaglutide 2.4 mg | Semaglutide 7.2 mg | Tirzepatide 10 mg | Tirzepatide 15 mg |
| --- | --- | --- | --- | --- | --- | --- | --- | --- | --- |
| Cagrilintide 2.4 mg | Cagrilintide 2.4 mg | 0.29 [ 0.22; 0.38] | . | . | **8.44 [ 4.64; 15.37]** | 0.59 [ 0.43; 0.81] | . | . | . |
| Cagrilintide 2.4 mg-Semaglutide 2.4 mg | 0.29 [ 0.22; 0.38] | Cagrilintide 2.4 mg-Semaglutide 2.4 mg | . | . | **29.07 [16.94; 49.89]** | **2.02 [ 1.67; 2.45]** | . | . | . |
| Oral Semaglutide 25 mg | 0.85 [ 0.26; 2.84] | 2.94 [ 0.91; 9.49] | Oral Semaglutide 25 mg | . | 9.45 [ 3.03; 29.45] | . | . | . | . |
| Oral Semaglutide 50 mg | 0.61 [ 0.27; 1.35] | 2.09 [ 0.98; 4.45] | 0.71 [ 0.19; 2.70] | Oral Semaglutide 50 mg | 13.33 [ 6.61; 26.91] | . | . | . | . |
| Placebo | 8.08 [ 5.50; 11.87] | **27.82 [20.94; 36.95]** | **9.45 [ 3.03; 29.45]** | **13.33 [ 6.61; 26.91**] | Placebo | 0.07 [ 0.05; 0.09] | 0.06 [ 0.02; 0.13] | 0.06 [ 0.04; 0.09] | 0.05 [ 0.03; 0.07] |
| Semaglutide 2.4 mg | 0.59 [ 0.43; 0.81] | **2.03 [ 1.69; 2.44]** | 0.69 [ 0.22; 2.21] | 0.98 [ 0.46; 2.05] | 0.07 [ 0.06; 0.09] | Semaglutide 2.4 mg | 0.70 [ 0.56; 0.86] | . | 0.57 [ 0.47; 0.69] |
| Semaglutide 7.2 mg | 0.41 [ 0.28; 0.60] | **1.42 [ 1.08; 1.88]** | 0.48 [ 0.15; 1.57] | 0.68 [ 0.32; 1.47] | 0.05 [ 0.04; 0.07] | 0.70 [ 0.57; 0.86] | Semaglutide 7.2 mg | . | . |
| Tirzepatide 10 mg | 0.40 [ 0.28; 0.58] | **1.38 [ 1.05; 1.80]** | 0.47 [ 0.15; 1.51] | 0.66 [ 0.31; 1.41] | 0.05 [ 0.04; 0.07] | 0.68 [ 0.55; 0.83] | 0.97 [ 0.73; 1.30] | Tirzepatide 10 mg | 0.86 [ 0.78; 0.94] |
| Tirzepatide 15 mg | 0.34 [ 0.24; 0.49] | 1.17 [ 0.91; 1.51] | 0.40 [ 0.12; 1.28] | 0.56 [ 0.27; 1.19] | 0.04 [ 0.03; 0.05] | 0.58 [ 0.48; 0.69] | 0.83 [ 0.63; 1.09] | 0.85 [ 0.77; 0.94] | Tirzepatide 15 mg |

## Table S7.9: Any Adverse Events League

|  | Cagrilintide 2.4 mg | Cagrilintide 2.4 mg-Semaglutide 2.4 mg | Cagrilintide 4.5 mg | Cagrilintide 4.5 mg-Semaglutide 2.4 mg | Oral Semaglutide 14 mg | Oral Semaglutide 25 mg | Oral Semaglutide 50 mg | Placebo | Semaglutide 2.4 mg | Semaglutide 7.2 mg | Tirzepatide 10 mg | Tirzepatide 15 mg |
| --- | --- | --- | --- | --- | --- | --- | --- | --- | --- | --- | --- | --- |
| Cagrilintide 2.4 mg | Cagrilintide 2.4 mg | 0.91 [0.84; 0.99] | 0.88 [0.76; 1.01] | . | . | . | . | 1.05 [0.97; 1.13] | 0.94 [0.86; 1.02] | . | . | . |
| Cagrilintide 2.4 mg-Semaglutide 2.4 mg | 0.93 [0.86; 1.01] | Cagrilintide 2.4 mg-Semaglutide 2.4 mg | . | 1.01 [0.78; 1.31] | . | . | . | **1.12 [1.04; 1.21]** | **1.02 [0.95; 1.09]** | . | . | . |
| Cagrilintide 4.5 mg | 0.84 [0.74; 0.95] | 0.90 [0.78; 1.03] | Cagrilintide 4.5 mg | . | . | . | . | **1.33 [1.12; 1.57]** | . | . | . | . |
| Cagrilintide 4.5 mg-Semaglutide 2.4 mg | 1.01 [0.81; 1.26] | 1.08 [0.87; 1.34] | 1.20 [0.94; 1.54] | Cagrilintide 4.5 mg-Semaglutide 2.4 mg | . | . | . | **.** | 0.95 [0.77; 1.17] | . | . | . |
| Oral Semaglutide 14 mg | 0.53 [0.35; 0.79] | 0.56 [0.38; 0.85] | 0.63 [0.41; 0.96] | 0.52 [0.33; 0.82] | Oral Semaglutide 14 mg | . | . | **1.95 [1.30; 2.92]** | . | . | . | . |
| Oral Semaglutide 25 mg | 0.94 [0.83; 1.07] | 1.01 [0.89; 1.15] | 1.13 [0.95; 1.34] | 0.94 [0.74; 1.19] | **1.79 [1.18; 2.72]** | Oral Semaglutide 25 mg | . | 1.09 [0.97; 1.21] | . | . | . | . |
| Oral Semaglutide 50 mg | 0.94 [0.85; 1.05] | 1.01 [0.91; 1.12] | 1.13 [0.97; 1.31] | 0.94 [0.75; 1.17] | **1.79 [1.19; 2.70]** | 1.00 [0.87; 1.14] | Oral Semaglutide 50 mg | **1.09 [1.00; 1.18]** | . | . | . | . |
| Placebo | 1.02 [0.95; 1.10] | **1.10 [1.04; 1.17]** | **1.22 [1.07; 1.40]** | 1.02 [0.82; 1.26] | **1.95 [1.30; 2.92]** | 1.09 [0.97; 1.21] | **1.09 [1.00; 1.18]** | Placebo | 0.96 [0.93; 0.99] | 0.89 [0.80; 0.98] | 0.87 [0.81; 0.93] | 0.90 [0.85; 0.94] |
| Semaglutide 2.4 mg | 0.97 [0.91; 1.05] | 1.05 [0.99; 1.11] | **1.17 [1.02; 1.33]** | 0.97 [0.78; 1.19] | **1.86 [1.24; 2.78]** | 1.03 [0.92; 1.16] | 1.03 [0.95; 1.13] | 0.95 [0.92; 0.98] | Semaglutide 2.4 mg | 0.96 [0.88; 1.05] | . | 1.03 [0.93; 1.14] |
| Semaglutide 7.2 mg | 0.93 [0.83; 1.03] | 0.99 [0.90; 1.09] | 1.11 [0.95; 1.29] | 0.92 [0.74; 1.15] | **1.76 [1.17; 2.66]** | 0.98 [0.86; 1.12] | 0.98 [0.88; 1.10] | 0.90 [0.84; 0.98] | 0.95 [0.88; 1.03] | Semaglutide 7.2 mg | . | . |
| Tirzepatide 10 mg | 0.90 [0.82; 0.99] | 0.97 [0.89; 1.05] | 1.08 [0.93; 1.24] | 0.89 [0.72; 1.11] | 1.71 [1.14; 2.57] | 0.95 [0.84; 1.08] | 0.96 [0.86; 1.06] | 0.88 [0.83; 0.93] | 0.92 [0.86; 0.99] | 0.97 [0.88; 1.07] | Tirzepatide 10 mg | 1.03 [0.97; 1.10] |
| Tirzepatide 15 mg | 0.94 [0.86; 1.02] | 1.00 [0.93; 1.08] | 1.12 [0.97; 1.28] | 0.93 [0.75; 1.15] | 1.78 [1.19; 2.67] | 0.99 [0.88; 1.12] | 0.99 [0.91; 1.09] | 0.91 [0.87; 0.96] | 0.96 [0.91; 1.01] | 1.01 [0.93; 1.10] | 1.04 [0.98; 1.10] | Tirzepatide 15 mg |

## Table S7.10: Serious Adverse Events League

|  | Cagrilintide 2.4 mg | Cagrilintide 2.4 mg- Semaglutide 2.4 mg | Cagrilintide 4.5 mg | Cagrilintide 4.5 mg- Semaglutide 2.4 mg | Oral Semaglutide 25 mg | Oral Semaglutide 50 mg | Placebo | Semaglutide 2.4 mg | Semaglutide 7.2 mg | Tirzepatide 10 mg | Tirzepatide 15 mg |
| --- | --- | --- | --- | --- | --- | --- | --- | --- | --- | --- | --- |
| Cagrilintide 2.4 mg | Cagrilintide 2.4 mg | 0.91 [0.49; 1.69] | 0.74 [0.16; 3.49] | . | . | . | 1.39 [0.75; 2.57] | 1.80 [0.83; 3.92] | . | . | . |
| Cagrilintide 2.4 mg- Semaglutide 2.4 mg | 0.88 [0.48; 1.59] | Cagrilintide 2.4 mg- Semaglutide 2.4 mg | . | 0.92 [0.06; 13.53] | . | . | 1.60 [0.90; 2.85] | 1.97 [1.00; 3.89] | . | . | . |
| Cagrilintide 4.5 mg | 0.92 [0.24; 3.54] | 1.06 [0.26; 4.25] | Cagrilintide 4.5 mg | . | . | . | 1.33 [0.28; 6.27] | . | . | . | . |
| Cagrilintide 4.5 mg- Semaglutide 2.4 mg | 0.72 [0.07; 7.95] | 0.83 [0.08; 8.67] | 0.78 [0.05; 11.61] | Cagrilintide 4.5 mg- Semaglutide 2.4 mg | . | . | . | 2.18 [0.14; 33.16] | . | . | . |
| Oral Semaglutide 25 mg | 3.47 [1.06; 11.41] | 3.97 [1.25; 12.61] | 3.75 [0.69; 20.51] | 4.81 [0.37; 63.22] | Oral Semaglutide 25 mg | . | 0.44 [0.16; 1.25] | . | . | . | . |
| Oral Semaglutide 50 mg | 1.40 [0.57; 3.40] | 1.60 [0.68; 3.72] | 1.51 [0.34; 6.79] | 1.93 [0.17; 22.46] | 0.40 [0.12; 1.39] | Oral Semaglutide 50 mg | 1.10 [0.56; 2.17] | . | . | . | . |
| Placebo | 1.54 [0.86; 2.73] | **1.76 [1.06; 2.91]** | 1.66 [0.43; 6.35] | 2.13 [0.20; 22.45] | 0.44 [0.16; 1.25] | 1.10 [0.56; 2.17] | Placebo | 0.89 [0.70; 1.13] | 0.81 [0.37; 1.77] | 0.99 [0.59; 1.66] | 1.07 [0.74; 1.57] |
| Semaglutide 2.4 mg | 1.41 [0.78; 2.55] | 1.61 [0.96; 2.71] | 1.53 [0.39; 5.92] | 1.96 [0.19; 20.56] | 0.41 [0.14; 1.18] | 1.01 [0.49; 2.07] | 0.92 [0.73; 1.16] | Semaglutide 2.4 mg | 1.62 [0.83; 3.14] | . | 0.72 [0.31; 1.68] |
| Semaglutide 7.2 mg | 1.81 [0.80; 4.11] | 2.07 [0.96; 4.46] | 1.96 [0.45; 8.49] | 2.51 [0.22; 28.31] | 0.52 [0.16; 1.73] | 1.30 [0.53; 3.21] | 1.18 [0.65; 2.15] | 1.28 [0.71; 2.31] | Semaglutide 7.2 mg | . | . |
| Tirzepatide 10 mg | 1.38 [0.65; 2.91] | 1.57 [0.79; 3.15] | 1.49 [0.36; 6.19] | 1.91 [0.17; 21.12] | 0.40 [0.13; 1.25] | 0.99 [0.43; 2.27] | 0.90 [0.56; 1.45] | 0.98 [0.58; 1.65] | 0.76 [0.35; 1.63] | Tirzepatide 10 mg | 1.18 [0.70; 2.00] |
| Tirzepatide 15 mg | 1.51 [0.78; 2.96] | 1.73 [0.94; 3.18] | 1.64 [0.41; 6.54] | 2.10 [0.19; 22.66] | 0.44 [0.15; 1.31] | 1.09 [0.51; 2.33] | 0.99 [0.70; 1.40] | 1.07 [0.72; 1.59] | 0.84 [0.42; 1.65] | 1.10 [0.68; 1.78] | Tirzepatide 15 mg |
|  |  |  |  |  |  |  |  |  |  |  |  |

## Table S7.11: GI Adverse Events League

|  | Cagrilintide 2.4 mg | Cagrilintide 2.4 mg-Semaglutide 2.4 mg | Cagrilintide 4.5 mg | Cagrilintide 4.5 mg-Semaglutide 2.4 mg | Oral Semaglutide 25 mg | Oral Semaglutide 50 mg | Placebo | Semaglutide 2.4 mg | Semaglutide 7.2 mg | Tirzepatide 15 mg |
| --- | --- | --- | --- | --- | --- | --- | --- | --- | --- | --- |
| Cagrilintide 2.4 mg | Cagrilintide 2.4 mg | 0.68 [0.50; 0.92] | 0.80 [0.55; 1.17] | . | . | . | **1.43 [1.11; 1.86]** | 0.73 [0.54; 1.00] | . | . |
| Cagrilintide 2.4 mg-Semaglutide 2.4 mg | 0.72 [0.55; 0.94] | Cagrilintide 2.4 mg-Semaglutide 2.4 mg | . | 1.12 [0.73; 1.73] | . | . | **2.00 [1.48; 2.70]** | 1.11 [0.87; 1.40] | . | . |
| Cagrilintide 4.5 mg | 0.75 [0.53; 1.05] | 1.04 [0.70; 1.56] | Cagrilintide 4.5 mg | . | . | . | **2.00 [1.30; 3.08]** | . | . | . |
| Cagrilintide 4.5 mg-Semaglutide 2.4 mg | 0.81 [0.51; 1.29] | 1.13 [0.75; 1.70] | 1.08 [0.63; 1.87] | Cagrilintide 4.5 mg-Semaglutide 2.4 mg | . | . | . | 1.03 [0.66; 1.62] | . | . |
| Oral Semaglutide 25 mg | 0.76 [0.15; 3.77] | 1.07 [0.22; 5.26] | 1.02 [0.20; 5.16] | 0.94 [0.18; 4.84] | Oral Semaglutide 25 mg | . | 1.74 [0.36; 8.45] | . | . | . |
| Oral Semaglutide 50 mg | 0.69 [0.38; 1.28] | 0.97 [0.53; 1.78] | 0.93 [0.48; 1.82] | 0.86 [0.42; 1.74] | 0.91 [0.17; 4.87] | Oral Semaglutide 50 mg | 1.91 [1.09; 3.36] | . | . | . |
| Placebo | 1.33 [1.05; 1.69] | 1.85 [1.47; 2.34] | **1.78 [1.24; 2.56]** | **1.64 [1.07; 2.52]** | 1.74 [0.36; 8.45] | **1.91 [1.09; 3.36]** | Placebo | 0.65 [0.55; 0.76] | 0.60 [0.44; 0.84] | . |
| Semaglutide 2.4 mg | 0.85 [0.66; 1.09] | 1.18 [0.95; 1.47] | 1.13 [0.78; 1.66] | 1.05 [0.69; 1.58] | 1.11 [0.23; 5.43] | 1.22 [0.68; 2.19] | 0.64 [0.55; 0.75] | Semaglutide 2.4 mg | 0.86 [0.64; 1.18] | 0.82 [0.39; 1.73] |
| Semaglutide 7.2 mg | 0.76 [0.54; 1.09] | 1.07 [0.76; 1.50] | 1.02 [0.65; 1.61] | 0.94 [0.58; 1.54] | 1.00 [0.20; 4.98] | 1.10 [0.59; 2.07] | 0.57 [0.43; 0.76] | 0.90 [0.68; 1.19] | Semaglutide 7.2 mg | . |
| Tirzepatide 15 mg | 0.69 [0.32; 1.53] | 0.97 [0.44; 2.12] | 0.93 [0.40; 2.15] | 0.86 [0.36; 2.01] | 0.91 [0.16; 5.26] | 1.00 [0.39; 2.59] | 0.52 [0.24; 1.12] | 0.82 [0.39; 1.73] | 0.91 [0.41; 2.02] | Tirzepatide 15 mg |

## Table S7.12: Adverse Events Leading to Treatment Discontinuation League

|  | Cagrilintide 2.4 mg | Cagrilintide 2.4 mg-Semaglutide 2.4 mg | Cagrilintide 4.5 mg | Cagrilintide 4.5 mg-Semaglutide 2.4 mg | Oral Semaglutide 14 mg | Oral Semaglutide 25 mg | Oral Semaglutide 50 mg | Placebo | Semaglutide 1 mg | Semaglutide 2.4 mg | Semaglutide 7.2 mg | Tirzepatide 10 mg | Tirzepatide 15 mg |
| --- | --- | --- | --- | --- | --- | --- | --- | --- | --- | --- | --- | --- | --- |
| Cagrilintide 2.4 mg | Cagrilintide 2.4 mg | 0.45 [0.20; 0.98] | 5.94 [0.71; 49.85] | . | . | . | . | 0.97 [0.47; 2.02] | . | 0.73 [0.28; 1.90] | . | . | . |
| Cagrilintide 2.4 mg-Semaglutide 2.4 mg | 0.51 [0.25; 1.05] | Cagrilintide 2.4 mg-Semaglutide 2.4 mg | . | 0.92 [0.06; 13.24] | . | . | . | 1.67 [0.97; 2.88] | . | 1.65 [0.84; 3.24] | . | . | . |
| Cagrilintide 4.5 mg | 4.93 [0.61; 39.78] | **9.59 [1.12; 81.95]** | Cagrilintide 4.5 mg | . | . | . | . | 0.33 [0.03; 3.23] | . | . | . | . | . |
| Cagrilintide 4.5 mg-Semaglutide 2.4 mg | 0.36 [0.03; 3.98] | 0.69 [0.07; 7.11] | 0.07 [0.00; 1.67] | Cagrilintide 4.5 mg-Semaglutide 2.4 mg | . | . | . | . | . | 2.18 [0.15; 32.48] | . | . | . |
| Oral Semaglutide 14 mg | 0.28 [0.03; 2.84] | 0.55 [0.06; 5.19] | 0.06 [0.00; 1.22] | 0.80 [0.03; 19.72] | Oral Semaglutide 14 mg | . | . | 4.00 [0.45; 35.67] | . | . | . | . | . |
| Oral Semaglutide 25 mg | 0.98 [0.29; 3.31] | 1.91 [0.64; 5.70] | 0.20 [0.02; 2.08] | 2.77 [0.22; 34.96] | 3.45 [0.31; 38.02] | Oral Semaglutide 25 mg | . | 1.16 [0.43; 3.12] | . | . | . | . | . |
| Oral Semaglutide 50 mg | 0.72 [0.25; 2.08] | 1.40 [0.56; 3.50] | 0.15 [0.02; 1.41] | 2.03 [0.17; 23.93] | 2.53 [0.25; 25.91] | 0.74 [0.21; 2.60] | Oral Semaglutide 50 mg | 1.58 [0.72; 3.46] | . | . | . | . | . |
| Placebo | 1.14 [0.56; 2.31] | **2.22 [1.39; 3.54]** | 0.23 [0.03; 1.94] | 3.21 [0.31; 33.22] | 4.00 [0.45; 35.67] | 1.16 [0.43; 3.12] | 1.58 [0.72; 3.46] | Placebo | 0.50 [0.05; 5.36] | 0.53 [0.41; 0.68] | 0.19 [0.04; 0.79] | 0.44 [0.25; 0.76] | 0.45 [0.29; 0.69] |
| Semaglutide 1 mg | 0.57 [0.05; 6.77] | 1.11 [0.10; 12.43] | 0.12 [0.00; 2.79] | 1.61 [0.06; 44.82] | 2.00 [0.08; 50.40] | 0.58 [0.04; 7.58] | 0.79 [0.06; 9.60] | 0.50 [0.05; 5.36] | Semaglutide 1 mg | . | . | . | . |
| Semaglutide 2.4 mg | 0.58 [0.28; 1.19] | 1.12 [0.69; 1.82] | 0.12 [0.01; 0.99] | 1.62 [0.16; 16.73] | 2.02 [0.22; 18.27] | 0.59 [0.21; 1.62] | 0.80 [0.35; 1.81] | 0.51 [0.40; 0.64] | 1.01 [0.09; 10.96] | Semaglutide 2.4 mg | 0.74 [0.33; 1.66] | . | 1.30 [0.69; 2.43] |
| Semaglutide 7.2 mg | 0.37 [0.13; 1.02] | 0.72 [0.30; 1.71] | 0.07 [0.01; 0.71] | 1.04 [0.09; 11.95] | 1.29 [0.13; 13.09] | 0.38 [0.11; 1.30] | 0.51 [0.17; 1.52] | 0.32 [0.15; 0.69] | 0.65 [0.05; 7.79] | 0.64 [0.31; 1.33] | Semaglutide 7.2 mg | . | . |
| Tirzepatide 10 mg | 0.54 [0.23; 1.27] | 1.06 [0.55; 2.05] | 0.11 [0.01; 0.97] | 1.53 [0.14; 16.57] | 1.91 [0.20; 17.91] | 0.55 [0.18; 1.66] | 0.75 [0.30; 1.89] | 0.48 [0.30; 0.77] | 0.95 [0.08; 10.72] | 0.94 [0.57; 1.57] | 1.48 [0.61; 3.57] | Tirzepatide 10 mg | 1.01 [0.64; 1.60] |
| Tirzepatide 15 mg | 0.57 [0.26; 1.26] | 1.12 [0.63; 1.99] | 0.12 [0.01; 1.00] | 1.62 [0.15; 17.10] | 2.01 [0.22; 18.49] | 0.58 [0.20; 1.67] | 0.79 [0.33; 1.89] | 0.50 [0.35; 0.72] | 1.01 [0.09; 11.08] | 1.00 [0.68; 1.46] | 1.56 [0.69; 3.54] | 1.06 [0.68; 1.63] | Tirzepatide 15 mg |

## Table S7.13: Change in HDL Cholesterol League

| Cagrilintide 2.4 mg | Cagrilintide 2.4 mg-Semaglutide 2.4 mg | Cagrilintide 4.5 mg-Semaglutide 2.4 mg | Oral Semaglutide 14 mg | Oral Semaglutide 50 mg | Placebo | Semaglutide 2.4 mg | Tirzepatide 10 mg | Tirzepatide 15 mg |  |
| --- | --- | --- | --- | --- | --- | --- | --- | --- | --- |
| Cagrilintide 2.4 mg | Cagrilintide 2.4 mg | **-5.10 [ -7.18; -3.02]** | . | . | . | **3.80 [ 1.72; 5.88]** | -0.70 [ -2.78; 1.38] | . | . |
| Cagrilintide 2.4 mg-Semaglutide 2.4 mg | **-4.83 [ -6.82; -2.84]** | Cagrilintide 2.4 mg-Semaglutide 2.4 mg | 0.00 [ -3.13; 3.13] | . | . | **8.90 [ 6.82; 10.98]** | **4.24 [ 2.50; 5.97]** | . | . |
| Cagrilintide 4.5 mg-Semaglutide 2.4 mg | **-4.22 [ -7.44; -1.00]** | 0.61 [ -2.22; 3.44] | Cagrilintide 4.5 mg-Semaglutide 2.4 mg | . | . | . | **3.86 [ 0.73; 6.99]** | . | . |
| Oral Semaglutide 14 mg | 2.27 [ -0.53; 5.07] | **7.10 [ 4.42; 9.78]** | **6.49 [ 2.90; 10.08]** | Oral Semaglutide 14 mg | . | 0.31 [ -1.81; 2.43] | . | . | . |
| Oral Semaglutide 50 mg | -2.22 [ -5.64; 1.20] | 2.61 [ -0.71; 5.93] | 2.00 [ -2.09; 6.09] | -4.49 [ -8.08; -0.90] | Oral Semaglutide 50 mg | **4.80 [ 1.91; 7.69]** | . | . | . |
| Placebo | **2.58 [ 0.76; 4.40]** | **7.41 [ 5.78; 9.04]** | **6.80 [ 3.91; 9.69]** | 0.31 [ -1.81; 2.43] | **4.80 [ 1.91; 7.69]** | Placebo | **-2.14 [ -2.96; -1.32]** | **-5.13 [ -6.34; -3.91]** | **-6.18 [ -7.32; -5.03]** |
| Semaglutide 2.4 mg | 0.25 [ -1.56; 2.06] | **5.08 [ 3.50; 6.67]** | **4.47 [ 1.64; 7.30]** | **-2.02 [ -4.28; 0.24]** | 2.47 [ -0.52; 5.46] | **-2.33 [ -3.10; -1.56]** | Semaglutide 2.4 mg | . | **-2.80 [ -4.68; -0.92]** |
| Tirzepatide 10 mg | **-2.37 [ -4.51; -0.22]** | **2.46 [ 0.48; 4.44]** | 1.85 [ -1.24; 4.95] | **-4.64 [ -7.06; -2.22]** | -0.15 [ -3.26; 2.97] | **-4.95 [ -6.11; -3.78]** | **-2.62 [ -3.95; -1.28]** | Tirzepatide 10 mg | -1.12 [ -2.34; 0.09] |
| Tirzepatide 15 mg | **-3.31 [ -5.35; -1.27]** | 1.52 [ -0.34; 3.38] | 0.91 [ -2.11; 3.93] | **-5.58 [ -7.93; -3.24]** | -1.09 [ -4.15; 1.97] | **-5.89 [ -6.89; -4.89]** | **-3.56 [ -4.69; -2.44]** | -0.94 [ -2.11; 0.22] | Tirzepatide 15 mg |

# **Supplement S8: Treatment rankings (by P-score):**

## **S8.1: Percent Change in body weight**

| **Intervention** | **P-Score** |
| --- | --- |
| Tirzepatide 15 mg | 0.9141 |
| Cagrilintide 2.4 mg-Semaglutide 2.4 mg | 0.8721 |
| Cagrilintide 4.5 mg-Semaglutide 2.4 mg | 0.8715 |
| Tirzepatide 10 mg | 0.6927 |
| Semaglutide 7.2mg | 0.6858 |
| Semaglutide 2.4 mg | 0.4507 |
| Oral Semaglutide 25 mg | 0.4483 |
| Oral Semaglutide 50 mg | 0.4357 |
| Cagrilintide 4.5 mg | 0.2300 |
| Oral Semaglutide 14 mg | 0.2189 |
| Cagrilintide 2.4 mg | 0.1801 |
| Placebo | 0.000 |

**S8.2: Change in Waist Circumference**

| **Intervention** | **P-Score** |
| --- | --- |
| Tirzepatide 15 mg | 0.9688 |
| Cagrilintide 2.4 mg-Semaglutide 2.4 mg | 0.8399 |
| Tirzepatide 10 mg | 0.7414 |
| Semaglutide 7.2 mg | 0.7308 |
| Oral Semaglutide 50 mg | 0.5879 |
| Oral Semaglutide 25 mg | 0.5421 |
| Semaglutide 2.4 mg | 0.4303 |
| Oral Semaglutide 14 mg | 0.2655 |
| Cagrilintide 4.5 mg | 0.1999 |
| Cagrilintide 2.4 mg | 0.1935 |
| Placebo | 0.0001 |

## **S8.3: Change in BMI**

| **Intervention** | **P-Score** |
| --- | --- |
| Tirzepatide 15 mg | 0.9947 |
| Cagrilintide 2.4 mg-Semaglutide 2.4 mg | 0.8766 |
| Semaglutide 7.2 mg | 0.7298 |
| Tirzepatide 10 mg | 0.6816 |
| Oral Semaglutide 50 mg | 0.5129 |
| Semaglutide 2.4 mg | 0.4305 |
| Oral Semaglutide 25 mg | 0.4253 |
| Cagrilintide 2.4 mg | 0.2033 |
| Oral Semaglutide 14 mg | 0.1453 |
| Placebo | 0.0000 |

## **S8.4: Absolute change in bodyweight**

| **Intervention** | **P-Score** |
| --- | --- |
| Tirzepatide 15 mg | 0.9838 |
| Cagrilintide 4.5 mg-Semaglutide 2.4 mg | 0.8723 |
| Cagrilintide 2.4 mg-Semaglutide 2.4 mg | 0.8360 |
| Semaglutide 7.2 mg | 0.7058 |
| Tirzepatide 10 mg | 0.6763 |
| Semaglutide 2.4 mg | 0.4819 |
| Oral Semaglutide 25 mg | 0.4546 |
| Oral Semaglutide 50 mg | 0.4195 |
| Cagrilintide 4.5 mg | 0.2422 |
| Cagrilintide 2.4 mg | 0.1939 |
| Oral Semaglutide 14 mg | 0.1337 |
| Placebo | 0.0000 |

## **S5: Proportion of patients achieving ≥ 5% weight loss**

| **Intervention** | **P-Score** |
| --- | --- |
| Tirzepatide 15 mg | 0.8550 |
| Oral Semaglutide 14 mg | 0.8412 |
| Tirzepatide 10 mg | 0.7328 |
| Oral Semaglutide 50 mg | 0.7247 |
| Cagrilintide 4.5 mg | 0.5149 |
| Cagrilintide 2.4 mg-Semaglutide 2.4 mg | 0.4768 |
| Oral Semaglutide 25 mg | 0.4116 |
| Semaglutide 7.2 mg | 0.3787 |
| Semaglutide 2.4 mg | 0.3175 |
| Cagrilintide 2.4 mg | 0.2466 |
| Placebo | 0.0000 |

## **S8.6: Proportion of patients achieving ≥ 10% weight loss**

| **Intervention** | **P-Score** |
| --- | --- |
| Cagrilintide 2.4 mg-Semaglutide 2.4 mg | 0.7303 |
| Oral Semaglutide 50 mg | 0.7233 |
| Tirzepatide 15 mg | 0.6944 |
| Semaglutide 7.2 mg | 0.5461 |
| Cagrilintide 4.5 mg | 0.5458 |
| Tirzepatide 10 mg | 0.5329 |
| Semaglutide 2.4 mg | 0.5295 |
| Oral Semaglutide 14 mg | 0.4727 |
| Oral Semaglutide 25 mg | 0.4493 |
| Cagrilintide 2.4 mg | 0.2758 |
| Placebo | 0.0001 |

## **S8.7****: Proportion of patients achieving ≥ 15% weight loss**

| **Intervention** | **P-Score** |
| --- | --- |
| Tirzepatide 15 mg | 0.8129 |
| Cagrilintide 2.4 mg-Semaglutide 2.4 mg | 0.7451 |
| Oral Semaglutide 14 mg | 0.6896 |
| Tirzepatide 10 mg | 0.5717 |
| Semaglutide 7.2 mg | 0.5533 |
| Oral Semaglutide 50 mg | 0.5508 |
| Oral Semaglutide 25 mg | 0.5266 |
| Semaglutide 2.4 mg | 0.3690 |
| Cagrilintide 2.4 mg | 0.1805 |
| Placebo | 0.0005 |

## **S8.8: Proportion of patients achieving ≥ 20% weight loss**

| **Intervention** | **P-Score** |
| --- | --- |
| Cagrilintide 2.4 mg-Semaglutide 2.4 mg | 0.9765 |
| Tirzepatide 15 mg | 0.8614 |
| Tirzepatide 10 mg | 0.6685 |
| Semaglutide 7.2 mg | 0.6545 |
| Oral Semaglutide 50 mg | 0.4321 |
| Semaglutide 2.4 mg | 0.4074 |
| Oral Semaglutide 25 mg | 0.3109 |
| Cagrilintide 2.4 mg | 0.1886 |
| Placebo | 0.0000 |

## **S8.9: Any Adverse Event**

| **Intervention** | **P-Score** |
| --- | --- |
| Placebo | 0.9275 |
| Cagrilintide 2.4 mg | 0.8166 |
| Cagrilintide 4.5 mg-Semaglutide 2.4 mg | 0.7325 |
| Semaglutide 2.4 mg | 0.7160 |
| Oral Semaglutide 25 mg | 0.5206 |
| Oral Semaglutide 50 mg | 0.5149 |
| Tirzepatide 15 mg | 0.4806 |
| Cagrilintide 2.4 mg-Semaglutide 2.4 mg | 0.4464 |
| Semaglutide 7.2 mg | 0.4227 |
| Tirzepatide 10 mg | 0.2738 |
| Cagrilintide 4.5 mg | 0.1448 |
| Oral Semaglutide 14 mg | 0.0037 |

## **S8.10: Serious Adverse Events**

| **Intervention** | **P-Score** |
| --- | --- |
| Oral Semaglutide 25 mg | 0.9334 |
| Semaglutide 7.2 mg | 0.7302 |
| Placebo | 0.6345 |
| Tirzepatide 15 mg | 0.6045 |
| Oral Semaglutide 50 mg | 0.5203 |
| Semaglutide 2.4 mg | 0.5181 |
| Tirzepatide 10 mg | 0.5038 |
| Cagrilintide 4.5 mg | 0.3134 |
| Cagrilintide 4.5 mg- Semaglutide 2.4 mg | 0.3027 |
| Cagrilintide 2.4 mg | 0.2659 |
| Cagrilintide 2.4 mg- Semaglutide 2.4 mg | 0.1731 |

## **S8.11: GI Adverse Events**

| **Intervention** | **P-Score** |
| --- | --- |
| Placebo | 0.9635 |
| Cagrilintide 2.4 mg | 0.7697 |
| Semaglutide 2.4 mg | 0.5697 |
| Cagrilintide 4.5 mg-Semaglutide 2.4 mg | 0.4867 |
| Oral Semaglutide 25 mg | 0.4624 |
| Semaglutide 7.2 mg | 0.3994 |
| Cagrilintide 4.5 mg | 0.3753 |
| Tirzepatide 15 mg | 0.3511 |
| Oral Semaglutide 50 mg | 0.3261 |
| Cagrilintide 2.4 mg-Semaglutide 2.4 mg | 0.2962 |

## **S8.12: Adverse Events leading to treatment discontinuation**

| **Intervention** | **P-Score** |
| --- | --- |
| Cagrilintide 4.5 mg | 0.9522 |
| Placebo | 0.8051 |
| Cagrilintide 2.4 mg | 0.7251 |
| Oral Semaglutide 25 mg | 0.6910 |
| Oral Semaglutide 50 mg | 0.5518 |
| Semaglutide 1 mg | 0.4516 |
| Semaglutide 2.4 mg | 0.4247 |
| Tirzepatide 15 mg | 0.4203 |
| Tirzepatide 10 mg | 0.3787 |
| Cagrilintide 2.4 mg-Semaglutide 2.4 mg | 0.3399 |
| Cagrilintide 4.5 mg-Semaglutide 2.4 mg | 0.3148 |
| Oral Semaglutide 14 mg | 0.2479 |
| Semaglutide 7.2 mg | 0.1968 |

## **S8.12: Change in HDL Cholesterol**

| **Intervention** | **P-Score** |
| --- | --- |
| Cagrilintide 2.4 mg-Semaglutide 2.4 mg | 0.9426 |
| Cagrilintide 4.5 mg-Semaglutide 2.4 mg | 0.8454 |
| Tirzepatide 15 mg | 0.7542 |
| Oral Semaglutide 50 mg | 0.5967 |
| Tirzepatide 10 mg | 0.5881 |
| Cagrilintide 2.4 mg | 0.3339 |
| Semaglutide 2.4 mg | 0.3009 |
| Oral Semaglutide 14 mg | 0.0894 |
| Placebo | 0.0488 |

# **Supplement S9.** **CINeMA (Confidence in Network Meta-Analysis)**

## Table S9.1: Percent Change in Body Weight League

| Comparison | Within_Study_Bias | Reporting_Bias | Indirectness | Imprecision | Heterogeneity | Incoherence | Overall_Confidence | Reason_for_downgrade |
| --- | --- | --- | --- | --- | --- | --- | --- | --- |
| Tirzepatide 15 mg: Placebo | No concerns | No concerns | No concerns | Some concerns | Major concerns | No concerns | Low | Imprecision, Heterogeneity |
| Semaglutide 2.4 mg: Placebo | No concerns | No concerns | No concerns | Some concerns | Major concerns | No concerns | Low | Imprecision, Heterogeneity |
| Cagrilintide 2.4 mg-Semaglutide 2.4 mg: Placebo | No concerns | No concerns | No concerns | Some concerns | Major concerns | No concerns | Low | Imprecision, Heterogeneity |
| Cagrilintide 4.5 mg-Semaglutide 2.4 mg: Placebo | No concerns | No concerns | No concerns | Some concerns | Major concerns | No concerns | Low | Imprecision, Heterogeneity |
| Oral Semaglutide 50 mg: Placebo | No concerns | Some concerns | Some concerns | Some concerns | Major concerns | No concerns | Low | Reporting bias, Indirectness, Imprecision, Heterogeneity |
| Cagrilintide 2.4 mg: Placebo | No concerns | No concerns | No concerns | Some concerns | Major concerns | No concerns | Low | Imprecision, Heterogeneity |
| Tirzepatide 10 mg: Placebo | No concerns | No concerns | No concerns | Some concerns | Major concerns | No concerns | Low | Imprecision, Heterogeneity |
| Oral Semaglutide 14 mg: Placebo | Major concerns | Some concerns | Some concerns | Some concerns | Major concerns | No concerns | Very low | Within-study bias, Reporting bias, Indirectness, Imprecision, Heterogeneity |
| Cagrilintide 4.5 mg: Placebo | No concerns | No concerns | No concerns | Some concerns | Major concerns | No concerns | Low | Imprecision, Heterogeneity |
| Oral Semaglutide 25 mg: Placebo | No concerns | Some concerns | No concerns | Some concerns | Major concerns | No concerns | Low | Reporting bias, Imprecision, Heterogeneity |
| Semaglutide 7.2 mg: Placebo | No concerns | No concerns | No concerns | Some concerns | Major concerns | No concerns | Low | Imprecision, Heterogeneity |
| Semaglutide 2.4 mg: Tirzepatide 15 mg | Some concerns | No concerns | No concerns | Some concerns | Major concerns | No concerns | Low | Within-study bias, Imprecision, Heterogeneity |
| Cagrilintide 2.4 mg-Semaglutide 2.4 mg: Tirzepatide 15 mg | No concerns | No concerns | No concerns | Major concerns | Major concerns | No concerns | Very low | Imprecision, Heterogeneity |
| Cagrilintide 4.5 mg-Semaglutide 2.4 mg: Tirzepatide 15 mg | No concerns | No concerns | No concerns | Major concerns | Major concerns | No concerns | Very low | Imprecision, Heterogeneity |
| Oral Semaglutide 50 mg: Tirzepatide 15 mg | No concerns | No concerns | No concerns | Some concerns | Major concerns | No concerns | Low | Imprecision, Heterogeneity |
| Semaglutide 7.2 mg: Tirzepatide 15 mg | No concerns | No concerns | No concerns | Major concerns | Major concerns | No concerns | Very low | Imprecision, Heterogeneity |
| Cagrilintide 2.4 mg-Semaglutide 2.4 mg: Semaglutide 2.4 mg | No concerns | No concerns | No concerns | Some concerns | Major concerns | No concerns | Low | Imprecision, Heterogeneity |
| Cagrilintide 4.5 mg-Semaglutide 2.4 mg: Semaglutide 2.4 mg | No concerns | No concerns | No concerns | Some concerns | Major concerns | No concerns | Low | Imprecision, Heterogeneity |
| Cagrilintide 2.4 mg-Semaglutide 2.4 mg: Oral Semaglutide 50 mg | No concerns | No concerns | No concerns | Some concerns | Major concerns | No concerns | Low | Imprecision, Heterogeneity |
| Cagrilintide 2.4 mg-Semaglutide 2.4 mg: Semaglutide 7.2 mg | No concerns | No concerns | No concerns | Major concerns | Major concerns | No concerns | Very low | Imprecision, Heterogeneity |
| Cagrilintide 4.5 mg-Semaglutide 2.4 mg: Oral Semaglutide 50 mg | No concerns | No concerns | No concerns | Major concerns | Major concerns | No concerns | Very low | Imprecision, Heterogeneity |
| Cagrilintide 4.5 mg-Semaglutide 2.4 mg: Semaglutide 7.2mg | No concerns | No concerns | No concerns | Major concerns | Major concerns | No concerns | Very low | Imprecision, Heterogeneity |
| Oral Semaglutide 50 mg: Semaglutide 7.2mg | No concerns | No concerns | No concerns | Major concerns | Major concerns | No concerns | Very low | Imprecision, Heterogeneity |

## Table S9.2: Change in Waist Circumference

| **Tirzepatide 15 mg: Placebo** | No concerns | No concerns | No concerns | Some concerns | Major concerns | No concerns | Low | Imprecision, Heterogeneity |
| --- | --- | --- | --- | --- | --- | --- | --- | --- |
| **Semaglutide 2.4 mg: Placebo** | No concerns | No concerns | No concerns | Some concerns | Major concerns | No concerns | Low | Imprecision, Heterogeneity |
| **Cagrilintide 2.4 mg-Semaglutide 2.4 mg: Placebo** | No concerns | No concerns | No concerns | Some concerns | Major concerns | No concerns | Low | Imprecision, Heterogeneity |
| **Cagrilintide 2.4 mg: Placebo** | No concerns | No concerns | No concerns | Some concerns | Major concerns | No concerns | Low | Imprecision, Heterogeneity |
| **Tirzepatide 10 mg: Placebo** | No concerns | No concerns | No concerns | Some concerns | Major concerns | No concerns | Low | Imprecision, Heterogeneity |
| **Oral Semaglutide 14 mg: Placebo** | Major concerns | Some concerns | Some concerns | Some concerns | Major concerns | No concerns | Very low | Within-study bias, Reporting bias, Indirectness, Imprecision, Heterogeneity |
| **Oral Semaglutide 50 mg: Placebo** | No concerns | Some concerns | No concerns | Some concerns | Major concerns | No concerns | Low | Reporting bias, Imprecision, Heterogeneity |
| **Cagrilintide 4.5 mg: Placebo** | No concerns | No concerns | No concerns | Some concerns | Major concerns | No concerns | Low | Imprecision, Heterogeneity |
| **Oral Semaglutide 25 mg: Placebo** | No concerns | Some concerns | No concerns | Some concerns | Major concerns | No concerns | Low | Reporting bias, Imprecision, Heterogeneity |
| **Semaglutide 7.2 mg: Placebo** | No concerns | No concerns | No concerns | Some concerns | Major concerns | No concerns | Low | Imprecision, Heterogeneity |
| **Semaglutide 2.4 mg: Tirzepatide 15 mg** | Some concerns | No concerns | No concerns | Some concerns | Major concerns | No concerns | Low | Within-study bias, Imprecision, Heterogeneity |
| **Cagrilintide 2.4 mg-Semaglutide 2.4 mg: Tirzepatide 15 mg** | No concerns | No concerns | No concerns | Major concerns | Major concerns | No concerns | Very low | Imprecision, Heterogeneity |
| **Oral Semaglutide 50 mg: Tirzepatide 15 mg** | No concerns | No concerns | No concerns | Some concerns | Major concerns | No concerns | Low | Imprecision, Heterogeneity |
| **Semaglutide 7.2 mg: Tirzepatide 15 mg** | No concerns | No concerns | No concerns | Major concerns | Major concerns | No concerns | Very low | Imprecision, Heterogeneity |
| **Cagrilintide 2.4 mg-Semaglutide 2.4 mg: Semaglutide 2.4 mg** | No concerns | No concerns | No concerns | Some concerns | Major concerns | No concerns | Low | Imprecision, Heterogeneity |
| **Cagrilintide 2.4 mg-Semaglutide 2.4 mg: Oral Semaglutide 50 mg** | No concerns | Some concerns | No concerns | Major concerns | Major concerns | No concerns | Very low | Reporting bias, Imprecision, Heterogeneity |
| **Cagrilintide 2.4 mg-Semaglutide 2.4 mg: Semaglutide 7.2 mg** | No concerns | Some concerns | No concerns | Major concerns | Major concerns | No concerns | Very low | Reporting bias, Imprecision, Heterogeneity |
| **Oral Semaglutide 50 mg: Semaglutide 7.2 mg** | No concerns | No concerns | No concerns | Major concerns | Major concerns | No concerns | Very low | Imprecision, Heterogeneity |
|  |  |  |  |  |  |  |  |  |

## Table S9.3: Change in BMI

| Comparison | Within_Study_Bias | Reporting_Bias | Indirectness | Imprecision | Heterogeneity | Incoherence | Overall_Confidence | Reason_for_downgrade |
| --- | --- | --- | --- | --- | --- | --- | --- | --- |
| Tirzepatide 15 mg: Placebo | No concerns | Some concerns | No concerns | No concerns | Major concerns | No concerns | Low | Reporting bias, Heterogeneity |
| Semaglutide 2.4 mg: Placebo | No concerns | Some concerns | No concerns | No concerns | Major concerns | No concerns | Low | Reporting bias, Heterogeneity |
| Cagrilintide 2.4 mg-Semaglutide 2.4 mg: Placebo | No concerns | Some concerns | No concerns | No concerns | Major concerns | No concerns | Low | Reporting bias, Heterogeneity |
| Cagrilintide 2.4 mg: Placebo | No concerns | Some concerns | No concerns | No concerns | Major concerns | No concerns | Low | Reporting bias, Heterogeneity |
| Tirzepatide 10 mg: Placebo | No concerns | Some concerns | No concerns | Some concerns | Major concerns | No concerns | Low | Reporting bias, Imprecision, Heterogeneity |
| Oral Semaglutide 14 mg: Placebo | Major concerns | Some concerns | Some concerns | Some concerns | Major concerns | No concerns | Very low | Within-study bias, Reporting bias, Indirectness, Imprecision, Heterogeneity |
| Oral Semaglutide 50 mg: Placebo | No concerns | Some concerns | No concerns | Some concerns | Major concerns | No concerns | Low | Reporting bias, Imprecision, Heterogeneity |
| Oral Semaglutide 25 mg: Placebo | No concerns | Some concerns | No concerns | Some concerns | Major concerns | No concerns | Low | Reporting bias, Imprecision, Heterogeneity |
| Semaglutide 7.2 mg: Placebo | No concerns | Some concerns | No concerns | Some concerns | Major concerns | No concerns | Low | Reporting bias, Imprecision, Heterogeneity |
| Semaglutide 2.4 mg: Tirzepatide 15 mg | Some concerns | Some concerns | No concerns | No concerns | Major concerns | No concerns | Low | Within-study bias, Reporting bias, Heterogeneity |
| Cagrilintide 2.4 mg-Semaglutide 2.4 mg: Tirzepatide 15 mg | No concerns | Some concerns | No concerns | Major concerns | Major concerns | No concerns | Very low | Reporting bias, Imprecision, Heterogeneity |
| Oral Semaglutide 50 mg: Tirzepatide 15 mg | No concerns | No concerns | No concerns | Some concerns | Major concerns | No concerns | Low | Imprecision, Heterogeneity |
| Semaglutide 7.2 mg: Tirzepatide 15 mg | No concerns | Some concerns | No concerns | Some concerns | Major concerns | No concerns | Low | Reporting bias, Imprecision, Heterogeneity |
| Cagrilintide 2.4 mg-Semaglutide 2.4 mg: Semaglutide 2.4 mg | No concerns | Some concerns | No concerns | No concerns | Major concerns | No concerns | Low | Reporting bias, Heterogeneity |
| Oral Semaglutide 50 mg: Semaglutide 2.4 mg | No concerns | No concerns | No concerns | Major concerns | Major concerns | No concerns | Very low | Imprecision, Heterogeneity |
| Cagrilintide 2.4 mg-Semaglutide 2.4 mg: Oral Semaglutide 50 mg | Some concerns | Some concerns | No concerns | Some concerns | Major concerns | No concerns | Low | Within-study bias, Reporting bias, Imprecision, Heterogeneity |
| Cagrilintide 2.4 mg-Semaglutide 2.4 mg: Semaglutide 7.2 mg | Some concerns | Some concerns | No concerns | Major concerns | Major concerns | No concerns | Very low | Within-study bias, Reporting bias, Imprecision, Heterogeneity |

## Table S9.4: Absolute Change in body weight

| Comparison | Within_Study_Bias | Reporting_Bias | Indirectness | Imprecision | Heterogeneity | Incoherence | Overall_Confidence | Reason_for_downgrade |
| --- | --- | --- | --- | --- | --- | --- | --- | --- |
| Tirzepatide 15 mg: Placebo | No concerns | No concerns | No concerns | Some concerns | Major concerns | No concerns | Low | Imprecision, Heterogeneity |
| Cagrilintide 2.4 mg-Semaglutide 2.4 mg: Placebo | No concerns | No concerns | No concerns | Some concerns | Major concerns | No concerns | Low | Imprecision, Heterogeneity |
| Cagrilintide 4.5 mg-Semaglutide 2.4 mg: Placebo | No concerns | Some concerns | No concerns | Some concerns | Major concerns | No concerns | Low | Reporting bias, Imprecision, Heterogeneity |
| Oral Semaglutide 50 mg: Placebo | No concerns | Some concerns | Some concerns | Some concerns | Major concerns | No concerns | Low | Reporting bias, Indirectness, Imprecision, Heterogeneity |
| Semaglutide 2.4 mg: Placebo | No concerns | No concerns | No concerns | Some concerns | Major concerns | No concerns | Low | Imprecision, Heterogeneity |
| Cagrilintide 2.4 mg: Placebo | No concerns | No concerns | No concerns | Some concerns | Major concerns | No concerns | Low | Imprecision, Heterogeneity |
| Tirzepatide 10 mg: Placebo | No concerns | No concerns | No concerns | Some concerns | Major concerns | No concerns | Low | Imprecision, Heterogeneity |
| Oral Semaglutide 14 mg: Placebo | Major concerns | Some concerns | Some concerns | Some concerns | Major concerns | No concerns | Very low | Within-study bias, Reporting bias, Indirectness, Imprecision, Heterogeneity |
| Cagrilintide 4.5 mg: Placebo | No concerns | Some concerns | No concerns | Some concerns | Major concerns | No concerns | Low | Reporting bias, Imprecision, Heterogeneity |
| Semaglutide 7.2 mg: Placebo | No concerns | No concerns | No concerns | Some concerns | Major concerns | No concerns | Low | Imprecision, Heterogeneity |
| Oral Semaglutide 25 mg: Placebo | No concerns | Some concerns | No concerns | Some concerns | Major concerns | No concerns | Low | Reporting bias, Imprecision, Heterogeneity |
| Cagrilintide 2.4 mg-Semaglutide 2.4 mg: Tirzepatide 15 mg | No concerns | No concerns | No concerns | Some concerns | Major concerns | No concerns | Low | Imprecision, Heterogeneity |
| Cagrilintide 4.5 mg-Semaglutide 2.4 mg: Tirzepatide 15 mg | No concerns | No concerns | No concerns | Major concerns | Major concerns | No concerns | Very low | Imprecision, Heterogeneity |
| Oral Semaglutide 50 mg: Tirzepatide 15 mg | Some concerns | Some concerns | No concerns | Some concerns | Major concerns | No concerns | Low | Within-study bias, Reporting bias, Imprecision, Heterogeneity |
| Semaglutide 2.4 mg: Tirzepatide 15 mg | Some concerns | No concerns | No concerns | Some concerns | Major concerns | No concerns | Low | Within-study bias, Imprecision, Heterogeneity |
| Semaglutide 7.2 mg: Tirzepatide 15 mg | No concerns | Some concerns | No concerns | Some concerns | Major concerns | No concerns | Low | Reporting bias, Imprecision, Heterogeneity |
| Cagrilintide 2.4 mg-Semaglutide 2.4 mg: Cagrilintide 4.5 mg-Semaglutide 2.4 mg | No concerns | No concerns | No concerns | Major concerns | Major concerns | No concerns | Very low | Imprecision, Heterogeneity |
| Cagrilintide 2.4 mg-Semaglutide 2.4 mg: Oral Semaglutide 50 mg | Some concerns | Some concerns | No concerns | Some concerns | Major concerns | No concerns | Low | Within-study bias, Reporting bias, Imprecision, Heterogeneity |
| Cagrilintide 2.4 mg-Semaglutide 2.4 mg: Semaglutide 2.4 mg | No concerns | No concerns | No concerns | Some concerns | Major concerns | No concerns | Low | Imprecision, Heterogeneity |
| Cagrilintide 2.4 mg-Semaglutide 2.4 mg: Semaglutide 7.2 mg | Some concerns | Some concerns | No concerns | Major concerns | Major concerns | No concerns | Very low | Within-study bias, Reporting bias, Imprecision, Heterogeneity |
| Cagrilintide 4.5 mg-Semaglutide 2.4 mg: Oral Semaglutide 50 mg | Some concerns | Some concerns | No concerns | Some concerns | Major concerns | No concerns | Low | Within-study bias, Reporting bias, Imprecision, Heterogeneity |
| Cagrilintide 4.5 mg-Semaglutide 2.4 mg: Semaglutide 2.4 mg | No concerns | No concerns | No concerns | Some concerns | Major concerns | No concerns | Low | Imprecision, Heterogeneity |
| Cagrilintide 4.5 mg-Semaglutide 2.4 mg: Semaglutide 7.2 mg | Some concerns | Some concerns | No concerns | Major concerns | Major concerns | No concerns | Very low | Within-study bias, Reporting bias, Imprecision, Heterogeneity |

## Table S9.5: Proportion of Patients Achieving >5% Weight Loss

| Comparison | Within_Study_Bias | Reporting_Bias | Indirectness | Imprecision | Heterogeneity | Incoherence | Overall_Confidence | Reason_for_downgrade |
| --- | --- | --- | --- | --- | --- | --- | --- | --- |
| Semaglutide 2.4 mg: Placebo | No concerns | Some concerns | No concerns | Major concerns | No concerns | No concerns | Low | Reporting bias, Imprecision |
| Cagrilintide 2.4 mg-Semaglutide 2.4 mg: Placebo | No concerns | Some concerns | No concerns | No concerns | No concerns | No concerns | Moderate | Reporting bias |
| Cagrilintide 2.4 mg: Placebo | No concerns | Some concerns | No concerns | No concerns | No concerns | No concerns | Moderate | Reporting bias |
| Tirzepatide 10 mg: Placebo | No concerns | Some concerns | No concerns | No concerns | No concerns | No concerns | Moderate | Reporting bias |
| Tirzepatide 15 mg: Placebo | No concerns | Some concerns | No concerns | No concerns | No concerns | No concerns | Moderate | Reporting bias |
| Oral Semaglutide 14 mg: Placebo | Major concerns | Some concerns | Some concerns | No concerns | No concerns | No concerns | Low | Within-study bias, Reporting bias, Indirectness |
| Oral Semaglutide 50 mg: Placebo | No concerns | Some concerns | No concerns | No concerns | No concerns | No concerns | Moderate | Reporting bias |
| Cagrilintide 4.5 mg: Placebo | No concerns | Some concerns | No concerns | No concerns | No concerns | No concerns | Moderate | Reporting bias |
| Oral Semaglutide 25 mg: Placebo | No concerns | Some concerns | No concerns | No concerns | No concerns | No concerns | Moderate | Reporting bias |
| Semaglutide 7.2 mg: Placebo | No concerns | Some concerns | No concerns | Major concerns | No concerns | No concerns | Low | Reporting bias, Imprecision |
| Cagrilintide 2.4 mg-Semaglutide 2.4 mg: Semaglutide 2.4 mg | No concerns | Some concerns | No concerns | No concerns | No concerns | No concerns | Moderate | Reporting bias |
| Semaglutide 2.4 mg: Tirzepatide 15 mg | No concerns | No concerns | No concerns | No concerns | No concerns | No concerns | High |  |
| Oral Semaglutide 50 mg: Semaglutide 2.4 mg | No concerns | Some concerns | No concerns | No concerns | No concerns | No concerns | Moderate | Reporting bias |
| Cagrilintide 2.4 mg-Semaglutide 2.4 mg: Tirzepatide 15 mg | No concerns | No concerns | No concerns | No concerns | No concerns | No concerns | High |  |
| Cagrilintide 2.4 mg-Semaglutide 2.4 mg: Oral Semaglutide 50 mg | No concerns | Some concerns | No concerns | No concerns | No concerns | No concerns | Moderate | Reporting bias |
| Cagrilintide 2.4 mg-Semaglutide 2.4 mg: Semaglutide 7.2 mg | No concerns | Some concerns | No concerns | No concerns | No concerns | No concerns | Moderate | Reporting bias |
| Oral Semaglutide 50 mg: Tirzepatide 15 mg | No concerns | Some concerns | No concerns | No concerns | No concerns | No concerns | Moderate | Reporting bias |
| Semaglutide 7.2 mg: Tirzepatide 15 mg | No concerns | Some concerns | No concerns | No concerns | No concerns | No concerns | Moderate | Reporting bias |

## Table S9.6: Proportion of Patients Achieving >10% Weight Loss

| Comparison | Within_Study_Bias | Reporting_Bias | Indirectness | Imprecision | Heterogeneity | Incoherence | Overall_Confidence | Reason_for_downgrade |
| --- | --- | --- | --- | --- | --- | --- | --- | --- |
| Tirzepatide 15 mg: Placebo | No concerns | No concerns | No concerns | No concerns | Some concerns | No concerns | Moderate | Heterogeneity |
| Semaglutide 2.4 mg: Placebo | No concerns | No concerns | No concerns | No concerns | Some concerns | No concerns | Moderate | Heterogeneity |
| Cagrilintide 2.4 mg-Semaglutide 2.4 mg: Placebo | No concerns | No concerns | No concerns | No concerns | Some concerns | No concerns | Moderate | Heterogeneity |
| Cagrilintide 2.4 mg: Placebo | No concerns | No concerns | No concerns | No concerns | Some concerns | No concerns | Moderate | Heterogeneity |
| Tirzepatide 10 mg: Placebo | No concerns | No concerns | No concerns | No concerns | Some concerns | No concerns | Moderate | Heterogeneity |
| Oral Semaglutide 14 mg: Placebo | No concerns | Some concerns | No concerns | No concerns | Some concerns | No concerns | Moderate | Reporting bias, Heterogeneity |
| Oral Semaglutide 50 mg: Placebo | No concerns | Some concerns | No concerns | No concerns | Some concerns | No concerns | Moderate | Reporting bias, Heterogeneity |
| Cagrilintide 4.5 mg: Placebo | No concerns | No concerns | No concerns | No concerns | Some concerns | No concerns | Moderate | Heterogeneity |
| Oral Semaglutide 25 mg: Placebo | No concerns | Some concerns | No concerns | No concerns | Some concerns | No concerns | Moderate | Reporting bias, Heterogeneity |
| Semaglutide 7.2 mg: Placebo | No concerns | No concerns | No concerns | No concerns | Some concerns | No concerns | Moderate | Heterogeneity |
| Semaglutide 2.4 mg: Tirzepatide 15 mg | Some concerns | No concerns | No concerns | No concerns | Some concerns | No concerns | Moderate | Within-study bias, Heterogeneity |
| Cagrilintide 2.4 mg-Semaglutide 2.4 mg: Tirzepatide 15 mg | No concerns | No concerns | No concerns | No concerns | Some concerns | No concerns | Moderate | Heterogeneity |
| Oral Semaglutide 50 mg: Tirzepatide 15 mg | No concerns | No concerns | No concerns | No concerns | Some concerns | No concerns | Moderate | Heterogeneity |
| Semaglutide 7.2 mg: Tirzepatide 15 mg | No concerns | No concerns | No concerns | No concerns | Some concerns | No concerns | Moderate | Heterogeneity |
| Cagrilintide 2.4 mg-Semaglutide 2.4 mg: Semaglutide 2.4 mg | No concerns | No concerns | No concerns | No concerns | Some concerns | No concerns | Moderate | Heterogeneity |
| Oral Semaglutide 50 mg: Semaglutide 2.4 mg | No concerns | No concerns | No concerns | No concerns | Some concerns | No concerns | Moderate | Heterogeneity |
| Cagrilintide 2.4 mg-Semaglutide 2.4 mg: Oral Semaglutide 50 mg | No concerns | Some concerns | No concerns | No concerns | Some concerns | No concerns | Moderate | Reporting bias, Heterogeneity |
| Cagrilintide 2.4 mg-Semaglutide 2.4 mg: Semaglutide 7.2 mg | No concerns | Some concerns | No concerns | No concerns | Some concerns | No concerns | Moderate | Reporting bias, Heterogeneity |

## Table S9.7: Proportion of Patients Achieving >15% Weight Loss

| Comparison | Within_Study_Bias | Reporting_Bias | Indirectness | Imprecision | Heterogeneity | Incoherence | Overall_Confidence | Reason_for_downgrade |
| --- | --- | --- | --- | --- | --- | --- | --- | --- |
| Tirzepatide 15 mg: Placebo | No concerns | No concerns | No concerns | No concerns | Some concerns | No concerns | Moderate | Heterogeneity |
| Semaglutide 2.4 mg: Placebo | No concerns | No concerns | No concerns | No concerns | Some concerns | No concerns | Moderate | Heterogeneity |
| Cagrilintide 2.4 mg-Semaglutide 2.4 mg: Placebo | No concerns | No concerns | No concerns | No concerns | Some concerns | No concerns | Moderate | Heterogeneity |
| Cagrilintide 2.4 mg: Placebo | No concerns | No concerns | No concerns | No concerns | Some concerns | No concerns | Moderate | Heterogeneity |
| Tirzepatide 10 mg: Placebo | No concerns | No concerns | No concerns | No concerns | Some concerns | No concerns | Moderate | Heterogeneity |
| Oral Semaglutide 14 mg: Placebo | Major concerns | Some concerns | Some concerns | Some concerns | Some concerns | No concerns | Low | Within-study bias, Reporting bias, Indirectness, Imprecision, Heterogeneity |
| Oral Semaglutide 50 mg: Placebo | No concerns | Some concerns | No concerns | No concerns | Some concerns | No concerns | Moderate | Reporting bias, Heterogeneity |
| Oral Semaglutide 25 mg: Placebo | No concerns | Some concerns | No concerns | Some concerns | Some concerns | No concerns | Low | Reporting bias, Imprecision, Heterogeneity |
| Semaglutide 7.2 mg: Placebo | No concerns | No concerns | No concerns | No concerns | Some concerns | No concerns | Moderate | Heterogeneity |
| Semaglutide 2.4 mg: Tirzepatide 15 mg | Some concerns | No concerns | No concerns | No concerns | Some concerns | No concerns | Moderate | Within-study bias, Heterogeneity |
| Cagrilintide 2.4 mg-Semaglutide 2.4 mg: Tirzepatide 15 mg | No concerns | No concerns | No concerns | No concerns | Some concerns | No concerns | Moderate | Heterogeneity |
| Oral Semaglutide 50 mg: Tirzepatide 15 mg | No concerns | No concerns | No concerns | Major concerns | Some concerns | No concerns | Low | Imprecision, Heterogeneity |
| Semaglutide 7.2 mg: Tirzepatide 15 mg | No concerns | No concerns | No concerns | No concerns | Some concerns | No concerns | Moderate | Heterogeneity |
| Cagrilintide 2.4 mg-Semaglutide 2.4 mg: Semaglutide 2.4 mg | No concerns | No concerns | No concerns | No concerns | Some concerns | No concerns | Moderate | Heterogeneity |
| Oral Semaglutide 50 mg: Semaglutide 2.4 mg | No concerns | No concerns | No concerns | No concerns | Some concerns | No concerns | Moderate | Heterogeneity |
| Cagrilintide 2.4 mg-Semaglutide 2.4 mg: Oral Semaglutide 50 mg | No concerns | Some concerns | No concerns | No concerns | Some concerns | No concerns | Moderate | Reporting bias, Heterogeneity |
| Cagrilintide 2.4 mg-Semaglutide 2.4 mg: Oral Semaglutide 25 mg | No concerns | Some concerns | No concerns | Some concerns | Some concerns | No concerns | Low | Reporting bias, Imprecision, Heterogeneity |
| Cagrilintide 2.4 mg-Semaglutide 2.4 mg: Semaglutide 7.2 mg | No concerns | Some concerns | No concerns | No concerns | Some concerns | No concerns | Moderate | Reporting bias, Heterogeneity |

## Table S9.8: Proportion of Patients Achieving >20% Weight Loss

| Comparison | | Within_Study_Bias | Reporting_Bias | Indirectness | Imprecision | Heterogeneity | Incoherence | Overall_Confidence | Reason_for_downgrade |
| --- | --- | --- | --- | --- | --- | --- | --- | --- | --- |
| Tirzepatide 15 mg: Placebo | | No concerns | No concerns | No concerns | No concerns | No concerns | No concerns | High |  |
| Semaglutide 2.4 mg: Placebo | | No concerns | No concerns | No concerns | No concerns | No concerns | No concerns | High |  |
| Cagrilintide 2.4 mg-Semaglutide 2.4 mg: Placebo | | No concerns | No concerns | No concerns | No concerns | No concerns | No concerns | High |  |
| Cagrilintide 2.4 mg: Placebo | | No concerns | No concerns | No concerns | No concerns | No concerns | No concerns | High |  |
| Tirzepatide 10 mg: Placebo | | No concerns | No concerns | No concerns | No concerns | No concerns | No concerns | High |  |
| Oral Semaglutide 50 mg: Placebo | | No concerns | Some concerns | No concerns | No concerns | No concerns | No concerns | Moderate | Reporting bias |
| Oral Semaglutide 25 mg: Placebo | | No concerns | Some concerns | No concerns | Some concerns | No concerns | No concerns | Moderate | Reporting bias, Imprecision |
| Semaglutide 7.2 mg: Placebo | | No concerns | No concerns | No concerns | No concerns | No concerns | No concerns | High |  |
| Semaglutide 2.4 mg: Tirzepatide 15 mg | | Some concerns | No concerns | No concerns | No concerns | No concerns | No concerns | Moderate | Within-study bias |
| Cagrilintide 2.4 mg-Semaglutide 2.4 mg: Tirzepatide 15 mg | | No concerns | No concerns | No concerns | No concerns | No concerns | No concerns | High |  |
| Oral Semaglutide 50 mg: Tirzepatide 15 mg | | No concerns | No concerns | No concerns | Major concerns | No concerns | No concerns | Low | Imprecision |
| Semaglutide 7.2 mg: Tirzepatide 15 mg | | No concerns | No concerns | No concerns | No concerns | No concerns | No concerns | High |  |
| Cagrilintide 2.4 mg-Semaglutide 2.4 mg: Semaglutide 2.4 mg | | No concerns | No concerns | No concerns | No concerns | No concerns | No concerns | High |  |
|  |  |  |  |  |  |  |  |  |  |
| Cagrilintide 2.4 mg-Semaglutide 2.4 mg: Oral Semaglutide 50 mg | | No concerns | Some concerns | No concerns | No concerns | No concerns | No concerns | Moderate | Reporting bias |
| Cagrilintide 2.4 mg-Semaglutide 2.4 mg: Semaglutide 7.2 mg | | No concerns | Some concerns | No concerns | No concerns | No concerns | No concerns | Moderate | Reporting bias |
| Oral Semaglutide 50 mg: Semaglutide 7.2 mg | | No concerns | No concerns | No concerns | Major concerns | No concerns | No concerns | Low | Imprecision |

## Table S9.9: Any Adverse Events

| Comparison | Within_Study_Bias | Reporting_Bias | Indirectness | Imprecision | Heterogeneity | Incoherence | Overall_Confidence | Reason_for_downgrade |
| --- | --- | --- | --- | --- | --- | --- | --- | --- |
| Cagrilintide 2.4 mg: Placebo | No concerns | Some concerns | No concerns | No concerns | No concerns | No concerns | Moderate | Reporting bias |
| Cagrilintide 4.5 mg: Placebo | No concerns | Some concerns | No concerns | No concerns | No concerns | No concerns | Moderate | Reporting bias |
| Oral Semaglutide 14 mg: Placebo | Major concerns | Some concerns | Some concerns | No concerns | No concerns | No concerns | Low | Within-study bias, Reporting bias, Indirectness |
| Oral Semaglutide 50 mg: Placebo | No concerns | Some concerns | Some concerns | No concerns | No concerns | No concerns | Moderate | Reporting bias, Indirectness |
| Semaglutide 2.4 mg: Placebo | No concerns | Some concerns | No concerns | No concerns | No concerns | No concerns | Moderate | Reporting bias |
| Cagrilintide 2.4 mg-Semaglutide 2.4 mg: Placebo | No concerns | Some concerns | No concerns | No concerns | No concerns | No concerns | Moderate | Reporting bias |
| Cagrilintide 4.5 mg-Semaglutide 2.4 mg: Placebo | No concerns | No concerns | No concerns | No concerns | No concerns | No concerns | High |  |
| Tirzepatide 15 mg: Placebo | No concerns | No concerns | No concerns | No concerns | No concerns | No concerns | High |  |
| Tirzepatide 10 mg: Placebo | No concerns | No concerns | No concerns | No concerns | No concerns | No concerns | High |  |
| Semaglutide 7.2 mg: Placebo | No concerns | Some concerns | No concerns | No concerns | No concerns | No concerns | Moderate | Reporting bias |
| Oral Semaglutide 25 mg: Placebo | No concerns | Some concerns | No concerns | No concerns | No concerns | No concerns | Moderate | Reporting bias |
| Cagrilintide 2.4 mg-Semaglutide 2.4 mg: Oral Semaglutide 50 mg | No concerns | Some concerns | Some concerns | No concerns | No concerns | No concerns | Moderate | Reporting bias, Indirectness |
| Cagrilintide 4.5 mg-Semaglutide 2.4 mg: Oral Semaglutide 50 mg | No concerns | No concerns | No concerns | No concerns | No concerns | No concerns | High |  |
| Oral Semaglutide 50 mg: Tirzepatide 15 mg | No concerns | No concerns | No concerns | No concerns | No concerns | No concerns | High |  |
| Oral Semaglutide 50 mg: Semaglutide 7.2 mg | No concerns | No concerns | No concerns | No concerns | No concerns | No concerns | High |  |
| Cagrilintide 2.4 mg-Semaglutide 2.4 mg: Semaglutide 2.4 mg | No concerns | Some concerns | No concerns | No concerns | No concerns | No concerns | Moderate | Reporting bias |
| Cagrilintide 4.5 mg-Semaglutide 2.4 mg: Semaglutide 2.4 mg | No concerns | Some concerns | No concerns | No concerns | No concerns | No concerns | Moderate | Reporting bias |
| Semaglutide 2.4 mg: Tirzepatide 15 mg | Some concerns | No concerns | No concerns | No concerns | No concerns | No concerns | Moderate | Within-study bias |
| Cagrilintide 2.4 mg-Semaglutide 2.4 mg: Semaglutide 7.2 mg | No concerns | Some concerns | No concerns | No concerns | No concerns | No concerns | Moderate | Reporting bias |
| Cagrilintide 4.5 mg-Semaglutide 2.4 mg: Tirzepatide 15 mg | No concerns | Some concerns | No concerns | No concerns | No concerns | No concerns | Moderate | Reporting bias |
| Cagrilintide 4.5 mg-Semaglutide 2.4 mg: Semaglutide 7.2 mg | No concerns | Some concerns | No concerns | No concerns | No concerns | No concerns | Moderate | Reporting bias |

## Table S9.10: Serious Adverse Events

| Comparison | Within_Study_Bias | Reporting_Bias | Indirectness | Imprecision | Heterogeneity | Incoherence | Overall_Confidence | Reason_for_downgrade |
| --- | --- | --- | --- | --- | --- | --- | --- | --- |
| Tirzepatide 15 mg: Placebo | No concerns | No concerns | No concerns | No concerns | Some concerns | No concerns | Moderate | Heterogeneity |
| Semaglutide 2.4 mg: Placebo | No concerns | No concerns | No concerns | No concerns | Some concerns | No concerns | Moderate | Heterogeneity |
| Cagrilintide 2.4 mg- Semaglutide 2.4 mg: Placebo | No concerns | No concerns | No concerns | No concerns | Some concerns | No concerns | Moderate | Heterogeneity |
| Cagrilintide 4.5 mg- Semaglutide 2.4 mg: Placebo | No concerns | No concerns | No concerns | Major concerns | Some concerns | No concerns | Low | Imprecision, Heterogeneity |
| Cagrilintide 2.4 mg: Placebo | No concerns | No concerns | No concerns | No concerns | Some concerns | No concerns | Moderate | Heterogeneity |
| Tirzepatide 10 mg: Placebo | No concerns | No concerns | No concerns | No concerns | Some concerns | No concerns | Moderate | Heterogeneity |
| Oral Semaglutide 50 mg: Placebo | No concerns | Some concerns | No concerns | No concerns | Some concerns | No concerns | Moderate | Reporting bias, Heterogeneity |
| Cagrilintide 4.5 mg: Placebo | No concerns | No concerns | No concerns | Some concerns | Some concerns | No concerns | Moderate | Imprecision, Heterogeneity |
| Oral Semaglutide 25 mg: Placebo | No concerns | Some concerns | No concerns | Major concerns | Some concerns | No concerns | Low | Reporting bias, Imprecision, Heterogeneity |
| Semaglutide 7.2 mg: Placebo | No concerns | No concerns | No concerns | No concerns | Some concerns | No concerns | Moderate | Heterogeneity |
| Semaglutide 2.4 mg: Tirzepatide 15 mg | Some concerns | No concerns | No concerns | No concerns | Some concerns | No concerns | Moderate | Within-study bias, Heterogeneity |
| Cagrilintide 2.4 mg- Semaglutide 2.4 mg: Tirzepatide 15 mg | No concerns | No concerns | No concerns | No concerns | Some concerns | No concerns | Moderate | Heterogeneity |
| Cagrilintide 4.5 mg- Semaglutide 2.4 mg: Tirzepatide 15 mg | No concerns | No concerns | No concerns | Major concerns | Some concerns | No concerns | Low | Imprecision, Heterogeneity |
| Oral Semaglutide 50 mg: Tirzepatide 15 mg | No concerns | No concerns | No concerns | No concerns | Some concerns | No concerns | Moderate | Heterogeneity |
| Semaglutide 7.2 mg: Tirzepatide 15 mg | No concerns | No concerns | No concerns | No concerns | Some concerns | No concerns | Moderate | Heterogeneity |
| Cagrilintide 2.4 mg- Semaglutide 2.4 mg: Semaglutide 2.4 mg | No concerns | No concerns | No concerns | No concerns | Some concerns | No concerns | Moderate | Heterogeneity |
| Cagrilintide 4.5 mg- Semaglutide 2.4 mg: Semaglutide 2.4 mg | No concerns | No concerns | No concerns | Major concerns | Some concerns | No concerns | Low | Imprecision, Heterogeneity |
| Cagrilintide 2.4 mg- Semaglutide 2.4 mg: Oral Semaglutide 50 mg | No concerns | No concerns | No concerns | No concerns | Some concerns | No concerns | Moderate | Heterogeneity |
| Cagrilintide 4.5 mg- Semaglutide 2.4 mg: Oral Semaglutide 50 mg | No concerns | No concerns | No concerns | Major concerns | Some concerns | No concerns | Low | Imprecision, Heterogeneity |
| Cagrilintide 4.5 mg- Semaglutide 2.4 mg: Semaglutide 7.2 mg | No concerns | No concerns | No concerns | Major concerns | Some concerns | No concerns | Low | Imprecision, Heterogeneity |

## Table S9.11: GI Adverse Events

| Comparison | Within_Study_Bias | Reporting_Bias | Indirectness | Imprecision | Heterogeneity | Incoherence | Overall_Confidence | Reason_for_downgrade |
| --- | --- | --- | --- | --- | --- | --- | --- | --- |
| Cagrilintide 2.4 mg: Placebo | No concerns | Some concerns | No concerns | No concerns | No concerns | No concerns | Moderate | Reporting bias |
| Cagrilintide 4.5 mg: Placebo | No concerns | Some concerns | No concerns | No concerns | No concerns | No concerns | Moderate | Reporting bias |
| Oral Semaglutide 50 mg: Placebo | No concerns | Some concerns | Some concerns | No concerns | No concerns | No concerns | Moderate | Reporting bias, Indirectness |
| Semaglutide 2.4 mg: Placebo | No concerns | Some concerns | No concerns | No concerns | No concerns | No concerns | Moderate | Reporting bias |
| Cagrilintide 2.4 mg-Semaglutide 2.4 mg: Placebo | No concerns | Some concerns | No concerns | No concerns | No concerns | No concerns | Moderate | Reporting bias |
| Cagrilintide 4.5 mg-Semaglutide 2.4 mg: Placebo | No concerns | Some concerns | No concerns | No concerns | No concerns | No concerns | Moderate | Reporting bias |
| Tirzepatide 15 mg: Placebo | No concerns | Some concerns | No concerns | Major concerns | No concerns | No concerns | Low | Reporting bias, Imprecision |
| Semaglutide 7.2 mg: Placebo | No concerns | Some concerns | No concerns | No concerns | No concerns | No concerns | Moderate | Reporting bias |
| Oral Semaglutide 25 mg: Placebo | No concerns | Some concerns | No concerns | Major concerns | No concerns | No concerns | Low | Reporting bias, Imprecision |
| Cagrilintide 2.4 mg-Semaglutide 2.4 mg: Oral Semaglutide 50 mg | No concerns | Some concerns | Some concerns | No concerns | No concerns | No concerns | Moderate | Reporting bias, Indirectness |
| Cagrilintide 4.5 mg-Semaglutide 2.4 mg: Oral Semaglutide 50 mg | No concerns | No concerns | No concerns | No concerns | No concerns | No concerns | High |  |
| Oral Semaglutide 50 mg: Tirzepatide 15 mg | No concerns | No concerns | No concerns | No concerns | No concerns | No concerns | High |  |
| Cagrilintide 2.4 mg-Semaglutide 2.4 mg: Semaglutide 2.4 mg | No concerns | Some concerns | No concerns | No concerns | No concerns | No concerns | Moderate | Reporting bias |
| Cagrilintide 4.5 mg-Semaglutide 2.4 mg: Semaglutide 2.4 mg | No concerns | Some concerns | No concerns | No concerns | No concerns | No concerns | Moderate | Reporting bias |
| Semaglutide 2.4 mg: Tirzepatide 15 mg | Major concerns | Some concerns | No concerns | No concerns | No concerns | No concerns | Low | Within-study bias, Reporting bias |
| Semaglutide 2.4 mg: Semaglutide 7.2 mg | No concerns | Some concerns | No concerns | No concerns | No concerns | No concerns | Moderate | Reporting bias |
| Cagrilintide 2.4 mg-Semaglutide 2.4 mg: Tirzepatide 15 mg | No concerns | Some concerns | No concerns | No concerns | No concerns | No concerns | Moderate | Reporting bias |
| Cagrilintide 2.4 mg-Semaglutide 2.4 mg: Semaglutide 7.2 mg | No concerns | Some concerns | No concerns | No concerns | No concerns | No concerns | Moderate | Reporting bias |
| Cagrilintide 4.5 mg-Semaglutide 2.4 mg: Tirzepatide 15 mg | No concerns | Some concerns | No concerns | Major concerns | No concerns | No concerns | Low | Reporting bias, Imprecision |
| Cagrilintide 4.5 mg-Semaglutide 2.4 mg: Semaglutide 7.2 mg | No concerns | Some concerns | No concerns | No concerns | No concerns | No concerns | Moderate | Reporting bias |
| Semaglutide 7.2 mg: Tirzepatide 15 mg | No concerns | Some concerns | No concerns | No concerns | No concerns | No concerns | Moderate | Reporting bias |

## Table S9.12: Adverse Events Leading to Treatment Discontinuation

| Comparison | Within_Study_Bias | Reporting_Bias | Indirectness | Imprecision | Heterogeneity | Incoherence | Overall_Confidence | Reason_for_downgrade |
| --- | --- | --- | --- | --- | --- | --- | --- | --- |
| Tirzepatide 15 mg: Placebo | Major concerns | No concerns | No concerns | Major concerns | No concerns | No concerns | Very low | Within-study bias, Imprecision |
| Semaglutide 2.4 mg: Placebo | Major concerns | No concerns | No concerns | No concerns | No concerns | No concerns | Low | Within-study bias |
| Semaglutide 1 mg: Placebo | Major concerns | Some concerns | Some concerns | Major concerns | No concerns | No concerns | Very low | Within-study bias, Reporting bias, Indirectness, Imprecision |
| Cagrilintide 2.4 mg-Semaglutide 2.4 mg: Placebo | Major concerns | No concerns | No concerns | No concerns | No concerns | No concerns | Low | Within-study bias |
| Cagrilintide 4.5 mg-Semaglutide 2.4 mg: Placebo | Major concerns | No concerns | No concerns | Major concerns | No concerns | No concerns | Very low | Within-study bias, Imprecision |
| Cagrilintide 2.4 mg: Placebo | Major concerns | No concerns | No concerns | No concerns | No concerns | No concerns | Low | Within-study bias |
| Tirzepatide 10 mg: Placebo | Major concerns | No concerns | No concerns | Major concerns | No concerns | No concerns | Very low | Within-study bias, Imprecision |
| Oral Semaglutide 14 mg: Placebo | Major concerns | Some concerns | Major concerns | Some concerns | No concerns | No concerns | Very low | Within-study bias, Reporting bias, Indirectness, Imprecision |
| Oral Semaglutide 50 mg: Placebo | Major concerns | Some concerns | No concerns | No concerns | No concerns | No concerns | Low | Within-study bias, Reporting bias |
| Cagrilintide 4.5 mg: Placebo | Major concerns | No concerns | No concerns | Major concerns | No concerns | No concerns | Very low | Within-study bias, Imprecision |
| Oral Semaglutide 25 mg: Placebo | Major concerns | Some concerns | No concerns | No concerns | No concerns | No concerns | Low | Within-study bias, Reporting bias |
| Semaglutide 7.2 mg: Placebo | Major concerns | No concerns | No concerns | Major concerns | No concerns | No concerns | Very low | Within-study bias, Imprecision |
| Semaglutide 2.4 mg: Tirzepatide 15 mg | Major concerns | No concerns | Some concerns | No concerns | No concerns | No concerns | Low | Within-study bias, Indirectness |
| Cagrilintide 2.4 mg-Semaglutide 2.4 mg: Tirzepatide 15 mg | Major concerns | No concerns | No concerns | No concerns | No concerns | No concerns | Low | Within-study bias |
| Cagrilintide 4.5 mg-Semaglutide 2.4 mg: Tirzepatide 15 mg | Major concerns | No concerns | No concerns | Major concerns | No concerns | No concerns | Very low | Within-study bias, Imprecision |
| Semaglutide 7.2 mg: Tirzepatide 15 mg | Major concerns | No concerns | No concerns | No concerns | No concerns | No concerns | Low | Within-study bias |
| Cagrilintide 2.4 mg-Semaglutide 2.4 mg: Semaglutide 2.4 mg | Major concerns | No concerns | No concerns | No concerns | No concerns | No concerns | Low | Within-study bias |
| Cagrilintide 4.5 mg-Semaglutide 2.4 mg: Semaglutide 2.4 mg | Major concerns | No concerns | No concerns | Major concerns | No concerns | No concerns | Very low | Within-study bias, Imprecision |
| Oral Semaglutide 50 mg: Semaglutide 2.4 mg | Major concerns | No concerns | No concerns | Major concerns | No concerns | No concerns | Very low | Within-study bias, Imprecision |
| Semaglutide 2.4 mg: Semaglutide 7.2 mg | Major concerns | No concerns | No concerns | Major concerns | No concerns | No concerns | Very low | Within-study bias, Imprecision |
| Cagrilintide 2.4 mg-Semaglutide 2.4 mg: Tirzepatide 10 mg | Major concerns | Some concerns | No concerns | No concerns | No concerns | No concerns | Low | Within-study bias, Reporting bias |
| Cagrilintide 2.4 mg-Semaglutide 2.4 mg: Oral Semaglutide 50 mg | Major concerns | No concerns | No concerns | No concerns | No concerns | No concerns | Low | Within-study bias |
| Cagrilintide 2.4 mg-Semaglutide 2.4 mg: Semaglutide 7.2 mg | Major concerns | No concerns | No concerns | Major concerns | No concerns | No concerns | Very low | Within-study bias, Imprecision |
| Cagrilintide 4.5 mg-Semaglutide 2.4 mg: Oral Semaglutide 50 mg | Major concerns | No concerns | No concerns | Major concerns | No concerns | No concerns | Very low | Within-study bias, Imprecision |
| Cagrilintide 4.5 mg-Semaglutide 2.4 mg: Semaglutide 7.2 mg | Major concerns | No concerns | No concerns | Major concerns | No concerns | No concerns | Very low | Within-study bias, Imprecision |
| Oral Semaglutide 50 mg: Semaglutide 7.2 mg | Major concerns | No concerns | No concerns | Major concerns | No concerns | No concerns | Very low | Within-study bias, Imprecision |

## Table S9.12: Change in HDL Cholesterol

| Comparison | Within_Study_Bias | Reporting Bias | Indirectness | Imprecision | Heterogeneity | Incoherence | Overall_Confidence | Reason_for_downgrade |
| --- | --- | --- | --- | --- | --- | --- | --- | --- |
| Tirzepatide 15 mg: Placebo | No concerns | No concerns | No concerns | Some concerns | Major concerns | No concerns | Low | Imprecision, Heterogeneity |
| Cagrilintide 2.4 mg-Semaglutide 2.4 mg: Placebo | No concerns | No concerns | No concerns | Some concerns | Major concerns | No concerns | Low | Imprecision, Heterogeneity |
| Cagrilintide 4.5 mg-Semaglutide 2.4 mg: Placebo | No concerns | Some concerns | No concerns | Some concerns | Major concerns | No concerns | Low | Reporting bias, Imprecision, Heterogeneity |
| Semaglutide 2.4 mg: Placebo | No concerns | No concerns | No concerns | No concerns | Major concerns | No concerns | Low | Heterogeneity |
| Cagrilintide 2.4 mg: Placebo | No concerns | Some concerns | No concerns | Some concerns | Major concerns | No concerns | Low | Reporting bias, Imprecision, Heterogeneity |
| Tirzepatide 10 mg: Placebo | No concerns | No concerns | No concerns | Some concerns | Major concerns | No concerns | Low | Imprecision, Heterogeneity |
| Oral Semaglutide 14 mg: Placebo | Major concerns | Some concerns | Some concerns | Major concerns | Major concerns | No concerns | Very low | Within-study bias, Reporting bias, Indirectness, Imprecision, Heterogeneity |
| Oral Semaglutide 50 mg: Placebo | No concerns | Some concerns | No concerns | Some concerns | Major concerns | No concerns | Low | Reporting bias, Imprecision, Heterogeneity |
| Cagrilintide 2.4 mg-Semaglutide 2.4 mg: Tirzepatide 15 mg | No concerns | No concerns | No concerns | Major concerns | Major concerns | No concerns | Very low | Imprecision, Heterogeneity |
| Cagrilintide 4.5 mg-Semaglutide 2.4 mg: Tirzepatide 15 mg | No concerns | No concerns | No concerns | Major concerns | Major concerns | No concerns | Very low | Imprecision, Heterogeneity |
| Semaglutide 2.4 mg: Tirzepatide 15 mg | Some concerns | No concerns | No concerns | Some concerns | Major concerns | No concerns | Low | Within-study bias, Imprecision, Heterogeneity |
| Oral Semaglutide 50 mg: Tirzepatide 15 mg | No concerns | Some concerns | No concerns | Major concerns | Major concerns | No concerns | Very low | Reporting bias, Imprecision, Heterogeneity |
| Cagrilintide 2.4 mg-Semaglutide 2.4 mg: Semaglutide 2.4 mg | No concerns | Some concerns | No concerns | Some concerns | Major concerns | No concerns | Low | Reporting bias, Imprecision, Heterogeneity |
| Cagrilintide 2.4 mg-Semaglutide 2.4 mg: Oral Semaglutide 50 mg | No concerns | Some concerns | No concerns | Major concerns | Major concerns | No concerns | Very low | Reporting bias, Imprecision, Heterogeneity |
| Cagrilintide 4.5 mg-Semaglutide 2.4 mg: Semaglutide 2.4 mg | No concerns | Some concerns | No concerns | Some concerns | Major concerns | No concerns | Low | Reporting bias, Imprecision, Heterogeneity |
| Cagrilintide 4.5 mg-Semaglutide 2.4 mg: Oral Semaglutide 50 mg | No concerns | Some concerns | No concerns | Major concerns | Major concerns | No concerns | Very low | Reporting bias, Imprecision, Heterogeneity |
| Oral Semaglutide 50 mg: Semaglutide 2.4 mg | No concerns | Some concerns | No concerns | Major concerns | Major concerns | No concerns | Very low | Reporting bias, Imprecision, Heterogeneity |

# **Supplement S10. Forest plot.**

**Figure S10.1: Forest Plot of Proportion of patients achieving ≥ 5% weight loss**

**
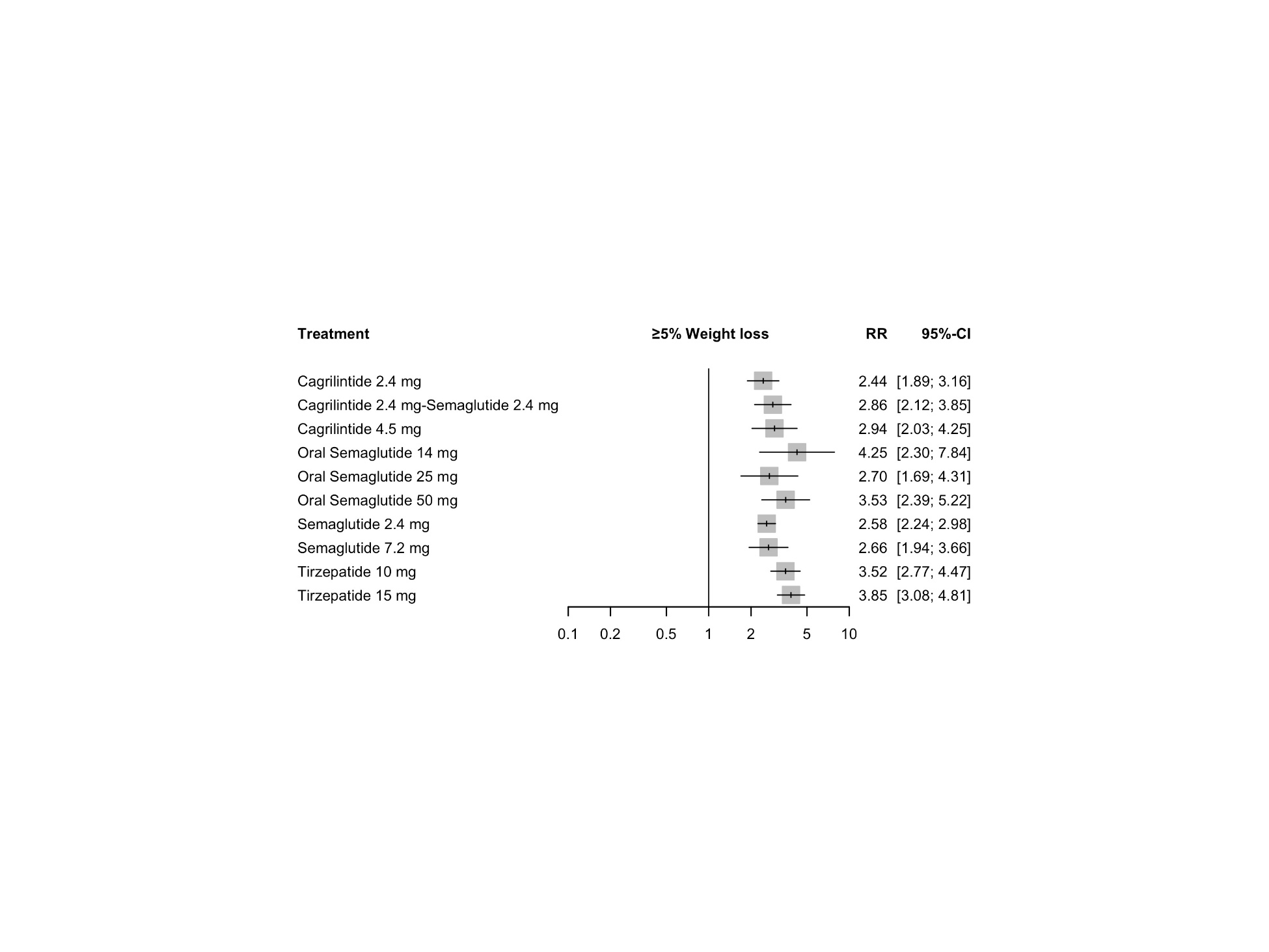
**

**Figure S10.2: Forest Plot of Proportion of patients achieving ≥ 10% weight loss**

**
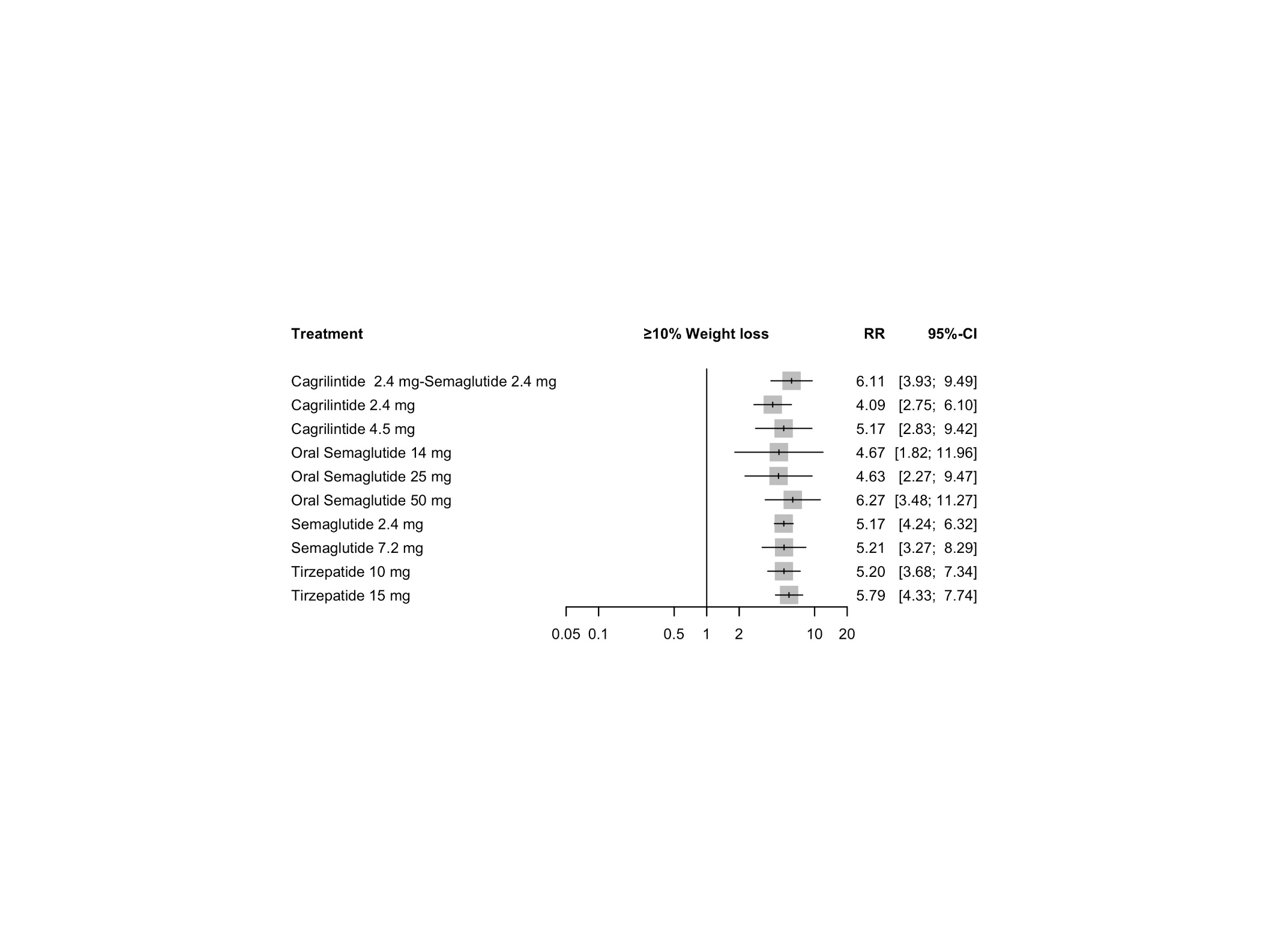
**

**Figure S10.3: Forest Plot of Proportion of patients achieving ≥ 15% weight loss**

**
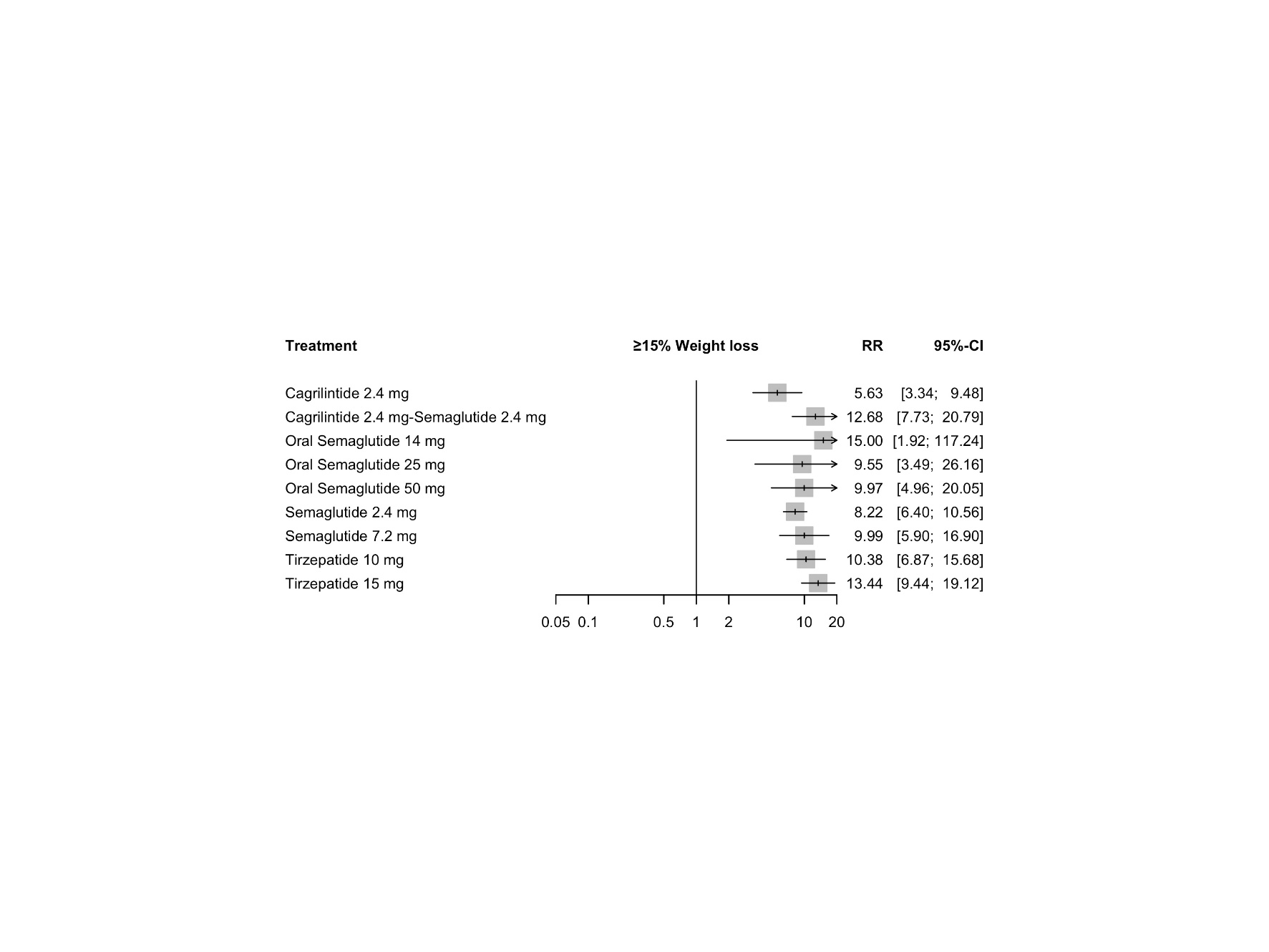
**

**Figure S10.4: Forest Plot of Proportion of patients achieving ≥ 20% weight loss**


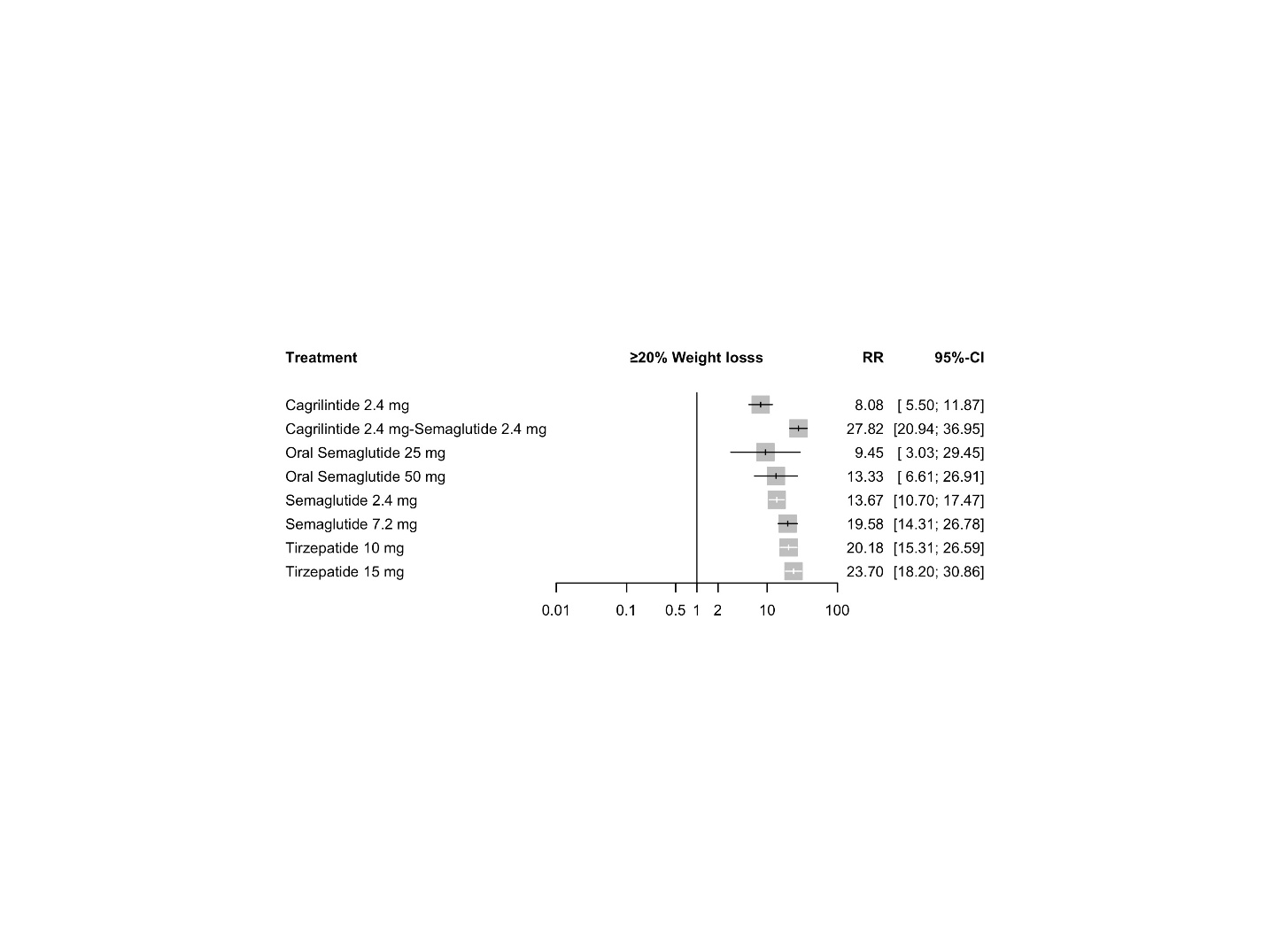


# **Supplement S11. Sensitivity Analysis**

## **Table S11.1: Percent Change in Body Weight Sensitivity Analysis**

| Intervention | Main Analysis MD (95% CI) | Excluding High RoB MD (95% CI) | Excluding Small Studies MD (95% CI) | I² Main (%) | I² High RoB (%) | I² Small (%) |
| --- | --- | --- | --- | --- | --- | --- |
| Tirzepatide 15 mg | −17.97 (−19.72, −16.21) | −18.03 (−19.94, −16.12) | −17.95 (−19.71, −16.20) | 89.4 | 89.5 | 89.9 |
| Cagrilintide 2.4 mg-Semaglutide 2.4 mg | −17.46 (−20.55, −14.37) | −17.44 (−20.56, −14.32) | −16.88 (−20.47, −13.29) | 89.4 | 89.5 | 89.9 |
| Cagrilintide 4.5 mg- Semaglutide 2.4 mg | −17.84 (−23.71, −11.98) | −17.82 (−23.72, −11.93) | — | 89.4 | 89.5 | — |
| Tirzepatide 10 mg | −14.77 (−17.29, −12.25) | −14.80 (−17.36, −12.24) | −14.76 (−17.28, −12.24) | 89.4 | 89.5 | 89.9 |
| Semaglutide 7.2 mg | −14.66 (−18.48, −10.84) | −14.64 (−18.50, −10.79) | −14.62 (−18.44, −10.79) | 89.4 | 89.5 | 89.9 |
| Semaglutide 2.4 mg | −11.41 (−12.74, −10.08) | −11.38 (−12.78, −9.98) | −11.32 (−12.72, −9.92) | 89.4 | 89.5 | 89.9 |
| Oral Semaglutide 25 mg | −11.40 (−16.16, −6.64) | −11.40 (−16.19, −6.61) | −11.40 (−16.16, −6.64) | 89.4 | 89.5 | 89.9 |
| Oral Semaglutide 50 mg | −11.24 (−14.29, −8.19) | −11.24 (−14.31, −8.17) | −12.70 (−17.02, −8.38) | 89.4 | 89.5 | 89.9 |
| Oral Semaglutide 14 mg | −8.06 (−12.51, −3.61) | — | — | 89.4 | — | — |
| Cagrilintide 4.5 mg | −8.30 (−12.34, −4.25) | −8.29 (−12.37, −4.21) | −8.23 (−12.28, −4.18) | 89.4 | 89.5 | 89.9 |
| Cagrilintide 2.4 mg | −7.69 (−10.52, −4.87) | −7.68 (−10.53, −4.83) | −7.56 (−10.40, −4.71) | 89.4 | 89.5 | 89.9 |

## **Table S11.2: Change in Waist Circumference Sensitivity Analysis**

| Intervention | Main Analysis MD (95% CI) | Excluding High RoB MD (95% CI) | Excluding Small Studies MD (95% CI) | I² Main (%) | I² High RoB (%) | I² Small (%) |
| --- | --- | --- | --- | --- | --- | --- |
| Tirzepatide 15 mg | −14.07 (−15.73, −12.41) | −14.16 (−16.04, −12.28) | −14.06 (−15.73, −12.39) | 83.9 | 84.4 | 84.5 |
| Cagrilintide 2.4 mg-Semaglutide 2.4 mg | −12.66 (−15.47, −9.84) | −12.64 (−15.49, −9.79) | −12.63 (−15.46, −9.80) | 83.9 | 84.4 | 84.5 |
| Tirzepatide 10 mg | −11.62 (−13.65, −9.60) | −11.66 (−13.75, −9.58) | −11.61 (−13.65, −9.58) | 83.9 | 84.4 | 84.5 |
| Semaglutide 7.2 mg | −11.49 (−14.48, −8.51) | −11.48 (−14.51, −8.45) | −11.47 (−14.48, −8.46) | 83.9 | 84.4 | 84.5 |
| Oral Semaglutide 50 mg | −10.00 (−13.34, −6.66) | −10.00 (−13.38, −6.62) | −10.00 (−13.36, −6.64) | 83.9 | 84.4 | 84.5 |
| Oral Semaglutide 25 mg | −9.50 (−13.69, −5.31) | −9.50 (−13.72, −5.28) | −9.50 (−13.70, −5.30) | 83.9 | 84.4 | 84.5 |
| Semaglutide 2.4 mg | −8.35 (−9.45, −7.24) | −8.31 (−9.48, −7.15) | −8.29 (−9.45, −7.13) | 83.9 | 84.4 | 84.5 |
| Oral Semaglutide 14 mg | −6.36 (−9.78, −2.94) | — | — | 83.9 | — | — |
| Cagrilintide 4.5 mg | −5.46 (−8.81, −2.11) | −5.46 (−8.84, −2.07) | −5.45 (−8.82, −2.09) | 83.9 | 84.4 | 84.5 |
| Cagrilintide 2.4 mg | −5.52 (−7.78, −3.26) | −5.51 (−7.80, −3.22) | −5.50 (−7.78, −3.23) | 83.9 | 84.4 | 84.5 |

## **Table S11.3: Change BMI Sensitivity Analysis**

| Intervention | Main Analysis MD (95% CI) | Excluding High RoB MD (95% CI) | Excluding Small Studies MD (95% CI) | I² Main (%) | I² High RoB (%) | I² Small (%) |
| --- | --- | --- | --- | --- | --- | --- |
| Tirzepatide 15 mg | −7.69 (−8.42, −6.97) | −8.11 (−8.89, −7.33) | −7.71 (−8.46, −6.96) | 73.4 | 65.5 | 75.9 |
| Cagrilintide 2.4 mg-Semaglutide 2.4 mg | −6.69 (−7.65, −5.72) | −6.63 (−7.46, −5.80) | −6.72 (−7.73, −5.70) | 73.4 | 65.5 | 75.9 |
| Semaglutide 7.2 mg | −5.74 (−6.76, −4.72) | −5.69 (−6.58, −4.80) | −5.77 (−6.83, −4.71) | 73.4 | 65.5 | 75.9 |
| Tirzepatide 10 mg | −5.50 (−6.68, −4.31) | −5.70 (−6.79, −4.61) | −5.51 (−6.73, −4.28) | 73.4 | 65.5 | 75.9 |
| Oral Semaglutide 50 mg | −4.70 (−5.84, −3.56) | −4.70 (−5.69, −3.71) | −4.70 (−5.88, −3.52) | 73.4 | 65.5 | 75.9 |
| Semaglutide 2.4 mg | −4.37 (−4.84, −3.91) | −4.25 (−4.68, −3.83) | −4.43 (−4.95, −3.91) | 73.4 | 65.5 | 75.9 |
| Oral Semaglutide 25 mg | −4.30 (−5.64, −2.96) | −4.30 (−5.52, −3.08) | −4.30 (−5.68, −2.92) | 73.4 | 65.5 | 75.9 |
| Cagrilintide 2.4 mg | −3.09 (−4.05, −2.12) | −3.03 (−3.86, −2.20) | −3.12 (−4.13, −2.10) | 73.4 | 65.5 | 75.9 |
|  |  |  |  |  |  |  |

## **Table S11.4: Change in Absolute Weight Sensitivity Analysis**

| Intervention | Main Analysis MD (95% CI) | Excluding High RoB MD (95% CI) | Excluding Small Studies MD (95% CI) | I² Main (%) | I² High RoB (%) | I² Small (%) |
| --- | --- | --- | --- | --- | --- | --- |
| Tirzepatide 15 mg | −21.41 (−23.38, −19.45) | −22.41 (−24.67, −20.14) | −21.50 (−23.43, −19.57) | 70.4 | 67.2 | 69.9 |
| Cagrilintide 4.5 mg-Semaglutide 2.4 mg | −19.12 (−23.22, −15.02) | −18.95 (−22.92, −14.97) | — | 70.4 | 67.2 | — |
| Cagrilintide 2.4 mg-Semaglutide 2.4 mg | −18.36 (−20.57, −16.16) | −18.23 (−20.31, −16.16) | −18.19 (−20.58, −15.80) | 70.4 | 67.2 | 69.9 |
| Semaglutide 7.2 mg | −16.10 (−18.88, −13.33) | −16.00 (−18.62, −13.38) | −16.22 (−18.94, −13.50) | 70.4 | 67.2 | 69.9 |
| Tirzepatide 10 mg | −15.56 (−18.76, −12.36) | −16.05 (−19.18, −12.93) | −15.60 (−18.74, −12.46) | 70.4 | 67.2 | 69.9 |
| Semaglutide 2.4 mg | −12.28 (−13.38, −11.18) | −12.06 (−13.13, −10.98) | −12.52 (−13.67, −11.37) | 70.4 | 67.2 | 69.9 |
| Oral Semaglutide 25 mg | −12.00 (−15.69, −8.31) | −12.00 (−15.54, −8.46) | −12.00 (−15.63, −8.37) | 70.4 | 67.2 | 69.9 |
| Oral Semaglutide 50 mg | −11.53 (−13.69, −9.36) | −11.52 (−13.56, −9.49) | −13.00 (−16.01, −9.99) | 70.4 | 67.2 | 69.9 |
| Cagrilintide 4.5 mg | −8.85 (−11.79, −5.91) | −8.82 (−11.59, −6.04) | −8.86 (−11.74, −5.99) | 70.4 | 67.2 | 69.9 |
| Cagrilintide 2.4 mg | −8.20 (−10.21, −6.19) | −8.13 (−10.02, −6.24) | −8.22 (−10.20, −6.25) | 70.4 | 67.2 | 69.9 |

## **Table S11.5: Proportion of patients achieving ≥ 5% weight loss**

| Intervention | Main Analysis RR (95% CI) | Excluding High RoB RR (95% CI) | Excluding Small Studies RR (95% CI) | I² Main (%) | I² High RoB (%) | I² Small (%) |
| --- | --- | --- | --- | --- | --- | --- |
| Tirzepatide 15 mg | 3.85 (3.08, 4.81) | 3.85 (3.08, 4.81) | 3.84 (3.08, 4.80) | 83.0 | 83.0 | 83.7 |
| Oral Semaglutide 14 mg | 4.25 (2.31, 7.84) | — | — | 83.0 | — | — |
| Tirzepatide 10 mg | 3.52 (2.77, 4.47) | 3.52 (2.77, 4.47) | 3.52 (2.77, 4.46) | 83.0 | 83.0 | 83.7 |
| Oral Semaglutide 50 mg | 3.53 (2.39, 5.22) | 3.53 (2.39, 5.22) | 3.53 (2.39, 5.20) | 83.0 | 83.0 | 83.7 |
| Cagrilintide 2.4 mg-Semaglutide 2.4 mg | 2.86 (2.12, 3.85) | 2.86 (2.12, 3.85) | 2.82 (2.10, 3.79) | 83.0 | 83.0 | 83.7 |
| Cagrilintide 4.5 mg | 2.94 (2.03, 4.25) | 2.94 (2.03, 4.25) | 2.92 (2.02, 4.21) | 83.0 | 83.0 | 83.7 |
| Oral Semaglutide 25 mg | 2.70 (1.69, 4.31) | 2.70 (1.69, 4.31) | 2.70 (1.70, 4.29) | 83.0 | 83.0 | 83.7 |
| Semaglutide 7.2 mg | 2.66 (1.94, 3.66) | 2.66 (1.94, 3.66) | 2.62 (1.91, 3.60) | 83.0 | 83.0 | 83.7 |
| Semaglutide 2.4 mg | 2.58 (2.24, 2.98) | 2.58 (2.24, 2.98) | 2.52 (2.18, |  |  |  |

## **Table S11.6: Proportion of patients achieving ≥ 10% weight loss**

| Intervention | Main Analysis RR (95% CI) | Excluding High RoB RR (95% CI) | Excluding Small Studies RR (95% CI) | I² Main (%) | I² High RoB (%) | I² Small (%) |
| --- | --- | --- | --- | --- | --- | --- |
| Cagrilintide 2.4 mg-Semaglutide 2.4 mg | 6.11 (3.93, 9.49) | 5.85 (3.88, 8.81) | 6.06 (3.89, 9.43) | 82.2 | 77.2 | 83.3 |
| Oral Semaglutide 50 mg | 6.27 (3.48, 11.27) | 6.27 (3.60, 10.92) | 6.27 (3.48, 11.29) | 82.2 | 77.2 | 83.3 |
| Tirzepatide 15 mg | 5.79 (4.33, 7.74) | 7.01 (5.06, 9.72) | 5.77 (4.31, 7.71) | 82.2 | 77.2 | 83.3 |
| Semaglutide 7.2 mg | 5.21 (3.27, 8.29) | 4.95 (3.20, 7.65) | 5.15 (3.23, 8.22) | 82.2 | 77.2 | 83.3 |
| Tirzepatide 10 mg | 5.20 (3.68, 7.34) | 5.96 (4.20, 8.45) | 5.18 (3.66, 7.33) | 82.2 | 77.2 | 83.3 |
| Cagrilintide 4.5 mg | 5.17 (2.83, 9.42) | 5.02 (2.84, 8.87) | 5.14 (2.81, 9.40) | 82.2 | 77.2 | 83.3 |
| Semaglutide 2.4 mg | 5.17 (4.24, 6.32) | 4.75 (3.89, 5.81) | 5.09 (4.15, 6.25) | 82.2 | 77.2 | 83.3 |
| Oral Semaglutide 25 mg | 4.63 (2.27, 9.47) | 4.63 (2.33, 9.22) | 4.63 (2.26, 9.48) | 82.2 | 77.2 | 83.3 |
| Oral Semaglutide 14 mg | 4.67 (1.82, 11.96) | — | — | 82.2 | — | — |

## **Table S11.7: Proportion of patients achieving ≥ 15% weight loss**

| Intervention | Main Analysis RR (95% CI) |  | Excluding High RoB RR (95% CI) | Excluding Small Studies RR (95% CI) | I² Main (%) | I² High RoB (%) | I² Small (%) |
| --- | --- | --- | --- | --- | --- | --- | --- |
| Tirzepatide 15 mg | 13.44 (9.44, 19.12) |  | 13.92 (8.83, 21.94) | 13.39 (9.39, 19.11) | 66.9 | 67.4 | 68.8 |
| Cagrilintide 2.4 mg + Semaglutide 2.4 mg | 12.68 (7.73, 20.79) |  | 12.68 (7.49, 21.48) | 12.58 (7.64, 20.73) | 66.9 | 67.4 | 68.8 |
| Oral Semaglutide 14 mg | 15.00 (1.92, 117.24) |  | — | — | 66.9 | — | — |
| Tirzepatide 10 mg | 10.38 (6.87, 15.68) |  | 10.68 (6.60, 17.27) | 10.35 (6.83, 15.69) | 66.9 | 67.4 | 68.8 |
| Semaglutide 7.2 mg | 9.99 (5.91, 16.90) |  | 9.98 (5.71, 17.46) | 9.90 (5.82, 16.83) | 66.9 | 67.4 | 68.8 |
| Oral Semaglutide 50 mg | 9.97 (4.96, 20.05) |  | 9.97 (4.83, 20.58) | 9.97 (4.94, 20.12) | 66.9 | 67.4 | 68.8 |
| Oral Semaglutide 25 mg | 9.55 (3.49, 26.16) |  | 9.55 (3.43, 26.64) | 9.55 (3.48, 26.22) | 66.9 | 67.4 | 68.8 |
| Semaglutide 2.4 mg | 8.22 (6.40, 10.56) |  | 8.22 (6.22, 10.85) | 8.12 (6.29, 10.48) | 66.9 | 67.4 | 68.8 |
| Cagrilintide 2.4 mg | 5.63 (3.34, 9.48) |  | 5.63 (3.24, 9.78) | 5.58 (3.30, 9.45) | 66.9 | 67.4 | 68.8 |

## **Table S11.8: Proportion of patients achieving ≥ 20% weight loss**

| Intervention | Main Analysis RR (95% CI) | Excluding High RoB RR (95% CI) | Excluding Small Studies RR (95% CI) | I² Main (%) | I² High RoB (%) | I² Small (%) |
| --- | --- | --- | --- | --- | --- | --- |
| Cagrilintide 2.4 mg-Semaglutide 2.4 mg | 27.82 (20.94, 36.95) | 28.91 (21.02, 39.76) | 27.78 (20.88, 36.95) | 0.0 | 0.0 | 0.0 |
| Tirzepatide 15 mg | 23.70 (18.20, 30.86) | 21.87 (14.68, 32.59) | 23.67 (18.15, 30.86) | 0.0 | 0.0 | 0.0 |
| Tirzepatide 10 mg | 20.18 (15.31, 26.59) | 18.66 (12.49, 27.89) | 20.16 (15.28, 26.59) | 0.0 | 0.0 | 0.0 |
| Semaglutide 7.2 mg | 19.58 (14.31, 26.78) | 20.40 (14.39, 28.91) | 19.55 (14.27, 26.78) | 0.0 | 0.0 | 0.0 |
| Semaglutide 2.4 mg | 13.67 (10.70, 17.47) | 14.28 (10.65, 19.15) | 13.65 (10.67, 17.47) | 0.0 | 0.0 | 0.0 |
| Oral Semaglutide 50 mg | 13.34 (6.61, 26.91) | 13.34 (6.61, 26.91) | 13.34 (6.61, 26.91) | 0.0 | 0.0 | 0.0 |
| Oral Semaglutide 25 mg | 9.45 (3.03, 29.45) | 9.45 (3.03, 29.45) | 9.45 (3.03, 29.45) | 0.0 | 0.0 | 0.0 |
| Cagrilintide 2.4 mg | 8.08 (5.50, 11.87) | 8.39 (5.56, 12.66) | 8.07 (5.48, 11.86) | 0.0 | 0.0 | 0.0 |

## **Table S11.9: Change in HDL Sensitivity Analysis**

| Intervention | Main Analysis MD (95% CI) | Excluding High RoB MD (95% CI) | Excluding Small Studies MD (95% CI) | I² Main (%) | I² High RoB (%) | I² Small (%) |
| --- | --- | --- | --- | --- | --- | --- |
| Cagrilintide 2.4 mg-Semaglutide 2.4 mg | 7.41 (5.78, 9.04) | 7.32 (5.64, 9.00) | 7.80 (5.95, 9.64) | 68.2 | 69.4 | 70.2 |
| Cagrilintide 4.5 mg-Semaglutide 2.4 mg | 6.80 (3.91, 9.69) | 6.68 (3.74, 9.62) | — | 68.2 | 69.4 | — |
| Tirzepatide 15 mg | 5.89 (4.89, 6.89) | 6.20 (5.00, 7.39) | 5.88 (4.88, 6.89) | 68.2 | 69.4 | 70.2 |
| Tirzepatide 10 mg | 4.95 (3.78, 6.11) | 5.14 (3.89, 6.39) | 4.94 (3.78, 6.11) | 68.2 | 69.4 | 70.2 |
| Oral Semaglutide 50 mg | 4.80 (1.91, 7.69) | 4.80 (1.87, 7.73) | 4.80 (1.91, 7.69) | 68.2 | 69.4 | 70.2 |
| Cagrilintide 2.4 mg | 2.58 (0.76, 4.40) | 2.50 (0.63, 4.38) | 2.70 (0.85, 4.54) | 68.2 | 69.4 | 70.2 |
| Semaglutide 2.4 mg | 2.33 (1.56, 3.10) | 2.19 (1.34, 3.04) | 2.29 (1.52, 3.07) | 68.2 | 69.4 | 70.2 |
| Oral Semaglutide 14 mg | 0.31 (−1.81, 2.43) | — | — | 68.2 | — | — |

## **Table S11.10: Any Adverse Event Sensitivity Analysis**

| Intervention | Main Analysis RR (95% CI) | Excluding High RoB RR (95% CI) | Excluding Small Studies RR (95% CI) | I² Main (%) | I² High RoB (%) | I² Small (%) |
| --- | --- | --- | --- | --- | --- | --- |
| Oral Semaglutide 14 mg | 1.95 (1.30, 2.92) | — | — | 40.4 | — | — |
| Cagrilintide 4.5 mg | 1.22 (1.07, 1.40) | 1.22 (1.07, 1.38) | 1.22 (1.08, 1.39) | 40.4 | 32.4 | 39.9 |
| Tirzepatide 10 mg | 1.14 (1.07, 1.21) | 1.15 (1.09, 1.22) | 1.14 (1.07, 1.21) | 40.4 | 32.4 | 39.9 |
| Tirzepatide 15 mg | 1.09 (1.05, 1.14) | 1.11 (1.06, 1.17) | 1.09 (1.05, 1.14) | 40.4 | 32.4 | 39.9 |
| Semaglutide 7.2 mg | 1.11 (1.02, 1.20) | 1.10 (1.02, 1.18) | 1.11 (1.03, 1.19) | 40.4 | 32.4 | 39.9 |
| Cagrilintide 2.4 mg-Semaglutide 2.4 mg | 1.10 (1.04, 1.17) | 1.10 (1.04, 1.16) | 1.11 (1.05, 1.17) | 40.4 | 32.4 | 39.9 |
| Oral Semaglutide 50 mg | 1.09 (1.00, 1.18) | 1.09 (1.01, 1.17) | 1.07 (0.99, 1.16) | 40.4 | 32.4 | 39.9 |
| Oral Semaglutide 25 mg | 1.09 (0.97, 1.21) | 1.09 (0.98, 1.21) | 1.09 (0.98, 1.21) | 40.4 | 32.4 | 39.9 |
| Semaglutide 2.4 mg | 1.05 (1.02, 1.08) | 1.04 (1.01, 1.07) | 1.05 (1.02, 1.08) | 40.4 | 32.4 | 39.9 |
| Cagrilintide 4.5 mg-Semaglutide 2.4 mg | 1.02 (0.82, 1.26) | 1.01 (0.82, 1.24) | — | 40.4 | 32.4 | — |
| Cagrilintide 2.4 mg | 1.02 (0.95, 1.10) | 1.02 (0.95, 1.09) | 1.03 (0.96, 1.10) | 40.4 | 32.4 | 39.9 |

## **Table S11.11: Serious Adverse Events Sensitivity Analysis**

| Intervention | Main Analysis RR (95% CI) | Excluding High RoB RR (95% CI) | Excluding Small Studies RR (95% CI) | I² Main (%) | I² High RoB (%) | I² Small (%) |
| --- | --- | --- | --- | --- | --- | --- |
| Cagrilintide 4.5 mg-Semaglutide 2.4 mg | 2.13 (0.20, 22.45) | 2.18 (0.21, 23.08) | — | 41.5 | 43.0 | — |
| Cagrilintide 2.4 mg-Semaglutide 2.4 mg | 1.76 (1.06, 2.91) | 1.78 (1.06, 2.98) | 1.74 (1.01, 2.99) | 41.5 | 43.0 | 48.9 |
| Cagrilintide 4.5 mg | 1.66 (0.43, 6.35) | 1.67 (0.43, 6.43) | 1.65 (0.43, 6.42) | 41.5 | 43.0 | 48.9 |
| Cagrilintide 2.4 mg | 1.54 (0.86, 2.73) | 1.55 (0.86, 2.79) | 1.52 (0.84, 2.78) | 41.5 | 43.0 | 48.9 |
| Tirzepatide 10 mg | 1.11 (0.69, 1.80) | 1.07 (0.65, 1.75) | 1.11 (0.68, 1.83) | 41.5 | 43.0 | 48.9 |
| Oral Semaglutide 50 mg | 1.10 (0.56, 2.17) | 1.10 (0.55, 2.20) | 1.10 (0.54, 2.24) | 41.5 | 43.0 | 48.9 |
| Semaglutide 2.4 mg | 1.09 (0.86, 1.37) | 1.13 (0.88, 1.44) | 1.07 (0.84, 1.38) | 41.5 | 43.0 | 48.9 |
| Tirzepatide 15 mg | 1.01 (0.72, 1.43) | 0.94 (0.64, 1.37) | 1.02 (0.71, 1.45) | 41.5 | 43.0 | 48.9 |
| Semaglutide 7.2 mg | 0.85 (0.47, 1.54) | 0.87 (0.47, 1.60) | 0.84 (0.45, 1.58) | 41.5 | 43.0 | 48.9 |
| Oral Semaglutide 25 mg | 0.44 (0.16, 1.25) | 0.44 (0.16, 1.26) | 0.44 (0.15, 1.28) | 41.5 | 43.0 | 48.9 |

## **Table S11.12: GI Adverse Events Sensitivity Analysis**

| Intervention | Main Analysis RR (95% CI) | Excluding High RoB RR (95% CI) | Excluding Small Studies RR (95% CI) | I² Main (%) | I² High RoB (%) | I² Small (%) |
| --- | --- | --- | --- | --- | --- | --- |
| Tirzepatide 15 mg | 1.91 (0.89, 4.11) | — | 1.89 (0.85, 4.19) | 63.3 | — | 76.4 |
| Oral Semaglutide 50 mg | 1.91 (1.09, 3.36) | 1.91 (1.09, 3.36) | 2.39 (0.81, 7.10) | 63.3 | 63.3 | 76.4 |
| Cagrilintide 2.4 mg-Semaglutide 2.4 mg | 1.86 (1.47, 2.34) | 1.86 (1.47, 2.34) | 1.87 (1.37, 2.55) | 63.3 | 63.3 | 76.4 |
| Semaglutide 7.2 mg | 1.74 (1.31, 2.31) | 1.74 (1.31, 2.31) | 1.73 (1.24, 2.40) | 63.3 | 63.3 | 76.4 |
| Oral Semaglutide 25 mg | 1.74 (0.36, 8.45) | 1.74 (0.36, 8.45) | 1.74 (0.35, 8.55) | 63.3 | 63.3 | 76.4 |
| Cagrilintide 4.5 mg-Semaglutide 2.4 mg | 1.64 (1.07, 2.52) | 1.64 (1.07, 2.52) | — | 63.3 | 63.3 | — |
| Cagrilintide 4.5 mg | 1.78 (1.24, 2.56) | 1.78 (1.24, 2.56) | 1.79 (1.19, 2.69) | 63.3 | 63.3 | 76.4 |
| Semaglutide 2.4 mg | 1.57 (1.34, 1.83) | 1.57 (1.34, 1.83) | 1.55 (1.28, 1.87) | 63.3 | 63.3 | 76.4 |
| Cagrilintide 2.4 mg | 1.33 (1.05, 1.69) | 1.33 (1.05, 1.69) | 1.34 (1.01, 1.76) | 63.3 | 63.3 | 76.4 |

## **Table S11.13: GI Adverse Events Sensitivity Analysis**

| Intervention | Main Analysis RR (95% CI) | Excluding High RoB RR (95% CI) | Excluding Small Studies RR (95% CI) | I² Main (%) | I² High RoB (%) | I² Small (%) |
| --- | --- | --- | --- | --- | --- | --- |
| Oral Semaglutide 14 mg | 4.00 (0.45, 35.67) | 4.00 (0.45, 35.67) | 4.00 (0.45, 35.76) | 18.3 | 18.3 | 20.1 |
| Cagrilintide 4.5 mg-Semaglutide 2.4 mg | 3.21 (0.31, 33.22) | 3.21 (0.31, 33.22) | 3.18 (0.31, 32.95) | 18.3 | 18.3 | 20.1 |
| Semaglutide 7.2 mg | 3.09 (1.45, 6.59) | 3.09 (1.45, 6.59) | — | 18.3 | 18.3 | — |
| Cagrilintide 2.4 mg-Semaglutide 2.4 mg | 2.22 (1.39, 3.54) | 2.22 (1.39, 3.54) | 2.21 (1.37, 3.55) | 18.3 | 18.3 | 20.1 |
| Tirzepatide 10 mg | 2.10 (1.30, 3.38) | 2.10 (1.30, 3.38) | 2.09 (1.29, 3.38) | 18.3 | 18.3 | 20.1 |
| Semaglutide 1 mg | 2.00 (0.19, 21.43) | 2.00 (0.19, 21.43) | 2.00 (0.19, 21.48) | 18.3 | 18.3 | 20.1 |
| Tirzepatide 15 mg | 1.99 (1.39, 2.85) | 1.99 (1.39, 2.85) | 1.98 (1.38, 2.84) | 18.3 | 18.3 | 20.1 |
| Semaglutide 2.4 mg | 1.98 (1.57, 2.50) | 1.98 (1.57, 2.50) | 1.94 (1.53, 2.47) | 18.3 | 18.3 | 20.1 |
| Oral Semaglutide 50 mg | 1.58 (0.72, 3.46) | 1.58 (0.72, 3.46) | 1.58 (0.71, 3.49) | 18.3 | 18.3 | 20.1 |
| Oral Semaglutide 25 mg | 1.16 (0.43, 3.12) | 1.16 (0.43, 3.12) | 1.16 (0.43, 3.14) | 18.3 | 18.3 | 20.1 |
| Cagrilintide 2.4 mg | 1.14 (0.56, 2.31) | 1.14 (0.56, 2.31) | 1.14 (0.56, 2.31) | 18.3 | 18.3 | 20.1 |
| Cagrilintide 4.5 mg | 0.23 (0.03, 1.94) | 0.23 (0.03, 1.94) | 0.23 (0.03, |  |  |  |

# **S12. PRISMA Check list.**

| **Section and Topic** | **Item #** | **Checklist item** | **Location where item is reported** |
| --- | --- | --- | --- |
| **TITLE** | | |  |
| Title | 1 | Identify the report as a systematic review. | 1 |
| **ABSTRACT** | | |  |
| Abstract | 2 | See the PRISMA 2020 for Abstracts checklist. |  |
| **INTRODUCTION** | | |  |
| Rationale | 3 | Describe the rationale for the review in the context of existing knowledge. |  |
| Objectives | 4 | Provide an explicit statement of the objective(s) or question(s) the review addresses. |  |
| **METHODS** | | |  |
| Eligibility criteria | 5 | Specify the inclusion and exclusion criteria for the review and how studies were grouped for the syntheses. |  |
| Information sources | 6 | Specify all databases, registers, websites, organisations, reference lists and other sources searched or consulted to identify studies. Specify the date when each source was last searched or consulted. |  |
| Search strategy | 7 | Present the full search strategies for all databases, registers and websites, including any filters and limits used. |  |
| Selection process | 8 | Specify the methods used to decide whether a study met the inclusion criteria of the review, including how many reviewers screened each record and each report retrieved, whether they worked independently, and if applicable, details of automation tools used in the process. |  |
| Data collection process | 9 | Specify the methods used to collect data from reports, including how many reviewers collected data from each report, whether they worked independently, any processes for obtaining or confirming data from study investigators, and if applicable, details of automation tools used in the process. |  |
| Data items | 10a | List and define all outcomes for which data were sought. Specify whether all results that were compatible with each outcome domain in each study were sought (e.g. for all measures, time points, analyses), and if not, the methods used to decide which results to collect. |  |
|  | 10b | List and define all other variables for which data were sought (e.g. participant and intervention characteristics, funding sources). Describe any assumptions made about any missing or unclear information. |  |
| Study risk of bias assessment | 11 | Specify the methods used to assess risk of bias in the included studies, including details of the tool(s) used, how many reviewers assessed each study and whether they worked independently, and if applicable, details of automation tools used in the process. | 1 |
| Effect measures | 12 | Specify for each outcome the effect measure(s) (e.g. risk ratio, mean difference) used in the synthesis or presentation of results. | 5,6 |
| Synthesis methods | 13a | Describe the processes used to decide which studies were eligible for each synthesis (e.g. tabulating the study intervention characteristics and comparing against the planned groups for each synthesis (item #5)). | 5 |
|  | 13b | Describe any methods required to prepare the data for presentation or synthesis, such as handling of missing summary statistics, or data conversions. | 5,6 |
|  | 13c | Describe any methods used to tabulate or visually display results of individual studies and syntheses. | 7 |
|  | 13d | Describe any methods used to synthesize results and provide a rationale for the choice(s). If meta-analysis was performed, describe the model(s), method(s) to identify the presence and extent of statistical heterogeneity, and software package(s) used. | 6,7 |
|  | 13e | Describe any methods used to explore possible causes of heterogeneity among study results (e.g. subgroup analysis, meta-regression). | 6,7 |
|  | 13f | Describe any sensitivity analyses conducted to assess robustness of the synthesized results. | 6,7 |
| Reporting bias assessment | 14 | Describe any methods used to assess risk of bias due to missing results in a synthesis (arising from reporting biases). | 6 |
| Certainty assessment | 15 | Describe any methods used to assess certainty (or confidence) in the body of evidence for an outcome. | 6 |
| **RESULTS** | | |  |
| Study selection | 16a | Describe the results of the search and selection process, from the number of records identified in the search to the number of studies included in the review, ideally using a flow diagram. | 5 |
|  | 16b | Cite studies that might appear to meet the inclusion criteria, but which were excluded, and explain why they were excluded. | 9 |
| Study characteristics | 17 | Cite each included study and present its characteristics. | Supplement S3 |
| Risk of bias in studies | 18 | Present assessments of risk of bias for each included study. | Supplement S5 |
| Results of individual studies | 19 | For all outcomes, present, for each study: (a) summary statistics for each group (where appropriate) and (b) an effect estimate and its precision (e.g. confidence/credible interval), ideally using structured tables or plots. | 7 |
| Results of syntheses | 20a | For each synthesis, briefly summarise the characteristics and risk of bias among contributing studies. | 9,9 |
|  | 20b | Present results of all statistical syntheses conducted. If meta-analysis was done, present for each the summary estimate and its precision (e.g. confidence/credible interval) and measures of statistical heterogeneity. If comparing groups, describe the direction of the effect. | 7,8 |
|  | 20c | Present results of all investigations of possible causes of heterogeneity among study results. | 7 |
|  | 20d | Present results of all sensitivity analyses conducted to assess the robustness of the synthesized results. | 7 |
| Reporting biases | 21 | Present assessments of risk of bias due to missing results (arising from reporting biases) for each synthesis assessed. | 6 |
| Certainty of evidence | 22 | Present assessments of certainty (or confidence) in the body of evidence for each outcome assessed. | Supplement S9 |
| **DISCUSSION** | | |  |
| Discussion | 23a | Provide a general interpretation of the results in the context of other evidence. | 9-21 |
|  | 23b | Discuss any limitations of the evidence included in the review. | 23 |
|  | 23c | Discuss any limitations of the review processes used. | 23 |
|  | 23d | Discuss implications of the results for practice, policy, and future research. | 22,23 |
| **OTHER INFORMATION** | | |  |
| Registration and protocol | 24a | Provide registration information for the review, including register name and registration number, or state that the review was not registered. | 4 |
|  | 24b | Indicate where the review protocol can be accessed, or state that a protocol was not prepared. | 4 |
|  | 24c | Describe and explain any amendments to information provided at registration or in the protocol. | 4 |
| Support | 25 | Describe sources of financial or non-financial support for the review, and the role of the funders or sponsors in the review. | 1 |
| Competing interests | 26 | Declare any competing interests of review authors. | 1 |
| Availability of data, code and other materials | 27 | Report which of the following are publicly available and where they can be found: template data collection forms; data extracted from included studies; data used for all analyses; analytic code; any other materials used in the review. | 1 |
